# Characterization of ACTN4 as a novel antiviral target against SARS-CoV-2

**DOI:** 10.1038/s41392-024-01956-4

**Published:** 2024-09-18

**Authors:** Miao Zhu, Fang Huang, Huize Sun, Kunpeng Liu, Zhen Chen, Baocheng Yu, Haojie Hao, Haizhou Liu, Shuang Ding, Xueyan Zhang, Lishi Liu, Kui Zhang, Jierao Ren, Yi Liu, Haibin Liu, Chao Shan, Wuxiang Guan

**Affiliations:** 1 Center for Emerging Infectious Diseases, Wuhan Institute of Virology, Center for Biosafety Mega-Science, Chinese Academy of Sciences, Wuhan, Hubei 430071 China; 2 University of Chinese Academy of Sciences, Beijing, 100049 China; 3 Hubei Jiangxia Laboratory, Wuhan, Hubei 430200 China

**Keywords:** Cell biology, Senescence, Epigenetics

## Abstract

The various mutations in severe acute respiratory syndrome coronavirus 2 (SARS-CoV-2) pose a substantial challenge in mitigating the viral infectivity. The identification of novel host factors influencing SARS-CoV-2 replication holds potential for discovering new targets for broad-spectrum antiviral drugs that can combat future viral mutations. In this study, potential host factors regulated by SARS-CoV-2 infection were screened through different high-throughput sequencing techniques and further identified in cells. Subsequent analysis and experiments showed that the reduction of m6A modification level on *ACTN4* (Alpha-actinin-4) mRNA leads to a decrease in mRNA stability and translation efficiency, ultimately inhibiting ACTN4 expression. In addition, ACTN4 was demonstrated to target nsp12 for binding and characterized as a competitor for SARS-CoV-2 RNA and the RNA-dependent RNA polymerase complex, thereby impeding viral replication. Furthermore, two ACTN4 agonists, YS-49 and demethyl-coclaurine, were found to dose-dependently inhibit SARS-CoV-2 infection in both Huh7 cells and K18-hACE2 transgenic mice. Collectively, this study unveils the pivotal role of ACTN4 in SARS-CoV-2 infection, offering novel insights into the intricate interplay between the virus and host cells, and reveals two potential candidates for future anti-SARS-CoV-2 drug development.

## Introduction

Severe acute respiratory syndrome coronavirus 2 (SARS-CoV-2), the etiological agent of the ongoing global pandemic, poses an unprecedented public health threat and has left an indelible mark on the global economy.^1–3^ Even broad vaccination against SARS-CoV-2 provides immunity for a limited time, but the emerging SARS-CoV-2 variants are able to partially or completely evade the immune response obtained through vaccination or infection. Several anti-SARS-CoV-2 drugs were widely used for COVID-19 treatment, such as Paxlovid targeting the viral encoded 3CL protease and VV116 nucleoside drug targeting the viral encoded RNA-dependent RNA polymerase (RdRp) complex.^4–7^ However, no drug candidates were available to target the host factors to block the replication of SARS-CoV-2.

SARS-CoV-2 possesses a single-stranded positive-sense RNA genome of approximately 30 kb that harbors 14 open reading frames, which encode a large polymeric protein, four structural proteins, and nine accessory proteins.^8–11^ The SARS-CoV-2 RdRp, nsp12, consists of 932 amino acids and features N-terminal nidovirus RdRp-associated nucleotidyltransferase, interface, and C-terminal RdRp domains. Notably, nsp12 exhibits limited intrinsic activity, but its functionality is markedly enhanced in the presence of cofactors, nsp7 and nsp8.^12–17^ A transition model from primer enzyme to polymerase complex has been proposed, in which the nsp7-nsp8 complex first binds to viral RNA and then recruits nsp12 to form the polymerase complex.^18^ Recent studies also reveal that the viral RNA endonuclease nsp15 targets TBK1 to interfere its interaction with IRF3 and inhibit IFN-beta production, and potential candidate molecules are identified to target nsp15 for inhibition.^19,20^ The indispensable roles of the RdRp complex and other viral proteins in SARS-CoV-2 replication render them an attractive target for drug development and have prompted extensive investigations, particularly into their interactions with host factors.^7,21,22^

N6-methyladenosine (m6A) modification plays pivotal roles in the regulation of infection by various viruses, including influenza A virus (IAV), enterovirus 71, HIV-1, and SARS-CoV-2.^23–27^ The reversible m6A modification catalyzed by “writers”, “erasers”, and “readers”, is the most abundant modification on eukaryotic messenger RNAs (mRNAs).^28–31^ The “writers” encompass methyltransferase-like (METTL)3 and METTL14 alongside the splicing factor Wilms’ tumor-1-associating protein (WTAP). The catalytical subunit is METTL3, METTL14 facilitates mRNA substrate recognition, whereas WTAP mainly orchestrates their localization within nuclear speckles. KIAA1429, HAKAI, ZC3H13, and RBM15/15B are also characterized as additional cofactors.^32–35^ The m6A methylation can be enzymatically removed by “erasers”, such as obesity-associated protein (FTO) and AlkB homolog 5 (ALKBH5). m6A “readers” include YTH domain-containing proteins (YTHDF1/2/3 and YTHDC1/2), which play pivotal roles in RNA stability, nuclear export, mRNA splicing, and translation.^36–39^ Notably, the SARS-CoV-2 genome also harbors m6A modifications. Recent findings indicate that RdRp modulates the sumoylation and ubiquitination of METTL3 via direct interaction with METTL3, thereby up-regulating viral replication.^26^ Conversely, m6A modifications on SARS-CoV-2 RNA exerted a negative regulatory effect on viral replication in Huh7 cells.^27^ Additionally, SARS-CoV-2 infection triggers global alterations in the host m6A methylome, resulting in shifts in the localization and motifs of m6A methylation within mRNAs.^27,40,41^ Nevertheless, a comprehensive understanding of the mechanisms how SARS-CoV-2 infection affects host cell m6A modifications is lacking.

Many host factors participate in viral infection in both positive and negative roles.^42,43^ The host factor Alpha-actinin-4 (ACTN4) regulates the replication of different viruses.^44–46^ ACTN4 is an actin-binding protein belonging to the spectrin superfamily that plays roles in maintaining cytoskeletal stability and regulating cell motility.^47–53^ Despite its pivotal roles in cellular physiology, numerous studies have revealed that ACTN4 may influence viral replication in specific instances. In hepatitis C virus (HCV) infection, interactions between ACTN4 and the NS5B have been observed and may affect viral replication dynamics.^44^ In rotavirus infection, actin microfilament dynamics may be influenced by ACTN4, particularly during virus internalization and viral RNA synthesis at 6 h post-infection (hpi).^46^ Notably, ACTN4 interacts with the viral nucleoprotein upon IAV infection, and this interaction may affect the nuclear localization of the viral nucleoprotein or ribonucleoproteins, thereby playing a pivotal role in viral replication.^45^ However, comprehensive insights into the function and mechanism of ACTN4 in viral infection are lacking.

In the current study, the interplay between the host factors and viral proteins were analyzed by combined nanopore high-throughput sequencing and second-generation sequencing. The host factor ACTN4 was found to interact with viral RdRp, which impeded viral replication. Notably, two ACTN4 agonists were capable of inhibiting the replication of various SARS-CoV-2 strains both in vitro and in vivo. The results showed that the host factor ACTN4 holds the potential for antiviral candidate.

## Results

### SARS-CoV-2 infection suppresses ACTN4 expression in an epitranscriptomic manner

**Fig. 1:**
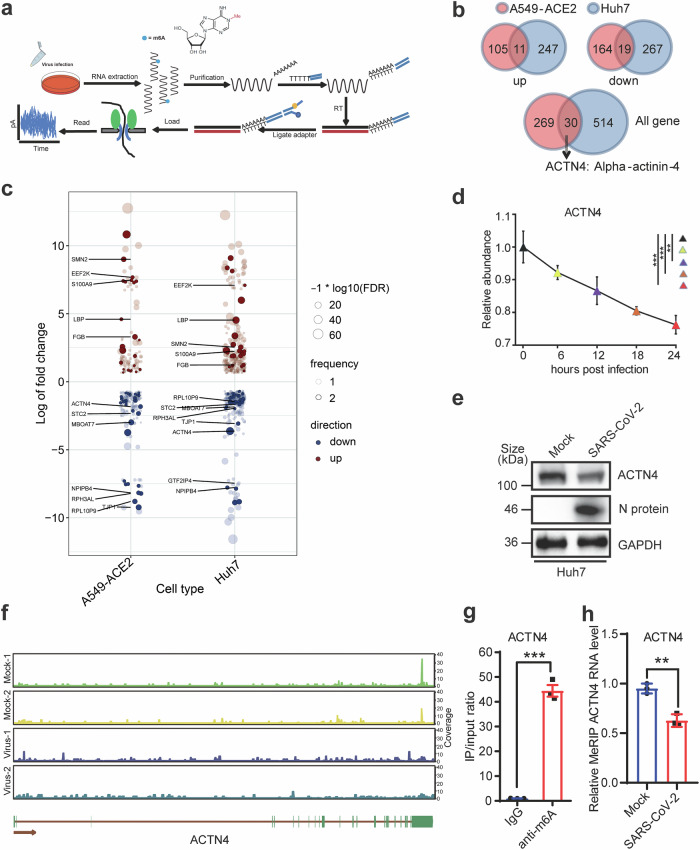
SARS-CoV-2 infection decreases ACTN4 expression in an epitranscriptomic manner. **a** Flow chart of Nanopore direct RNA sequencing. A549-ACE2 and Huh7 cells were infected with WT SARS-CoV-2 at MOI = 1, RNA was then collected at 48 h post-infection (hpi). After purification using oligo (dT), sequencing libraries were prepared and sequenced using a MinION device. **b** Overlap of differential gene expression in A549-ACE2 and Huh7 cells. **c** Map of differentially expressed genes. Sequencing data were statistically analyzed. Red dots represent up-regulated genes, green dots represent down-regulated genes. Dot size represents the degree of change. Log fold change based on the abundance of differential host genes. **d** Comparison of the relative abundance of *ACTN4* in Huh7 cells infected with WT SARS-CoV-2 at MOI = 1 at different time points within 24 h was analyzed by RT-qPCR. Data are means ± SEMs (*n* = 3). **P ≤ 0.01, ***P < 0.001, one-way ANOVA. **e** Western blot (WB) assays of ACTN4 expression in Huh7 cells at 48 hpi with SARS-CoV-2. GAPDH was used as the loading control. **f** Variation and distribution of m6A modifications on *ACTN4* mRNA. After performing MeRIP-seq via anti-m6A antibodies (Abs) and normalizing the m6A coverage from IP with the m6A coverage from Input, changes in m6A modification levels on *ACTN4* mRNAs in Huh7 cells with SARS-CoV-2 infection or not were presented. The top two panels presented normal cells ‘Mock’, the lower two panels presented infected cells ‘Virus’. **g**, **h** MeRIP-qPCR. RNAs from SARS-CoV-2-infected or mock cells were incubated with IgG or anti-m6A Abs, followed with immunoprecipitation and RT-qPCR. Data are means ± SEMs (*n* = 3). ***P < 0.001, unpaired Student’s *t*-test

SARS-CoV-2 infection induces substantial alterations in both the transcriptional and epitranscriptomic profiles of host genes, initiating diverse cellular responses.^27^ Total RNA were extracted from Huh7 or A549-ACE2 cells infected with wild-type SARS-CoV-2 for 48 h at MOI = 1, and subjected to nanopore high-throughput sequencing (Fig. 1a). After analyzing the sequencing results, the expression patterns of host genes in infected and uninfected cells were presented (Fig. 1b), and the differentially expressed genes were annotated (Fig. 1c) and clustered in cellular pathways (Supplementary Fig. 1a, b). After investigating the altered host factors in both cells, ACTN4 was largely down-regulated after SARS-CoV-2 infection. Additionally, after re-evaluating our previously published RNA-seq data,^54^ levels of *ACTN4* mRNA were decreased in a time course dependent manner in SARS-CoV-2 infected Huh7 cells (Fig. 1d). The *ACTN4* mRNA decrease was further confirmed by examining the protein and mRNA levels of *ACTN4* after SARS-CoV-2 infection in both cells (Fig. 1e, Supplementary Fig. 2a-c). Previous studies have suggested that ACTN4 regulates the replication of HCV and IAV.^44,45^ In line with this, our findings suggested that SARS-CoV-2 infection down-regulated *ACTN4* expression, which might affect viral replication.

SARS-CoV-2 infection was reported to affect the expression of host genes by altering m6A modifications.^27^ MeRIP-seq assays using anti-m6A antibodies (Abs) were performed by using RNAs extracted from Huh7 cells with or without SARS-CoV-2 infection. As shown in Fig. 1f, the ACTN4 transcript contained m6A modifications, and the abundance of m6A signals was decreased by SARS-CoV-2 infection. Meanwhile, through meticulous analysis of the nanopore sequencing data, m6A-modified residues were detected through the *ACTN4* transcript, and the m6A ratio was also decreased following SARS-CoV-2 infection (Supplementary Fig. 2h). To validate this variation in m6A modification among *ACTN4* transcripts, methylated RNA immunoprecipitation (MeRIP) assays were performed using anti-m6A Abs in ACTN4 overexpressed or SARS-CoV-2 infected Huh7 cells, then levels of enriched RNA were measured by using reverse transcribed quantitative PCR (RT-qPCR). The abundant m6A-modified exogenous *ACTN4* transcripts were enriched by using anti-m6A Abs compared to a control IgG (Fig. 1g). In contrast, reduction of m6A modification levels in endogenous *ACTN4* transcripts upon SARS-CoV-2 infection were detected (Fig. 1h). Notably, the mRNA levels of several m6A modification related proteins, such as METTL3, WTAP, METTL14 and ALKBH5, were not affected at 24 h after SARS-CoV-2 infection (Supplementary Fig. 2d–g). Taken together, above data indicated that SARS-CoV-2 infection altered the m6A modifications in cells, which correlated with the decreased of ACTN4 expression.

### m6A modifications on *ACTN4* mRNA promotes its expression

**Fig. 2:**
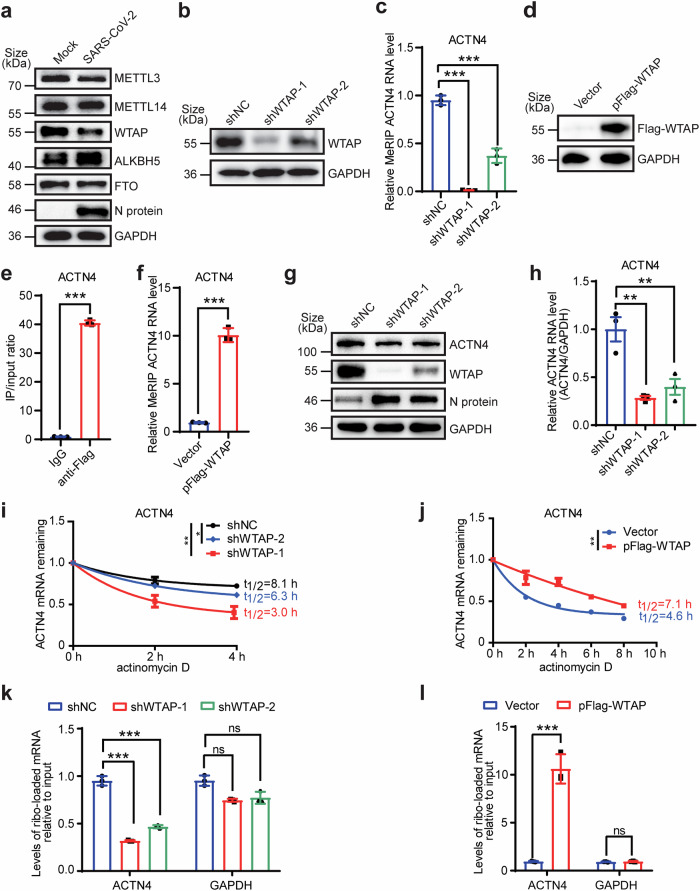
The m6A modification and its catalytic complex are essential for ACTN4 expression. **a** SARS-CoV-2 infection alters the expression of m6A-related proteins. WB assays of METTL3, METTL14, WTAP, ALKBH5, FTO and SARS-CoV-2 N protein levels in Huh7 cells infected with WT SARS-CoV-2 at MOI = 1 for 48 h. GAPDH was used as the loading control. **b**–**f** *WTAP* knockdown or overexpression affects the m6A modification level of *ACTN4* mRNA. **b**, **d** WB assays of extracts from Huh7 cells treated with pFlag-WTAP (**d**) or shRNA (**b**) WTAP expression was assessed using anti-Flag/anti-WTAP Abs, and GAPDH was served as the loading control. **c**, **e**, **f** Modulation of *ACTN4* mRNA methylation was affected by WTAP. Total RNAs were extracted from Huh7 cells with *WTAP* overexpression (**e,** **f**) or knockdown (**c**) and subjected to MeRIP-qPCR. Data are means ± SEMs (*n* = 3). ***P < 0.001, unpaired Student’s *t*-test (**e**, **f**) or one-way ANOVA (**c**). **g**, **h** *WTAP* knockdown inhibits ACTN4 expression. **g** WB assays of ACTN4, WTAP, N protein levels in Huh7 cells transfected with scramble shRNA (shNC) or two WTAP-specific shRNAs (shWTAP-1 and shWTAP-2). **h** RT-qPCR analysis of the relative *ACTN4* mRNA levels in *WTAP* knockdown Huh7 cells. Data are means ± SEMs (*n* = 3). **P ≤ 0.01, one-way ANOVA. **i**, **j** WTAP enhances *ACTN4* mRNA stability. Relative *ACTN4* mRNA levels remaining after actinomycin D (4 µg/mL) treatment in *WTAP* knockdown (**i**) or overexpressing (**j**) Huh7 cells as assessed using qRT-PCR. Data are means ± SEMs (*n* = 3). *P ≤ 0.05, **P ≤ 0.01, unpaired Student’s *t*-test (**j**) or one-way ANOVA (**i**). **k**, **l** WTAP increases the ribosome loading onto *ACTN4* transcripts. *WTAP* knockdown (**k**) or overexpressing (**l**) Huh7 cells were used to analyze input and ribosome-loaded *ACTN4* RNA levels at 48 hpi, *GAPDH* was set as the control. Data are means ± SEMs (*n* = 3). ***P < 0.001, ns: not significant, unpaired Student’s *t*-test (**l**) or one-way ANOVA (**k**)

SARS-CoV-2 infection leads to alterations in the expression levels and distribution patterns of m6A modification-related proteins in various cell types.^26,27^ After SARS-CoV-2 infection, the expression of methyltransferases WTAP and METTL3 were decreased, while the expression of demethyltransferase ALKBH5 was enhanced (Fig. 2a). Further experiments demonstrated that depletion of WTAP reduced levels of m6A modifications on *ACTN4* mRNAs (Fig. 2b, c), whereas overexpressed WTAP was capable to target *ACTN4* mRNA for binding and increased levels of m6A modified *ACTN4* transcripts (Fig. 2d–f). In contrast, knockdown of METTL3 or METTL14 only did not exert discernible effects on *ACTN4* expression (Supplementary Fig. 3a–h). However, similar with WTAP depletion, knocking down METTL3 and METTL14 in the same cells decreased both m6A modifications and mRNA levels of *ACTN4* (Supplementary Fig. 4a–c).

Recent studies have illuminated the impact of METTL3/METTL14 depletion on SARS-CoV-2 replication in different cell contexts.^27^ Consistently, depletion of METTL3, METTL14 or WTAP by indicated shRNAs enhanced the replication of SARS-CoV-2 (Supplementary Fig. 5a–i), while knockdown of ALKBH5 caused opposite effects (Supplementary Fig. 5j–l). While, depletion of WTAP led to augment the expression of the SARS-CoV-2 N protein while reducing both mRNA and protein levels of ACTN4 (Fig. 2g, h). The m6A modifications affect mRNA transportation, mRNA stability and translation efficiency, etc.^55–57^ To elucidate the mechanisms by which m6A modification regulates ACTN4 expression, the *ACTN4* mRNA decay rate and ribosome loading capacity under WTAP overexpression or depletion were examined. The decreased *ACTN4* mRNA stability was detected after WTAP depletion (Fig. 2i), whereas was increased by exogenous expressed WTAP (Fig. 2j). The *ACTN4* mRNA ribosome loading efficiency exhibited a similar trend as RNA stability (Fig. 2k, l, Supplementary Fig. 6a, b). Furthermore, exogenous WTAP was found to dose-dependently promote ACTN4 expression (Supplementary Fig. 6d), whereas no interactions were detected between these two proteins (Supplementary Fig. 6c). Additionally, without affecting levels of *ACTN4* mRNA, its expression was enhanced by depletion of ALKBH5, but was decreased by exogenous ALKBH5 (Supplementary Fig. 3i–l). While, FTO was found not to regulate ACTN4 expression (Supplementary Fig. 3m–p). To sum up, these data revealed that the m6A modification levels of *ACTN4* mRNA determine its expression by affecting mRNA stability and translation efficiency.

### ACTN4 inhibits SARS-CoV-2 replication and interacts with nsp12

**Fig. 3:**
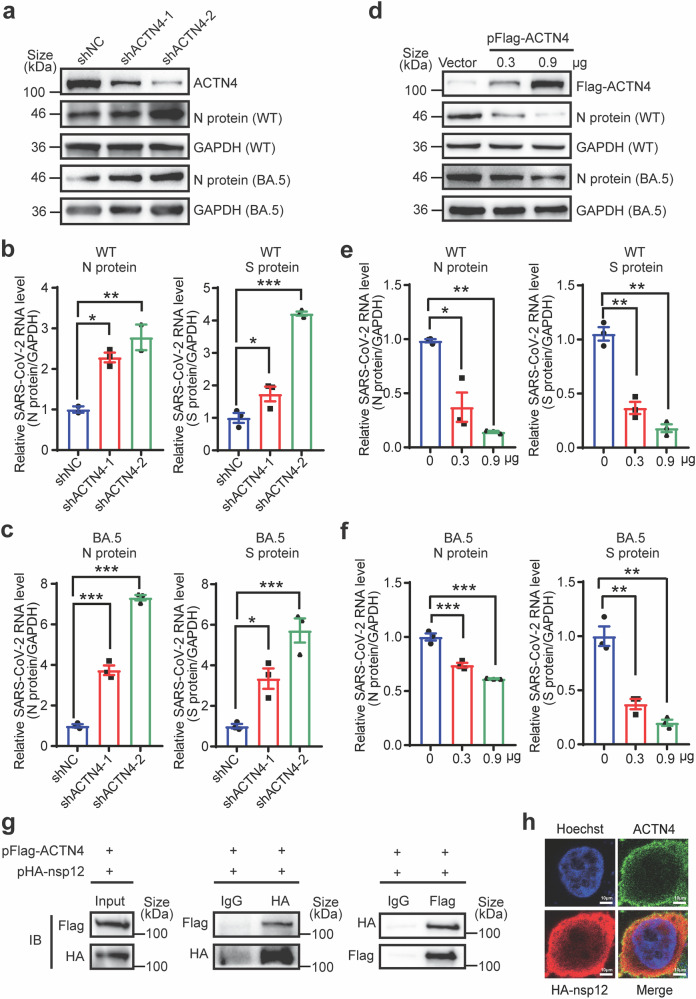
ACTN4 inhibits SARS-CoV-2 replication and interacts with nsp12. **a**, **d** WB assays of ACTN4 and viral protein N protein levels. *ACTN4* was knocked down (**a**) or overexpressed (**d**) in Huh7 cells infected with WT SARS-CoV-2 or the BA.5 mutant at MOI = 1. GAPDH was used as the loading control. **b**, **c** *ACTN4* knockdown promotes viral replication. RT-qPCR was used to determine the SARS-CoV-2 WT (**b**) or BA.5 (**c**) RNA levels in *WTAP* knockdown Huh7 cells at 48 hpi. Relative levels of SARS-CoV-2 RNA were quantified using RT-qPCR with specific primers targeting the *N* gene and *S* gene. Data are means ± SEMs (*n* = 3). *P ≤ 0.05, **P ≤ 0.01, ***P < 0.001, one-way ANOVA. **e**, **f** Overexpression of ACTN4 inhibits viral replication. RT-qPCR was used to determine SARS-CoV-2 WT (**e**) or BA.5 (**f**) RNA levels in WTAP-overexpressing Huh7 cells at 48 hpi. Relative levels of SARS-CoV-2 RNA were quantified using RT-qPCR with specific primers targeting the *N* gene and *S* gene. Data are means ± SEMs (*n* = 3). *P ≤ 0.05, **P ≤ 0.01, ***P < 0.001, one-way ANOVA. **g** ACTN4 interacts with viral RdRp nsp12. Huh7 cells were transfected with pHA-nsp12 and pFlag-ACTN4. Co-IP was performed using anti-HA/anti-Flag Abs or IgG. The immuno-blots were probed with the anti-Flag or anti-HA Abs. Left panels were input, middle panels were incubated with anti-HA Abs then detected with anti-Flag/anti-HA Abs, right panels were incubated with anti-Flag Abs then detected with anti-HA/anti-Flag Abs. **h** Confocal microscopy images of Huh7 cells transfected with empty vector or pHA-nsp12. Co-staining was performed using an anti-ACTN4 Abs (green) and anti-HA Abs (red), together with Hoechst to stain the nucleus (blue). The scale on the picture indicates 10 μm

ACTN4 has been documented to regulate the replication of several viruses,^44,45^ and our findings showed that ACTN4 was specifically downregulated following SARS-CoV-2 infection (Fig. 1, Supplementary Fig. 2). To elucidate the precise roles of ACTN4 in viral replication, ACTN4 was overexpressed or knocked down in Huh7 cells, and levels of SARS-CoV-2 replication were examined. As depicted in Fig. 3a, depletion of ACTN4 increased the expression of N proteins encoded by wild-type (WT) SARS-CoV-2 or Omicron BA.5 variant. After measuring RNA levels of both N and S genes, the viral RNA levels of both WT SARS-CoV-2 and the Omicron BA.5 variant were increased by ACTN4 depletion (Fig. 3b, c). Conversely, overexpressing ACTN4 inhibited viral protein expression and decreased viral replication of both WT SARS-CoV-2 and the Omicron BA.5 variant (Fig. 3d–f). ACTN4 was reported to promote HCV and IAV replication by targeting their RdRp, NS5B and NP.^44^ To verify whether ACTN4 was able to target SARS-CoV-2 RdRp for interactions, co-immunoprecipitation (IP) assays were performed by co-expressing both ACTN4 and the key RdRp component nsp12. Similarly, the interactions between ACTN4 and nsp12 were detected (Fig. 3g). Moreover, the indirect fluorescent assay showed that exogenous ACTN4 co-localized with nsp12 in the cytoplasm (Fig. 3h). Thus, ACTN4 was verified to impede SARS-CoV-2 replication and interact with the nsp12.

### ACTN4 competes with nsp7/nsp8 to bind nsp12

**Fig. 4:**
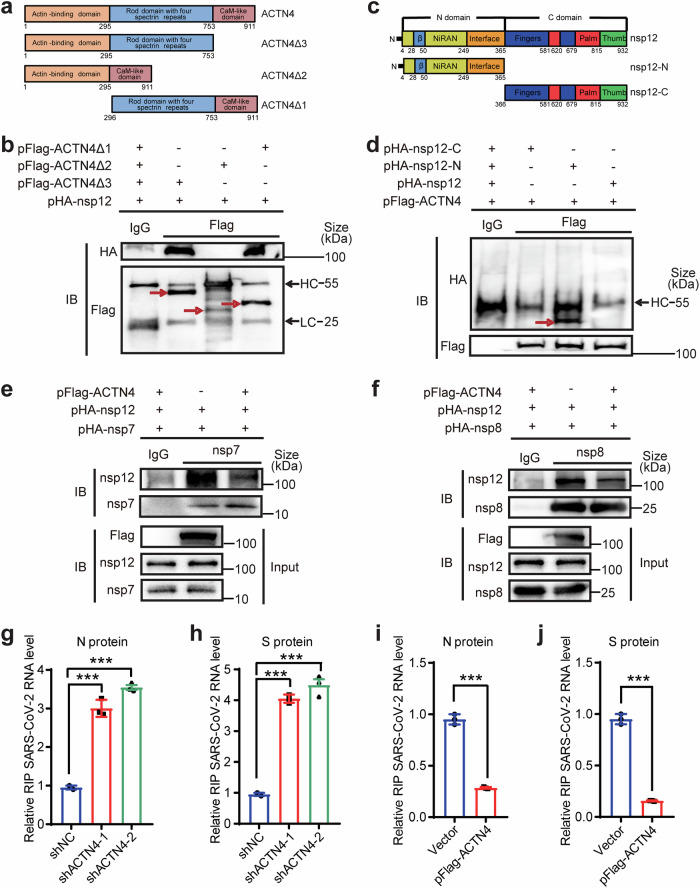
ACTN4 specifically competes with SARS-CoV-2 RNA to bind nsp12. **a**, **c** Schematic representation of ACTN4 and nsp12 proteins. ACTN4 contains three domains: 1-295 amino acids (aa), 296-753 aa, and 754-911 aa. The N-terminal domain of nsp12 consists of 1–365 aa and the C-terminal domain consists of 366–932 aa. **b**, **d** nsp-12-interacting domain of ACTN4. ACTN4△1, ACTN4△2, or ACTN4△3 was co-transfected with nsp12 (**b**) or pHA-nsp12-N or pHA-nsp12-C was co-transfected with ACTN4 (**d**) into Huh7 cells. Co-IP was performed using anti-Flag Abs. IgG Abs were set as the control. Reactive proteins were visualized using the indicated Abs. ‘HC’ represents heavy chain; ‘LC’ represents light chain. **e**, **f** ACTN inhibits nsp7/nsp8 binding with nsp12. Co-IP samples obtained from HEK293T cells transfected with pFlag-ACTN4, pHA-nsp12, or pHA-nsp7/pHA-nsp8 were measured using anti-nsp7 or anti-nsp8 Abs. The input samples were measured using the indicated Abs. **g**–**j** Formaldehyde crosslinked RIP-qPCR. The *ACTN4* knockdown or overexpressed Huh7 cells infected with WT SARS-CoV-2 at MOI = 1 for 48 h were formaldehyde-crosslinked and incubated with IgG or anti-nsp12 Abs. The abundance of SARS-CoV-2 RNA was detected using RT-qPCR using specific primers targeting the *N* (**g**, **i**) and *S* (**h**, **j**) genes. Fold enrichment was determined by dividing the fold change between the IP and the input, and the mean value of the vector or shNC were defined as 1. Data are means ± SEMs (*n* = 3). ***P ≤ 0.001, unpaired Student’s *t*-test (**i**, **j**) or one-way ANOVA (**g**, **h**)

ACTN4 targets various viral proteins that participate in the replication processes of HCV and IAV.^44,45^ To investigate the potential mechanisms by which ACTN4 targets nsp12 to impede viral replication, truncated mutants of both proteins were constructed to delineate the precise domains responsible for their interaction. Subsequent co-IP experiments demonstrated that the Rod domain of ACTN4 (amino acids 296-753) was instrumental in its interaction with nsp12 (Fig. 4a, b, Supplementary Fig. 7a). Additionally, the N-terminal motif of nsp12, consisting of NiRAN and Interface domains, was targeted by ACTN4 for their interactions (Fig. 4c, d, Supplementary Fig. 7b).

Notably, nsp7 and nsp8 are two other components of the RdRp complex, which directly interact with nsp12. The N-terminal motif of nsp12, targeted by ACTN4, is essential for the formation of these hetero-trimer complexes. However, neither nsp7 nor nsp8 interacted with ACTN4 (Supplementary Fig. 7c, d). Notably, exogenous ACTN4 largely impaired the interactions between nsp7 and nsp12 and between nsp8 and nsp12 (Fig. 4e, f). Given that ACTN4 targets nsp12 for binding, it is plausible that ACTN4 inhibits SARS-CoV-2 replication by interfering with the formation of RdRp complexes and the subsequent RNA loading capacity of nsp12. RIP assays using anti-nsp12 antibodies in WT SARS-CoV-2 infected Huh7 cells revealed that the levels of viral RNAs pulled down by nsp12 were substantially increased upon ACTN4 depletion (Fig. 4g, h). Conversely, ACTN4 overexpression substantially inhibited the binding of nsp12 to viral RNAs (Fig. 4i, j). Collectively, these findings suggested that ACTN4 competes with nsp7/nsp8 to interact with nsp12, consequently impairing SARS-CoV-2 replication by inhibiting the viral RNA binding capacity of nsp12.

### ACTN4 inhibitors and agonists affect SARS-CoV-2 replication in cells

**Fig. 5:**
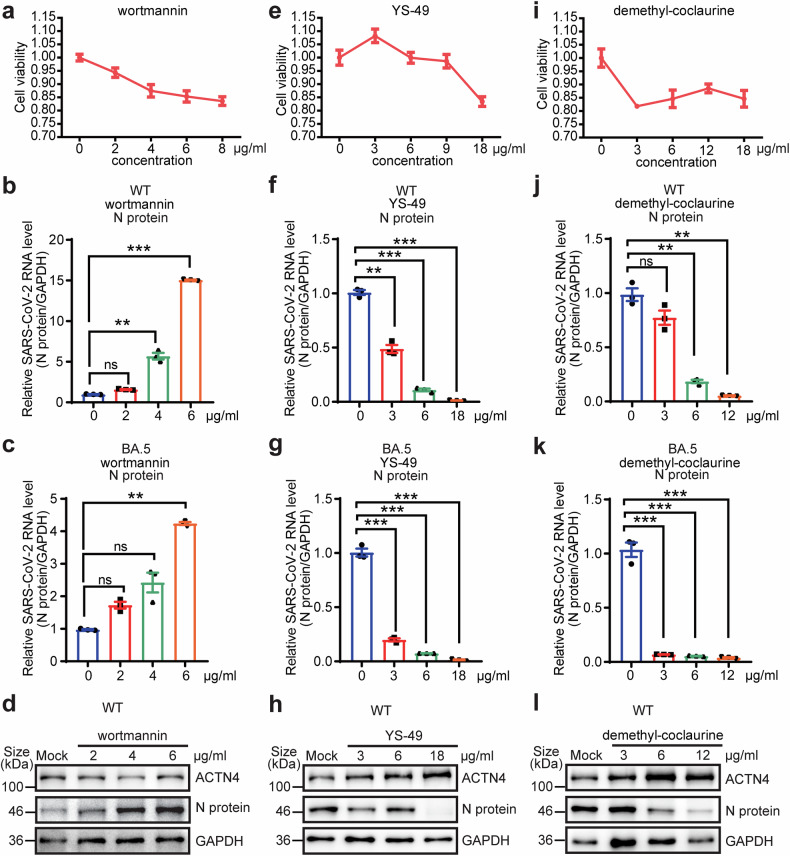
ACTN4 inhibitors and agonists affect SARS-CoV-2 replication in cells. **a**, **e**, **i** Huh7 cells were seeded into 96-well plates and treated with increasing amounts of each indicated compound for 48 h. Cell viability was assessed using the CCK-8 assay. Error bars denote mean ± sd of 3 independent replicates. Huh7 cells were infected with WT SARS-CoV-2 or the BA.5 mutant at MOI = 1 for 48 h, after the cells were treated with indicated compounds for 24 h. RT-qPCR was used to detect relative SARS-CoV-2 WT (**b**, **f**, **j**) or BA.5 (**c**, **g**, **k**) RNA levels affected by wortmannin (**b**, **c**), YS-49 (**f**, **g**) and demethyl-coclaurine (**j**, **k**), using specific primers targeting the *N* gene. Data are means ± SEMs (*n* = 3). **P ≤ 0.01, ***P < 0.001, ns: not significant, one-way ANOVA. **d**, **h**, **l** WB assays of ACTN4 and viral N proteins expression in above SARS-CoV-2 WT-infected Huh7 cells. ‘Mock’ represents 0 µg/mL and equal amount of DMSO. GAPDH was used as the loading control

The wortmannin, defined as the inhibitor of both PI3K and ACTN4, has been characterized to counteract the ACTN4-mediated promotion of IAV replication.^45^ In line with this, with less than 20% reduction of cell viability (Fig. 5a), wortmannin treatment led to reduce ACTN4 transcriptional and expression levels (Supplementary Fig. 8a, b), enhanced the replication of both WT SARS-CoV-2 and the Omicron BA.5 variant in a dose-dependent manner (Fig. 5b–d, Supplementary Fig. 10a).

ACTN4 has been associated with various cellular processes, such as cell proliferation, apoptosis, and carcinogenesis.^47,52,58^ In contrast to the inhibitory effects to the PI3K by wortmannin, potential compounds capable to activate PI3K might possess the ability to inhibit the viral replication of SARS-CoV-2 through affecting ACTN4. Then, we picked out several small molecules that have the potential to act as agonists to promote ACTN4 expression, such as 740 Y-P, poria cocos extract, YS-49, and demethyl-coclaurine. Following screening of their effects on SARS-CoV-2 replication, two potential ACTN4 agonists, YS-49 and demethyl-coclaurine, which largely inhibited the replication of wild-type SARS-CoV-2 and the Omicron BA.5 variant in a dose-dependent manner, with less than 20% cytotoxic effects (Fig. 5e–l, Supplementary Fig. 10b, c, Supplementary Fig. 11). Furthermore, both YS-49 and demethyl-coclaurine increased ACTN4 expression by promoting its transcriptional levels in a dose-dependent manner (Supplementary Fig. 8c–f). In contrast, other two PI3K agonists 740 Y-P and poria cocos extract neither affected the viral replication nor the ACTN4 expression (Supplementary Fig. 9a–h). Thus, the ACTN4 inhibitor wortmannin enhanced SARS-CoV-2 replication, its agonists YS-49 and demethyl-coclaurine inhibited SARS-CoV-2 replication in vitro. Nevertheless, present findings not only confirmed the negative regulation of SARS-CoV-2 replication by ACTN4, but also suggested its potential as an antiviral target against SARS-CoV-2.

### ACTN4 agonists inhibit SARS-CoV-2 infection in mice

**Fig. 6:**
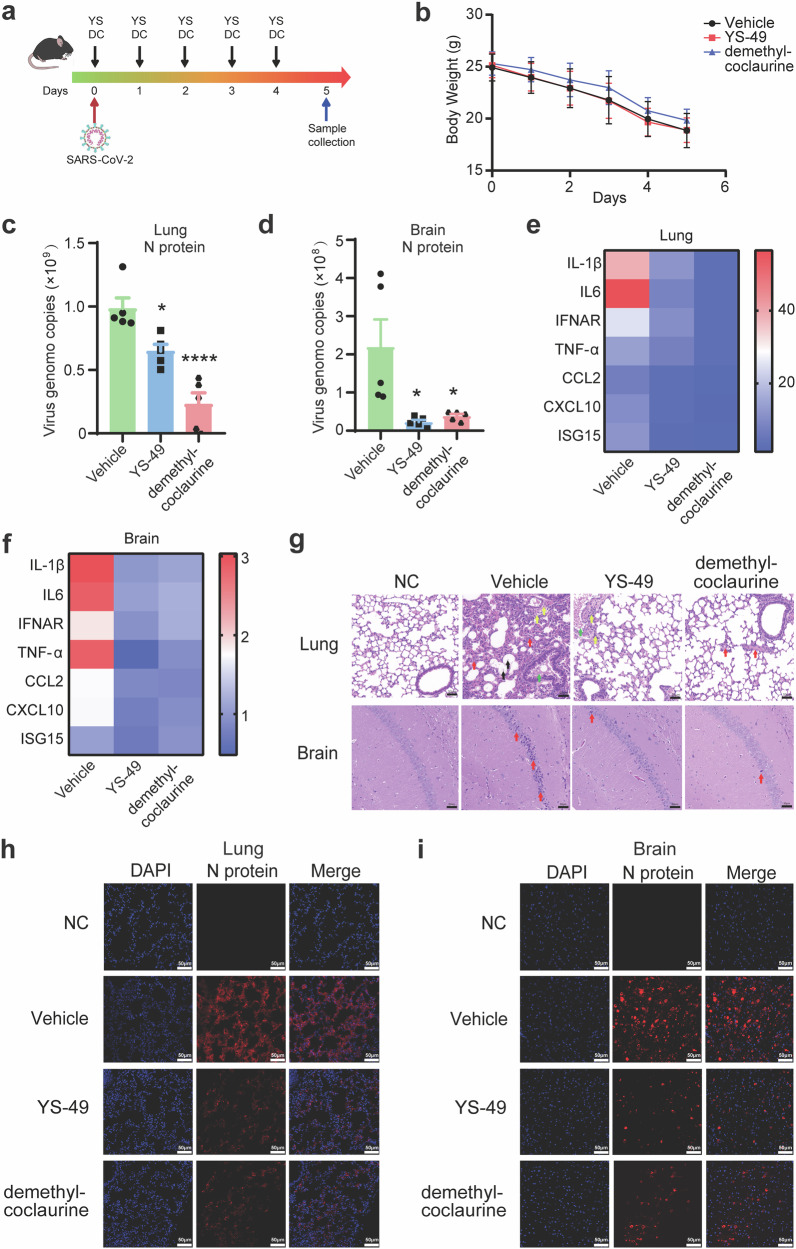
ACTN4 agonists inhibit SARS-CoV-2 infection in mice. **a** Experimental timeline for SARS-CoV-2 infection in K18-hACE2 mice. K18-hACE2 mice were intranasally inoculated with 1 × 10^5^ PFU SARS-CoV-2 BA.5 and treated with DMSO (Vehicle, IG, five times, *n* = 5), YS represented YS-49 (50 mg/kg, IG, five times, *n* = 5), DC represented demethyl-coclaurine (50 mg/kg, IG, five times, *n* = 5). **b** Body weights of mice in the presence of 50 mg/kg YS-49 or demethyl-coclaurine or indicate amount of DMSO were recorded daily, the data were presented as the line chart. **c**, **d** Viral loads in lung (**c**) and brain (**d**) tissues at 5 dpi were quantified using RT-qPCR. Data are means ± SEMs (*n* = 5). *P ≤ 0.05, ****P < 0.0001, one-way ANOVA. **e**, **f** Relative levels of indicated inflammatory genes, *IL-1β, IL-6, IFNAR, TNF-α, CCL2, CXCL10*, and *ISG15*, were measured in the lung and brain using RT-qPCR, at day 5. Different colors represented the abundance changes of each genes, and blue to red indicating a gradual increase in abundance. **g** Hematoxylin and eosin staining of lung and brain tissues. The differences of histology were marked with arrows. NC represents normal K18-hACE2 mice without virus infection. Scale bars, 50 μm. **h**, **i** Detection of SARS-CoV-2 N proteins in lung (**h**) and brain (**i**) tissues were via immunofluorescence staining. Scale bars, 50 µm

In addition to the above assays performed in cells, we investigated the potential inhibitory effects of the two agonists on SARS-CoV-2 infection using K18-hACE2 transgenic mice. The drug administration (intragastric, IG) and the inoculation of 1 × 10^5^ pfu Omicron BA.5 viruses were performed at day 0. Subsequently, mice were then administered with YS-49 or demethyl-coclaurine once a day until day 5 (Fig. 6a). Based on preliminary experiments to determine the optimal drug dosage in mice (Supplementary Fig. 12a, b), a concentration of 50 mg/kg was selected for subsequent experiments. Mice subjected to the indicated treatments were infected with the Omicron BA.5 variant. The mice were sacrificed on day 5, and viral loads in lung and brain tissues were assessed. As presented in Fig. 6b, the body weighs of mice treated with or without the agonists did not show differences. Other clinical outcomes, such as the autonomic activity status, the state of nervous response, the hair state, the hunchback posture and the breathing status, were also monitored daily, and no significant differences were observed (Supplementary Table 1). While, both agonists reduced both viral RNA copies and viral titer in lung (~1-5 fold) and brain (~5-10 fold) tissues (Fig. 6c, d, Supplementary Fig. 12c, d), compared to the vehicle. In addition, cytokine gene expression was measured in both lungs and brains at day 5. The relative abundance of *IL-1β*, *IL-6*, *IFNAR*, *TNF-α*, *CCL2*, *CXCL10*, and *ISG15* mRNAs was separately measured. Compared to vehicle, mice with YS-49 or demethyl-coclaurine resulted in the reduction of these cytokines (Fig. 6e, f). Hematoxylin and eosin staining revealed reducing lung and brain lesions in YS-49 or demethyl-coclaurine-treated mice, resulting in decreased alveolar wall proliferation and reduced neuronal damage (Fig. 6g). Tissue immune-staining indicated reduced fluorescence intensity of SARS-CoV-2 N protein (cy3) in lung and brain tissues (Fig. 6h, i, Supplementary Fig. 12e, f). In conclusion, the ACTN4 agonists demethyl-coclaurine and YS-49 were implicated to hold potential as antiviral drugs against SARS-CoV-2.

## Discussion

**Fig. 7:**
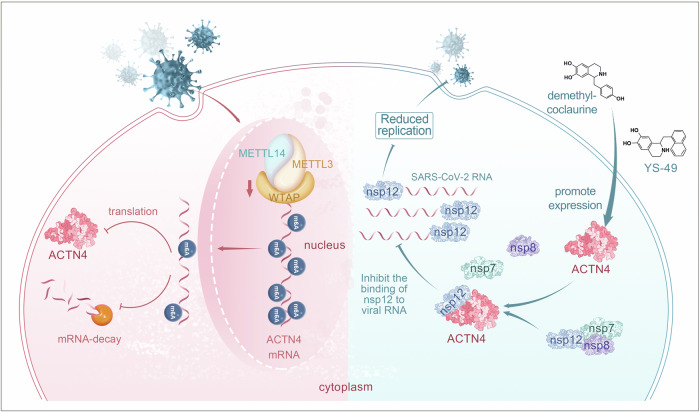
The model of intricate interplay between host factor ACTN4 and SARS-CoV-2. (Left half part) SARS-CoV-2 infection decreases the m6A related factors and global m6A modification levels in cells, which correlates with reduction of ACTN4 expression. (Right half part) ACTN4 targets viral protein nsp12 for binding to block the viral RdRp complex formation, which causes inhibition of SARS-CoV-2 replication. Two ACTN4 agonists, YS-49 and demethyl-coclaurine, are identified to inhibit viral replication. This graphic model was created using the software of Photoshop

The present study comprehensively elucidated the intricate interplay between the host factor ACTN4 and SARS-CoV-2, a model of which is shown in Fig. 7. Comparative analysis of host gene expression before and after SARS-CoV-2 infection revealed the downregulation of ACTN4 expression. The reduction of *ACTN4* mRNA m6A modification levels resulted in decreased mRNA stability and translational efficiency, ultimately leading to the inhibition of ACTN4 expression. Furthermore, ACTN4 acts as a competitor of SARS-CoV-2 RNA and the RdRp complex in binding with nsp12, thereby inhibiting viral replication. Notably, our screening identified two promising ACTN4 agonists that effectively suppressed SARS-CoV-2 infection both in vitro and in vivo. Thus, our study unveiled a novel communication pathway between SARS-CoV-2 and host cells that is orchestrated via a feedback loop encompassing RNA metabolism and ACTN4.

Studies on the involvement of ACTN4 in virus infection are limited to HCV, IAV, and rotavirus infections, while the precise molecular mechanisms underlying the role of ACTN4 during viral infection remain enigmatic.^44–46^ This study revealed that *ACTN4* knockdown enhanced the replication of various SARS-CoV-2 variants, vice versa. Similar to its interactions with HCV and IAV, ACTN4 interacts and co-localizes with the SARS-CoV-2 RdRp, nsp12. In contrast to enhanced viral replication of HCV and IAV by ACTN4, it potentially competes with nsp12 for binding to SARS-CoV-2 RNA, resulting in reduced nsp12 association with viral RNA and consequently, the inhibition of viral replication. Moreover, the N-terminus of nsp12 involved with nsp7/nsp8 binding, was characterized as the ACTN4 interaction motif, leading to the hypothesis that ACTN4 competed with nsp7/8 to bind nsp12. Consistently, following co-IP assays shown ACTN4 prevented nsp7/nsp8 from binding nsp12 and suppressing the association of nsp12 with SARS-CoV-2 RNA, leading to the inhibition of virus replication. Preliminary experiments corroborated these findings. Thus, ACTN4 behaved as a novel host factor capable of inhibiting SARS-CoV-2 infection, offering a new avenue for future research and therapeutic exploration. However, further investigations are warranted to elucidate the precise binding sites of this competitive interaction and whether this competition persists following the occurrence of mutations within these binding sites.

SARS-CoV-2 causes dramatic changes of m6A modification by affecting m6A related machineries in different cells.^26,27,40^ Consistent with previous studies, SARS-CoV-2 infection in Huh7 cells led to changes of m6A related enzymes expression, but without affecting their mRNA levels. This implied that m6A related enzymes were regulated via the post-translational pathway by viral infection, which needed further investigations. SARS-CoV-2 maintained very high levels of replication in cells, therefore, there should be some potential mechanisms to overcome the obstacle of viral replication caused by the anti-viral cytokine, ACTN4. Similarly, we also found SARS-CoV-2 infection in Huh7 cells led to the reduction of whole m6A modification. Detailed analysis of both nanopore sequencing and MeRIP-seq using anti-m6A Abs revealed that m6A levels of ACTN4 mRNA were decreased after viral infection, and similar pattern of the m6A motifs (GGACH) (data not shown) was also obtained. The overlapped m6A modified regions were mainly located within its 3’ end of ACTN4, even the variety of m6A distribution was detected by these two high-through-put sequencing assays. The m6A modifications were revealed to be essential for translational efficiency and RNA stability, which determined the levels of ACTN4 proteins. While, the exact functional m6A modified sites of ACTN4 mRNA will be worthy to be further identified. These findings underscore the notion that SARS-CoV-2 infection can modulate host pathways by altering m6A modifications on host mRNAs. Nevertheless, the precise underlying molecular mechanisms remain elusive, necessitating further investigations to unravel the intricate relationships among the virus, host, and m6A system and how viral infection affects the m6A system and host functions.

As the defined inhibitor of ACTN4, wortmannin was reported to cause the translocation of ACTN4 from cytoplasm to nucleus.^45,59^ We found that wortmannin was capable to decrease the transcription of ACTN4 and enhance SARS-CoV-2 replication in cells. On the contrary, compared to other two PI3K agonists 740 Y-P and poria cocos extract, the agonists YS-49 and demethyl-coclaurine were characterized to effectively inhibit the replication of wild-type SARS-CoV-2 and BA.5 in Huh7 cells, which correlated with increase of ACTN4 expression, by combining both in vitro and in vivo experimental data. ACTN4 was also found to be ubiquitinated and degraded via the ubiquitination mediated proteasomal pathway.^60^ These new findings implied that the expression of ACTN4 was closely related with PI3K pathway, but further detailed studies were still needed to elucidate the exact mechanisms.

To date, those commonly used anti-SARS-CoV-2 drugs, including Paxlovid, VV116, and Azvudine,^4,6,7,61^ which targeted different viral proteins, would cause selection pressure to viruses and lead to the emergence of resistant mutant viruses. However, these two agonists were able to broadly block the viral infection by targeting the host cytokine, without causing further mutations. Considering the administration of these two agonists, they both held the potential to prevent the SARS-CoV-2 infection and alleviate the COVID-19 symptoms in mice. Notably, demethyl-coclaurine exhibited lower toxicity and superior antiviral efficacy compared to YS-49. Demethyl-coclaurine, also named higenamine, is a constituent of the Chinese herbal medicine aconite root, which exerts therapeutic actions, such as fever reduction, inflammation alleviation, and pain relief.^62,63^ These attributes align with the symptoms experienced by patients with coronavirus disease, making demethyl-coclaurine a promising candidate for clinical treatment. Thus, ACTN4 is a potential target for the development of SARS-CoV-2 therapeutics and clinical management of SARS-CoV-2 infections.

In summary, our study provided compelling evidence that m6A modification plays a pivotal role in mediating the interaction between SARS-CoV-2 and the host protein, ACTN4. Particularly, ACTN4 binds to the viral RdRp, nsp12, leading to reduced nsp12 binding to SARS-CoV-2 RNA and subsequent inhibition of viral replication. Moreover, we identified a novel potential antiviral agent and drug for combating SARS-CoV-2 infection, as demonstrated through in vitro and in vivo experiments. Our findings contribute to a better understanding of the involvement of SARS-CoV-2-induced m6A modifications in virus–host interactions, paving the way for the development of prophylactic and therapeutic strategies against SARS-CoV-2-associated diseases. Future research endeavors should aim to delve deeper into the following aspects: first, the intricate regulatory mechanisms governing the feedback loop require further elucidation to gain comprehensive insights into the role of ACTN4 in SARS-CoV-2 replication; second, comprehensive safety assessments of the two agonists in mice are warranted, alongside further investigations to determine whether they effectively inhibit viral infection within the established safety parameters; third, extensive exploration are needed to elucidated other alternative mechanisms how these two agonists inhibits SARS-Cov-2 infection; fourth, optimization of the two agonists through artificial intelligence and structural analyses to reduce toxicity and enhance antiviral efficiency should be pursued.

## Materials And Methods

### Facility and ethics statements

All experiments involving live SARS-CoV-2 were conducted in a biosafety level 3 facility maintained under negative pressure at the Wuhan Institute of Virology (WIV), Chinese Academy of Sciences (CAS) (approval No. NBL3-202320). All animal experiments were carried out in strict accordance with the institutional guidelines and approved by the Institutional Animal Care and Use Committee of WIV, CAS (approval No. WIVA42202206).

### Cell culture and cell manipulation

A549-ACE2 cells (generated in house), Huh7 cells (Resource Center of WIV, CAS), Vero E6 cells (CRL-1586; ATCC) and HEK293T human embryonic kidney cells (CRL-11268; ATCC) were cultured in Dulbecco’s modified Eagle’s medium (DMEM) (C11995500BT, Thermo Fisher Scientific, Wilmington, DE, USA) containing 10% fetal bovine serum (Gibco) at 37 °C in the presence of 5% CO_2_. Cells were transfected with plasmids using lipofectamine 2000 reagent (cat. no. 11668-019; Invitrogen, Carlsbad, CA, USA) following the instructions suggested by the manufacturer. For experiments involving cell treatments, Huh7 cells were first treated with YS-49 (E0785), demethyl-coclaurine (S3294) (both from Selleck) or wortmannin (S1952; Beyotime Biotechnology) for 24 h, and then infected with SARS-CoV-2 and maintained for another 72 h.

### Virus strains and Infection

SARS-CoV-2 (IVCAS 6.7512) and Omicron BA.5 (IVCAS 6.8981) were obtained from the Virus Resource Center of WIV, CAS. The viruses were propagated in Vero E6 cells and were titrated by plaque assay according to a standard procedure. In brief, Vero E6 cells were inoculated into a 24-well plate at 1 × 10^5^ cells/well. After 24 h, the cells were infected a 10-fold diluted viral stock solution in 200 µL of DMEM at 37 °C for 1 h. After removal of the inoculant, the cells were cultured in DMEM containing 0.9% methylcellulose at 37 °C for 4 days. Plaques were monitored and counted. Different susceptibility of cell lines to SARS-CoV-2, therefore, we tested different viral multiplication of infection (MOI) gradients and ultimately determined the optimal MOI for each cell line. Finally, we used MOI = 0.05 in Vero-E6 cells and MOI = 1 in A549-ACE2 and Huh7 cells for the following experiments.

### Nanopore direct RNA sequencing and data analysis

Total RNA was extracted from cells infected with SARS-CoV-2 at 48 h utilizing TRIzol Reagent (Invitrogen, Cat. No. 15596026), then purified using an Oligo(dT) kit (Thermo Fisher Scientific). Two micrograms of the purified RNA were employed for library preparation, adhering to the Oxford Nanopore direct RNA sequencing protocol (SQK-RNA002). The prepared libraries were loaded onto a FLO-MIN106D flow cell and sequenced using a MinION device (Oxford Nanopore Technologies). Data analysis was conducted at BENAGEN, setting a Q-value of 7 as the threshold for read screening. Guppy software version 3.4.5 (Oxford Nanopore Technologies) was utilized for base-calling. The multi_to_single_fast5 tool from the ont_fast5_api (version 3.1.6, available at https://github.com/nanoporetech/ont_fast5_api) was used to convert multi-FAST5 files to single-read files. The reads from Oxford Nanopore Technologies were aligned to the Homo sapiens cDNA reference genome (Ensembl release-102) using Minimap2 (version 2.24) with the options -ax splice -uf -N 32 -k 14 -t 20. Samtools (version 1.12) was then used to generate files in BAM format. Transcript abundance from the ONT reads was quantified using Salmon (version 1.4.0), based on the BAM files produced by Minimap2. Differential gene expression analysis was performed using edgeR, and the results were visualized with ggplot2 (version 3.4.2). The false discovery rate (FDR) cutoff for identifying differentially expressed genes was set at 0.05, and the absolute log fold change (logFC) threshold was set to greater than 2. Enrichment analysis of these differentially expressed genes was conducted using the R package clusterProfiler (version 4.0.5), with the parameters for GO enrichment analysis set to pAdjustMethod = “fdr”, pvalueCutoff = 0.05, and qvalueCutoff = 0.2. Identification of m6A sites was performed based on the de novo model of Tombo, followed by the MINES process for assigning m6A modulation status. Visualization of methylation sites was achieved using R (version 4.0.5) and ggplot2.

### MeRIP-seq and data analysis

Briefly, total RNAs were extracted from Huh7 cells with/without SARS-CoV-2 infection. After the RNA integrity and concentration were confirmed, 50 μg RNAs were enriched by VAHTS mRNA Capture Beads (VAHTS, cat. NO. N401-01/02). After treating with 20 mM ZnCl_2_ following 95 °C for 10 min, 10% RNA fragments was saved as “Input”, while the rest was proceeded to m6A immunoprecipitation (IP) using anti-m6A Abs (Synaptic Systems, 202203). The stranded RNA sequencing library was constructed using the KC-Digital^TM^ Stranded mRNA Library Prep Kit for Illumina® (Catalog NO. DR08502, Wuhan Seqhealth Co., Ltd. China) according to manufacturer’s instructions, following with enrichment, quantification and finally sequencing on DNBSEQ-T7 sequencer (MGI Tech Co., Ltd. China) with PE150 model. Raw sequencing data were processed and analyzed as previously described, The m6A site analysis was performed using de-duplicated consensus sequences, which were mapped to the reference genome (https://www.ncbi.nlm.nih.gov/gene/81) using STAR software (version 2.5.3a) with default parameters.^64^

Variation of m6A modifications on *ACTN4* mRNA was presented after normalizing the m6A coverage from IP with the m6A coverage from Input.

### Plasmids

The plasmids pFlag-WTAP, pFlag-ACTN4, pHA-WTAP, pHA-nsp12, pHA-nsp7, and pHA-nsp8 were constructed by inserting the coding sequences of the indicated genes into the pXJ40-Flag and pXJ40-HA vectors. The truncated mutants pFlag-ACTN4Δ1, pFlag-ACTN4Δ2, pFlag-ACTN4Δ3, pHA-nsp12-N, and pHA-nsp12-C were constructed using the same vectors. The sequences of the short hairpin (sh)RNAs targeting specific genes used in this study were as follows: shWTAP-1: 5′- GTTATGGCAAGAGATGAGTTA-3′, shWTAP-2: 5′- ATGGCAAGAGATGAGTTAATT-3′, shACTN4-1: 5′-GCCACACTATCGGACATCAAA-3′, shACTN4-2: 5′- CATCGCTTCCTTCAAGGTCTT-3′, shACTN4-3: 5′- CCTCTCTTTCTCAGTCTTGTA-3’. The shRNAs were individually cloned into the pLKO.1-TRC vector (Addgene, 10878) and packaged into lentiviruses as described previously.^24^ Stable knockdown cell lines were selected with 5 mg/mL puromycin.

### Western blotting and Reverse transcription quantitative PCR (RT-qPCR)

Western blotting analyses were performed as described previously.^24^ Briefly, cells were lysed by lysis buffer, centrifuged at 13,000 × *g* at 4 °C for 10 min, and the supernatant was removed. The proteins were separated using 10% sodium dodecyl sulfate-polyacrylamide gel, then transferred onto nitrocellulose membrane. The following antibodies (Abs) were used: anti-GAPDH (Cat. No. PA1-987), anti-METTL3 (15073-1-AP), anti-METTL14 (MA5-24706), anti-WTAP (PA5-52704), anti-ALKBH5 (16837-1-AP), anti-ACTN4 (19096-1-AP) purchased from Proteintech, anti-Flag (F1804, Sigma-Aldrich), anti-HA (#5017S, Cell Signaling Technology), and anti-SARS-CoV-2 nsp12 (A20233), anti-SARS-CoV-2 nsp7 (A20201), anti-SARS-CoV-2 nsp8 (A20202l), and anti-SARS-CoV-2 N protein (A20021) obtained from ABclonal. Luminescent signals were measured using the ChemiDoc MP imaging system (Bio-Rad, Hercules, CA, USA).

Total RNAs were isolated from cells using TRIzol Reagent (Invitrogen) and reverse transcribed using a HiScript First-Strand cDNA Synthesis Kit (Vazyme Biotech, Nanjing, China) according to manufacturer’s instructions. qPCR was run using SYBR Green (Yeasen Biotech, Shanghai, China) on a CFX Connect real-time system (Bio-Rad Laboratories). The primers used for qPCR were as follows: ACTN4 forward: 5′-GCAGCATGGGCGACTACAT-3′, reverse: 5′-TTGAGCCCGTCTCGGAAGT-3′, SARS-CoV-2 nucleoprotein (N) protein forward: 5′-GGGGAACTTCTCCTGCTAGAAT-3′, reverse: 5′-CAGACATTTTGCTCTCAAGCTG-3′, SARS-CoV-2 spike (S) glycoprotein forward: 5′-ACTTACTCCTACTTGGCGTGTT-3′, reverse: 5′-CTAGCGCATATACCTGCACCAA-3′, and GAPDH forward: 5′-GAAGGTGAAGGTCGGAGTC-3′, reverse: 5′-GAAGATGGTGATGGGATTTC-3′.

### Indirect immunofluorescence assay

Huh7 cells were infected with SARS-CoV-2 and harvested at 48 hpi. Cells were fixed overnight in 4% paraformaldehyde at 4 °C, washed three times with phosphate-buffered saline (PBS), incubated with 0.5% Triton X-100 at room temperature for 10 min, and blocked in 3% bovine serum albumin (BSA) at room temperature for 1 h. The cells were probed with indicated primary antibodies (1:100) at 4 °C overnight, then washed three times with PBS, and then incubated with indicated secondary antibodies at room temperature for 1 h. Nuclei were stained with Hoechst 33258 (1:1000). The cells were observed under a PerkinElmer VoX confocal microscope.

### Formaldehyde-crosslinked RIP assay

Huh7 cells were cross-linked with 1% methanol-free formaldehyde at 37 °C for 10 min, then quenched with 0.125 M glycine. After three washes with ice-cold PBS, the cells were collected and resuspended in RIP buffer (150 mM KCl, 25 Mm Tris-HCl pH 7.4, 5 mM EDTA, 0.5 mM dithiothreitol (DTT), 0.5% NP40, 100 U/mL RNase inhibitor, 100 µM phenylmethylsulfonyl fluoride, and 1 µg/mL proteinase inhibitors. The cell lysates were centrifugated at 13,000 × *g* at 4 °C for 10 min, and the supernatants were incubated with anti-Flag Abs or mouse IgG (control) at 4 °C overnight. After incubating the mixture with the protein-G agarose beads at 4 °C for 2 h, washing the beads three times with wash buffer (300 mM KCl, 25 mM Tris-HCl pH 7.4, 5 mM EDTA, 0.5 mM DTT, 0.5% NP40, 100 U/mL RNase inhibitor, 100 µM phenylmethylsulfonyl fluoride, and 1 µg/mL proteinase inhibitors), followed by washing the beads three times with RIP buffer. After proteinase K digestion, RNA was extracted using TRIzol and used in the following RT-qPCR assay.

### Methylated RIP qPCR (MeRIP-qPCR)

Briefly, total RNA was incubated with anti-m6A Abs (Synaptic Systems, Goettingen, Germany) or IgG in RIP buffer (150 mM NaCl, 0.1% NP-40, 10 mM Tris-HCl, pH 7.4) at 4 °C for 2 h. The mixture was incubated with anti-rabbit Ab-bound magnetic beads (S1432S; NEB, Ipswich, MA, USA) at 4 °C for 2 h. Then, washed the beads six times with RIP buffer and incubated with elution buffer (5 mM Tris-HCl, pH 7.5, 1 mM EDTA, pH 8.0, 0.05% SDS, and 4.2 µl of 20 mg/mL proteinase K) at 50 °C for 1.5 h. The eluted RNAs were purified using phenol/chloroform, precipitated with 75% ethanol, and subjected to RT-qPCR.

### Measurement of mRNA stability

Huh7 cells with *WTAP* knockdown or overexpression were treated with actinomycin D (4 μg/mL) for the indicated times. RNA was extracted and analyzed by RT-qPCR. RNA turnover rates and half-lives were measured and compared as previously reported.^65^ The half-lives of remaining RNAs were obtained by linear regression fitting of relative RNA remaining (ln) as a function of time using the following equation: t1/2 = ln (2)/slope.

### Ribosome loading assay

Ribosome loading onto *ACTN4* transcripts was quantified as previously described.^66^ Briefly, Huh7 cells with *WTAP* overexpression or knockdown were treated with 100 mg/mL cycloheximide at 37 °C for 10 min, washed three times with PBS, and lysed with ribosome lysis buffer (10 mM Tris-HCl pH 7.4, 5 mM MgCl_2_, 100 mM KCl, 1% Triton X-100, Protease inhibitor, 2 mM DTT, 100 mg/mL cycloheximide, and RNase inhibitor). After centrifugation at 5000 × *g* at 4 °C for 10 min, 10% supernatant was taken as an input sample, and the remainder was loaded on top of a 10–50% sucrose gradient and centrifuged at 30,000 × *g* at 4 °C for 3 h. After removing the top lysis layer, and RNA was extracted from the ribosomal pellet layer using TRIzol. *ACTN4* and *GAPDH* levels were measured using RT-qPCR, and *GAPDH* was set as the control.

### Co-IP assay

Huh7 cells were washed three times with PBS after collected and lysed with lysis buffer (50 mM Tris-HCl pH 7.5, 1 mM EGTA, 1 mM EDTA, 1% Triton X-100, 150 mM NaCl, 2 mM DTT) supplemented with protease inhibitors on ice. After centrifugation at 13,000 × *g* for 10 min, 5% supernatant was taken as input, and the remainder was incubated with indicated Abs overnight at 4 °C. After incubating the mixture with Protein G agarose at 4 °C for 2 h, the beads were washed with washing buffer and then with IP buffer three times each. Finally, the co-IP and input samples were denatured and subjected to western blotting.

### CCK-8 cell viability assay

Huh7 cells were inoculated into 96-well plates at 20,000 cells/well, then treated with the indicated concentrations of wortmannin, YS-49, or demethyl-coclaurine for 24 h. Cell viability was tested using a CCK-8 Kit (A311; Vazyme) according manufacturer’s protocols. The data were plotted via GraphPad Prism 8.

### Mouse experiments, hematoxylin and eosin staining, and immunohistochemistry

Eight-week-old, male transgenic K18-hACE2 mice (C57BL/6JGpt-H11^em1cin(K18-hACE2)^/Gpt (Strain NO. T037657)) were randomly divided into groups with at least five animals in each group. The anti-viral effects of YS-49 and demethyl-coclaurine were investigated using weight monitoring, virus detection (RT-qPCR, focus-forming assay, immunostaining analysis), histopathology, and survival analysis. Mice were inoculated with 1 × 10^5^ pfu SARS-CoV-2 BA.5 strains nasally, and the mice were intragastrically administrated daily at a drug dose of 50 mg/kg according to the body weight. Genomic RNA of secreted viruses was isolated from tissue samples using the QlAamp Viral RNA Mini Kit (52906; Qiagen) as suggested by the manufacturer, and quantified by RT-qPCR using a Taqman probe targeting the SARS-CoV-2 *N* gene (same forward and reverse primers as mentioned above, fluorescence probe: 5′-FAM-TTGCTGCTGCTTGACAGATT-TAMRA-3′). The data were plotted using GraphPad Prism 8.

Hematoxylin and eosin staining and immunohistochemical analysis were performed at Wuhan Bioqiandu Technology. Briefly, mouse lung and brain sections were fixed in 4% paraformaldehyde, embedded in paraffin, sectioned, and stained with hematoxylin and eosin. For immunohistochemistry, slides were blocked in 3% bovine serum albumin for 30 min and incubated with a primary Abs against SARS-CoV-2 N protein (#26369; Cell Signaling Technology) in blocking buffer at 4 °C overnight. After washing with PBS three times, the slides were incubated with cy3-conjugated secondary Abs at room temperature for 50 min, and nuclei were counterstained with DAPI. Images were acquired using Pannoramic DESK, P-MIDI, and P250 (3D HISTECH).

### Statistical analysis

Data are presented as means ± standard error of the means (SEMs) or standard deviations (SDs) (*n* = 3). All experiments were repeated at least three times. RT-qPCR data were statistically analyzed using a two-tailed unpaired *t*-test and one-way ANOVA in GraphPad Prism Software (version 5). *P* ≤ 0.05 was considered statistically significant.

## Supplementary information

Supplementary Materials

WB original image

## Data Availability

SARS-CoV-2 sequence data analyzed in this study were uploaded to indicated servers: GISAID (https://www.gisaid.org/) with accession numbers EPI_ISL_402124, EPI_ISL_402127-EPI_ISL_402130 and EPI_ISL_402131; GenBank with accession numbers MN996527 to MN996532; National Genomics Data Center in Beijing Institute of Genomics, CAS (https://bigd.big.ac.cn/databases?lang=en) with accession numbers SAMC133236-SAMC133240 and SAMC133252. The second-generation sequencing data in Fig. 1d were deposited in GSA (http://bigd.big.ac.cn) with accession number CRA003624. The MeRIP-seq data in Fig. 1f were uploaded to GEO (https://www.ncbi.nlm.nih.gov/geo/) with accession number GSE274550.
