# Supplementary material for: Characterization of ACTN4 as a novel antiviral target against SARS-CoV-2
Source: Signal Transduct Target Ther. 2024 Sep 18;9:243. doi: 10.1038/s41392-024-01956-4 (PMC11408661; doi:10.1038/s41392-024-01956-4)
Supplement: Supplementary file 1 — Supplementary Materials [file 41392_2024_1956_MOESM1_ESM.docx]

Supplementary Materials for

**Characterization of ACTN4 as a novel antiviral target against SARS-CoV-2**

Miao Zhu^1,2^, Fang Huang^3^, Huize Sun^1,2^, Kunpeng Liu^1,2^, Zhen Chen^1^, Baocheng Yu^1,2^, Haojie Hao^1^, Haizhou Liu^1^, Shuang Ding^1^, Xueyan Zhang^1^, Lishi Liu^1,2^, Kui Zhang^1,2^, Jierao Rao^1,2^, Yi Liu^3^, Haibin Liu^1,3^, Chao Shan^1,3^ and Wuxiang Guan^1,3^*

* Correspondence: Wuxiang Guan, Tel.: +86-27-87197258; Fax: +86-27 87197258; Email: [guanwx@wh.iov.cn](mailto:guanwx@wh.iov.cn)

These authors contributed equally: Miao Zhu, Fang Huang, Huize Sun.

**This PDF file includes:**

Supplementary Fig. 1 to 12

Supplementary Table 1

Supplementary Fig. 1.


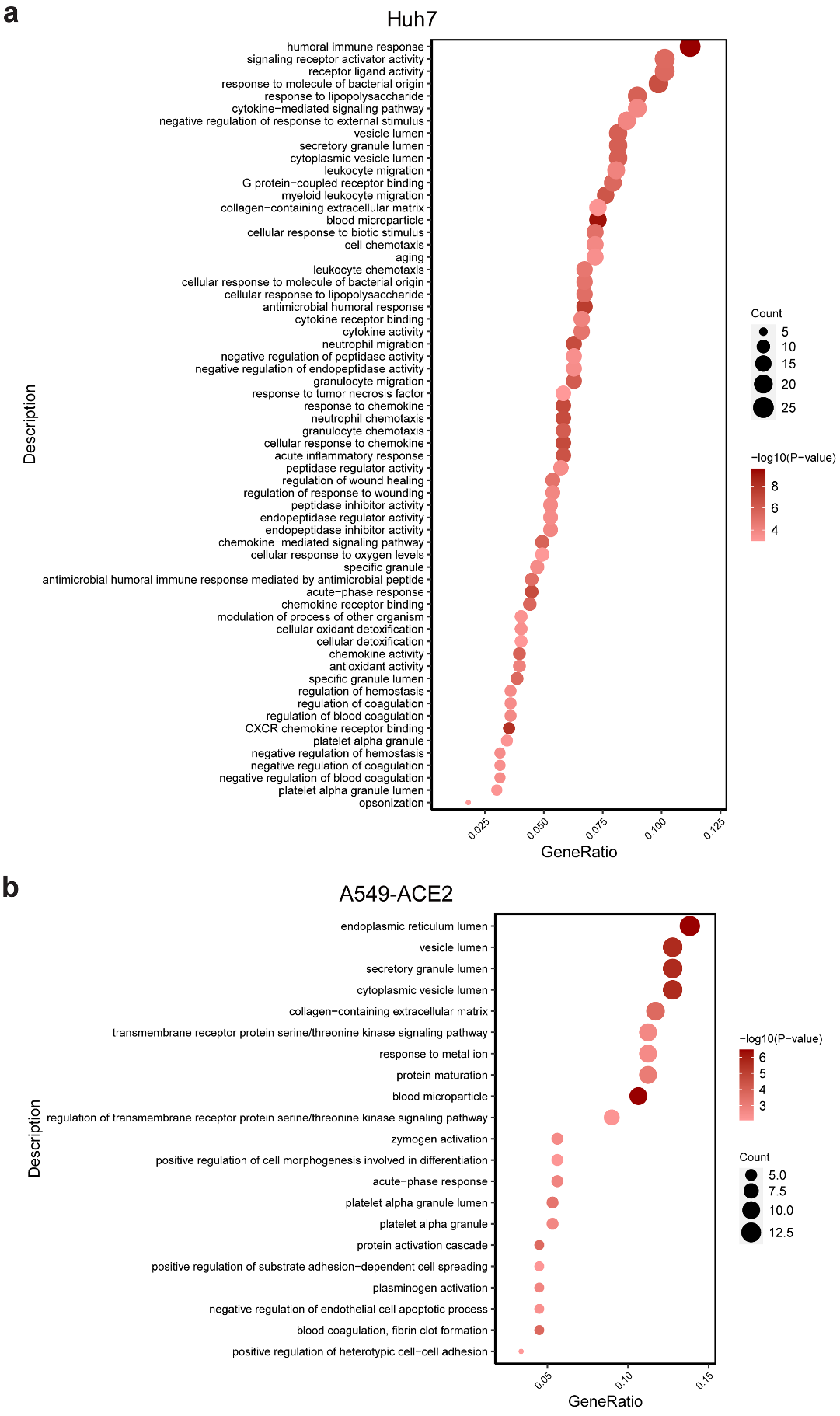


**Supplementary Fig. 1 SARS-CoV-2 infection alters the pattern of multiple cellular pathways in cells (related to Fig. 1)**

(a, b) GO pathway enrichment differentially expressed genes in Huh7 cells (a) and A549-ACE2 cells (b).

Supplementary Fig. 2.


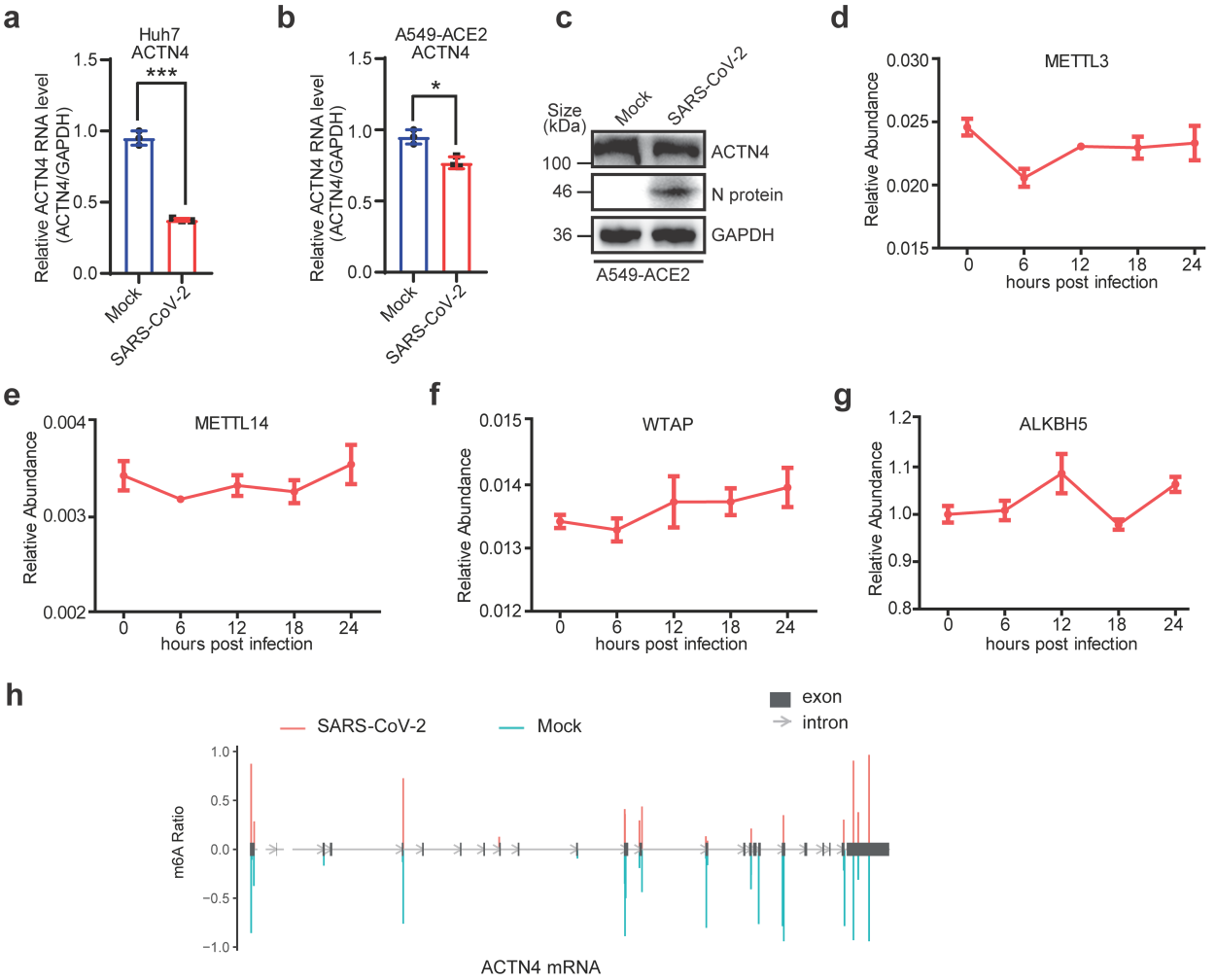


**Supplementary Fig. 2 SARS-CoV-2 infection decreases ACTN4 expression in cells (related to Fig. 1)**

(a, b) RT-qPCR analysis of *ACTN4* mRNA levels in Huh7 (a) and A549-ACE2 (b) cells infected with WT SARS-CoV-2 at MOI = 1 for 48 h. Data are means ± SEMs (*n =* 3). **P* ≤ 0.05, ****P* < 0.001, unpaired Student’s *t*-test. (c) Western blot (WB) assays of ACTN4 expression, viral N protein and GAPDH in A549-ACE2 cells infected with WT SARS-CoV-2 at MOI = 1 for 48 h were performed using indicated antibodies (Abs). (d-g) Relative abundances of m6A-related proteins at different time points, METTL3 (d), METTL14 (e), WTAP (f) and ALKBH5 (g) were analyzed in Huh7 cells infected with WT SARS-CoV-2 at MOI = 1. (h) Distribution of m6A modification levels on ACTN4 mRNAs. Changes of m6A modification levels on ACTN4 mRNAs in Huh7 cells with SARS-CoV-2 infection or not were presented. Blue represents uninfected, red represents infected.

Supplementary Fig. 3.


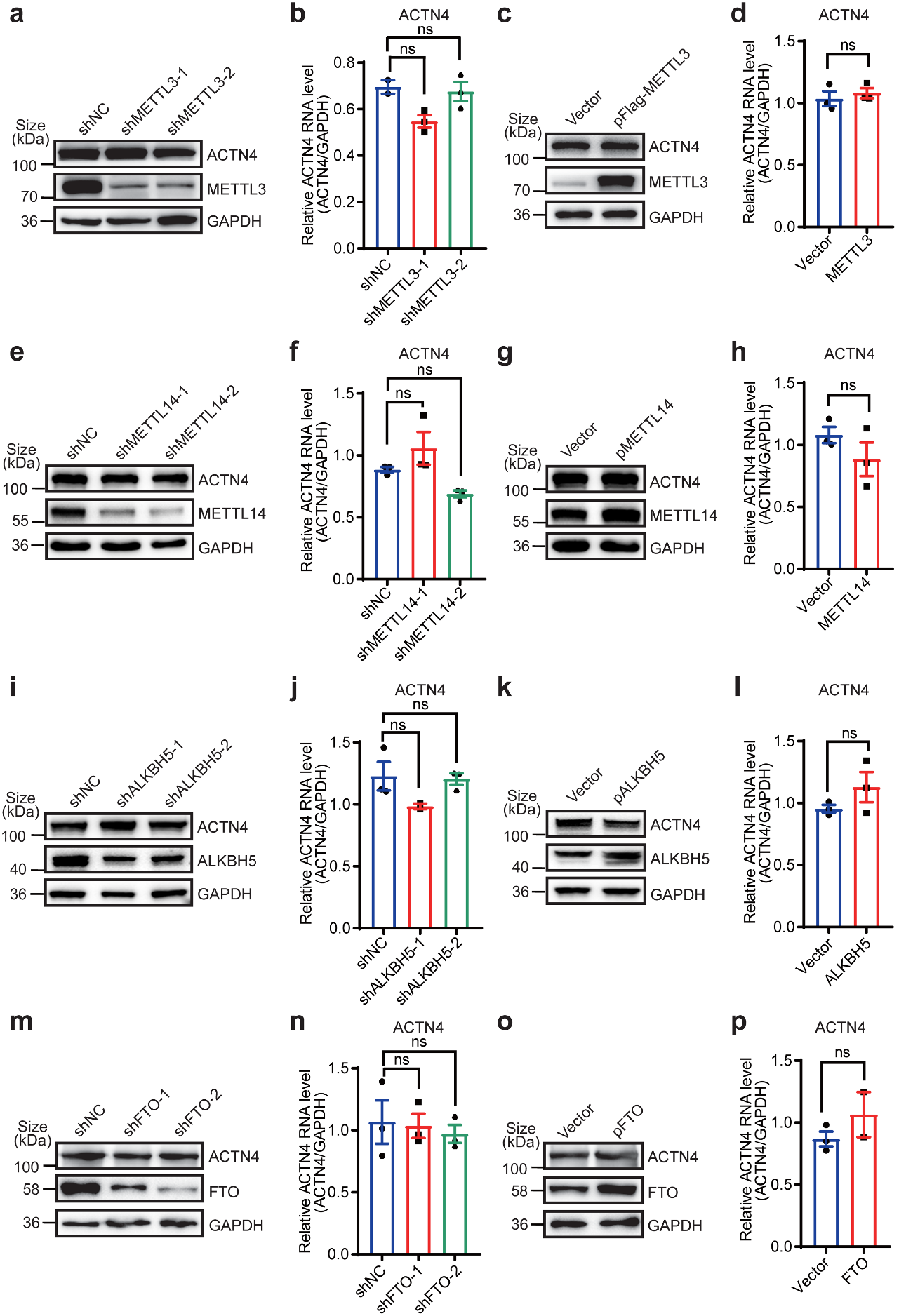


**Supplementary Fig. 3 Effect of m6A related machinery on ACTN4 expression (related to Fig. 2).**

(a, c, e, g, i, k, m, o) WB assays. METTL3/METTL14/ALKBH5/FTO was overexpressed (c, g, k, o) or knocked down using indicated shRNAs (a, e, i, m) in Huh7 cells. METTL3/METTL14/ALKBH5/FTO expression was analyzed using anti-METTL3/METTL14/ALKBH5/FTO Abs. Vector-transfected cells were used as a control. (b, d, f, h, j, l, n, p) RT-qPCR. Total RNAs were isolated from Huh7 cells in which METTL3/METTL14/ALKBH5/FTO was overexpressed (d, h, l, p) or knocked down using indicated shRNAs (b, f, j, n). Relative levels of *ACTN4* mRNA were quantified using RT-qPCR using specific primers. Data are means ± SEMs (*n =* 3). ns: not significant, unpaired Student’s *t*-test (d, h, l, p) or one-way ANOVA (b, f, j, n).

Supplementary Fig. 4.


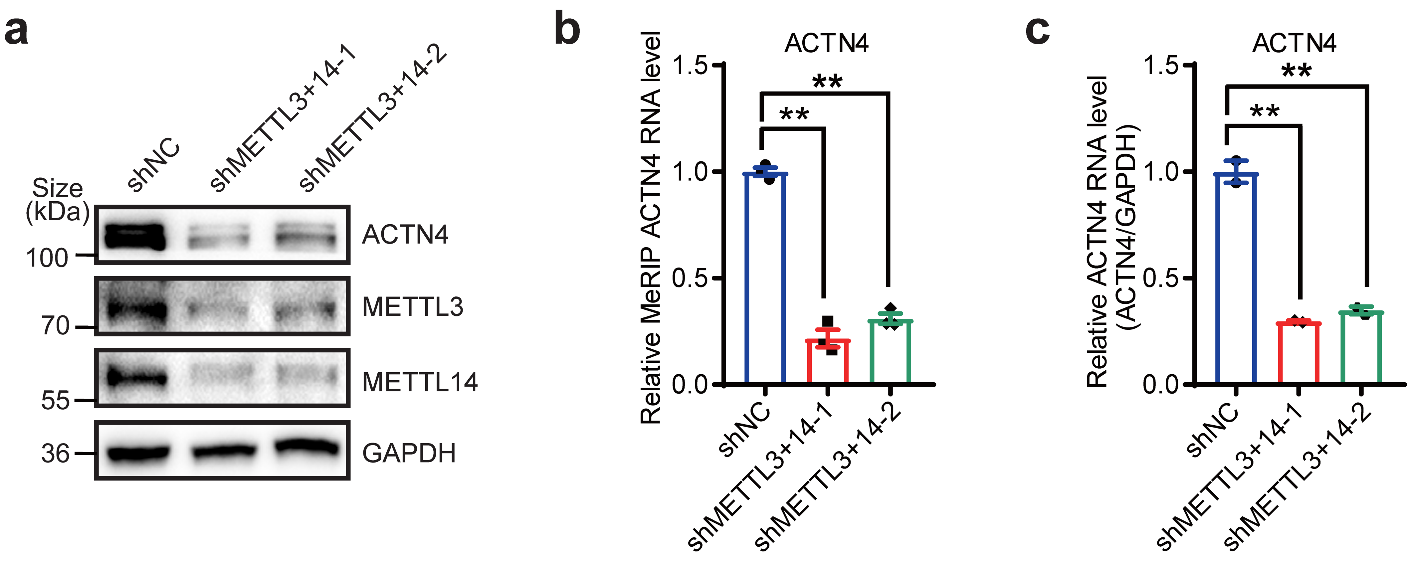


**Supplementary Fig. 4 Knockdown of both METTL3 and METTL14 affects the expression of ACTN4 (related to Fig. 2).**

(a) WB assays. Huh7 cells with knockdown of both METTL3 and METTL14 were constructed. The expression of METTL3/METTL14/ACTN4 was detected by WB assays using anti- METTL3/METTL14/ACTN4 Abs, and GAPDH was set as the control. (b) MeRIP-qPCR. IP assays were performed with m6A Abs in Huh7 cells with knockdown of both METTL3 and METTL14, and qRT-PCR was used to detect the relative abundance of m6A enriched ACTN4 mRNA. (c) RT-qPCR. Total RNAs were isolated from Huh7 cells with knockdown of both METTL3 and METTL14 using indicated shRNAs. Relative levels of *ACTN4* mRNA were quantified using RT-qPCR using specific primers. Data are means ± SEMs (*n =* 3). ***P* ≤ 0.01, one-way ANOVA.

Supplementary Fig. 5.


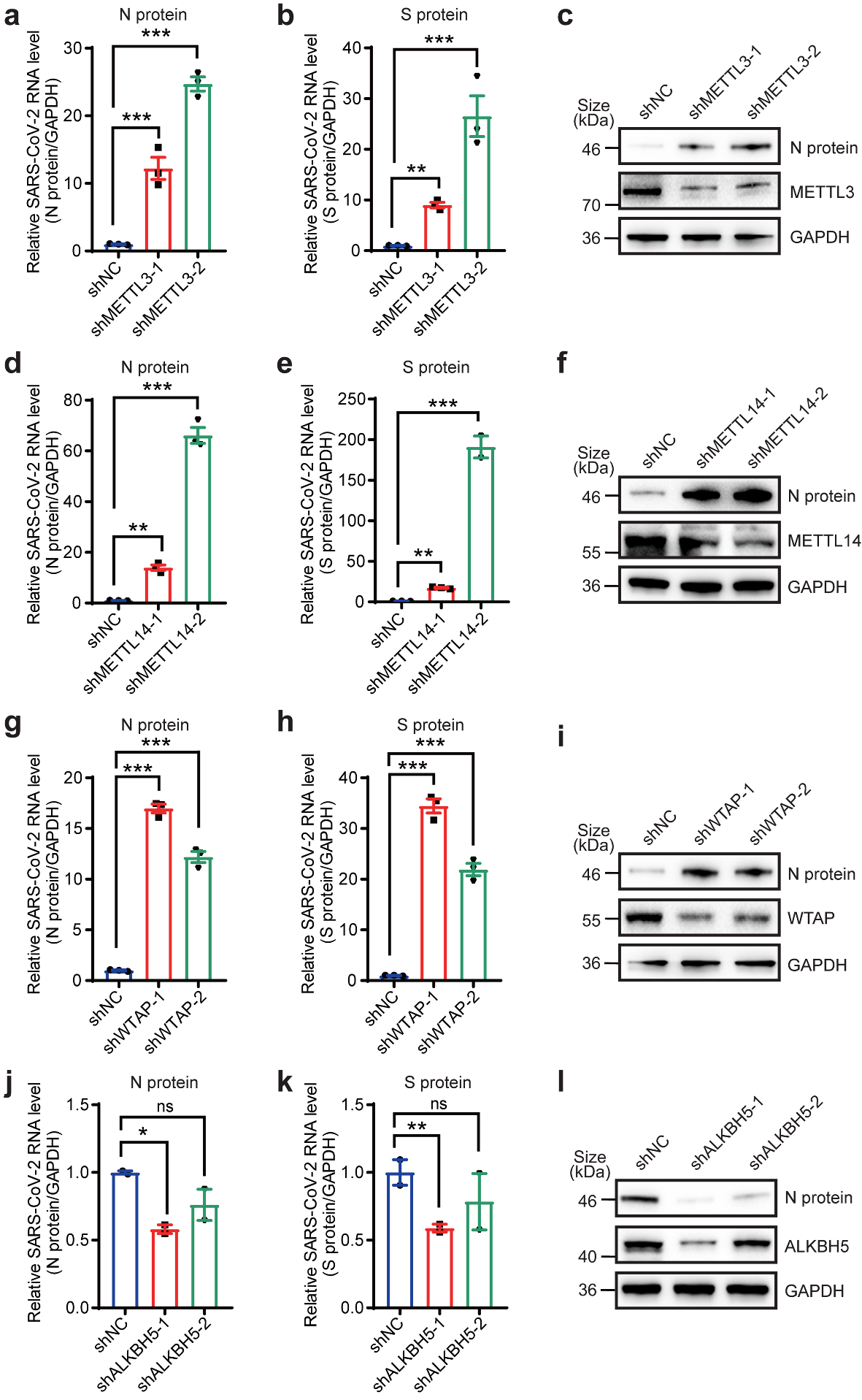


**Supplementary Fig. 5 The m6A related host factors regulate SARS-CoV-2 replication (related to Fig. 2).**

RT-qPCR was performed to determine the SARS-CoV-2 WT RNA levels in METTL3 (a, b), METTL14 (d, e), WTAP (g, h) and ALKBH5 (j, k) knockdown Huh7 cells infected with WT SARS-CoV-2 at MOI = 1 for 48 h. Relative SARS-CoV-2 RNA levels were quantified with specific primers targeting the *N* gene (a, d, g, j) or *S* gene (b, e, h, k). Data are means ± SEMs (*n =* 3). **P* ≤ 0.05, ***P* ≤ 0.01, ****P* < 0.001, ns: not significant, one-way ANOVA. (c, f, i, l) The expression of SARS-CoV-2 N proteins was detected by anti-N protein Abs. METTL3/METTL14/WTAP/ALKBH5 expression was analyzed by WB assays using anti-METTL3/METTL14/WTAP/ALKBH5 Abs. GAPDH was set as the control.

Supplementary Fig. 6.


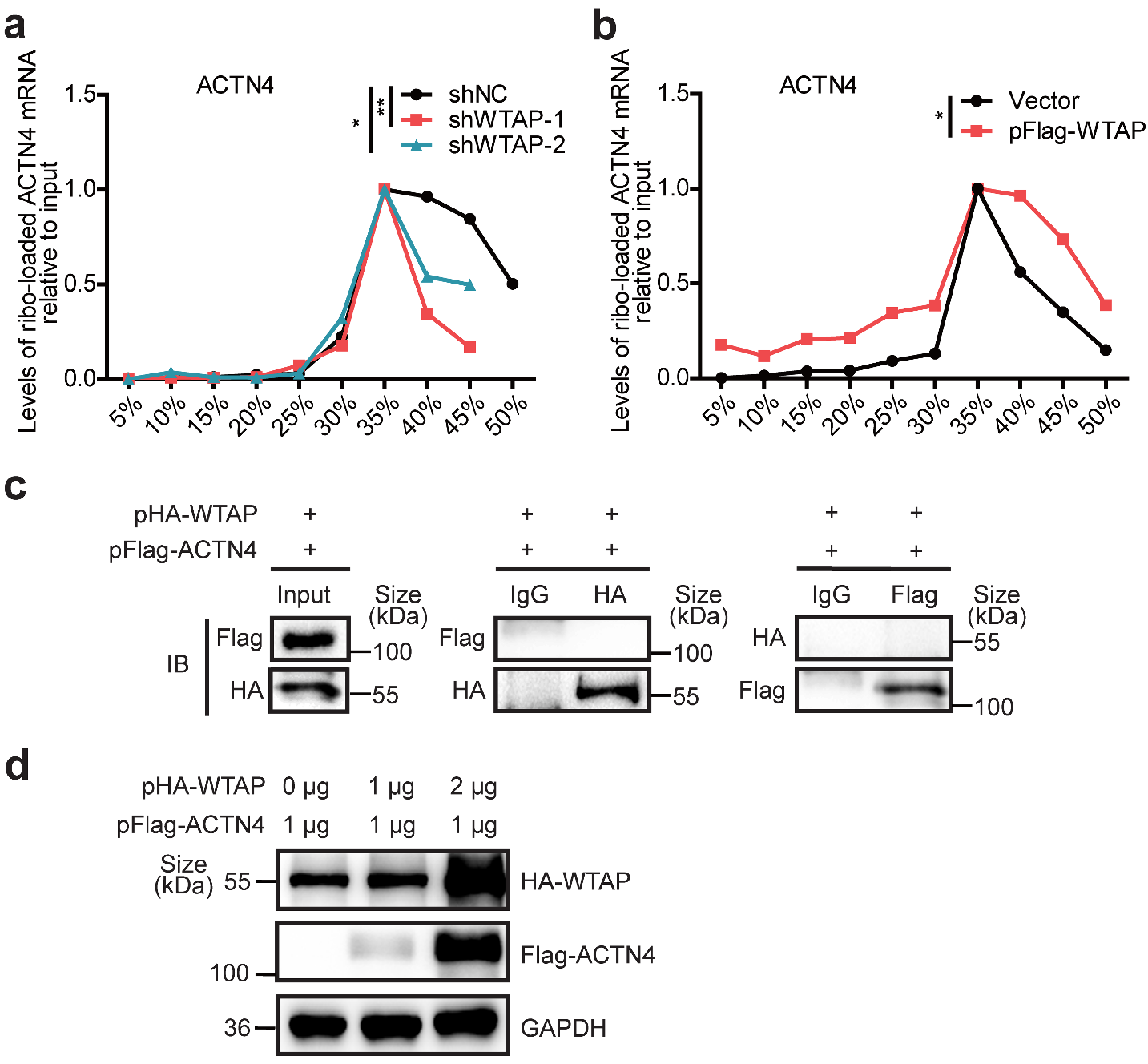


**Supplementary Fig. 6 WTAP does not interact with ACTN4, but promotes its expression (related to Fig. 2).**

(a, b) Huh7 cells with knockdown of WTAP (a) or overexpressed WTAP (b) were lysed, the pre-cleared lysates were pelleted on top of density gradient (5%-50%) sucrose and proceeded to ultra-centrifugation at 35,000 g, 4 h, 4 °C. RNA was extracted from each layer, qRT-PCR was used to detect the abundance of *ACTN4* mRNA. The relative levels of ribosome loaded ACTN4 mRNA were presented as the line charts. Data are means ± SEMs (*n =* 3). **P* ≤ 0.05, ***P* ≤ 0.01, unpaired Student’s t-test (b) or one-way ANOVA (a). (c) WTAP did not interact with ACTN4. pFlag-ACTN4 and pHA-WTAP were co-transfected into Huh7 cells, and Co-IP was performed using anti-HA (middle) or anti-Flag (right) Abs. IgG was set as the control. Left was input. The immuno-blots were probed with the anti-Flag or anti-HA Abs. (d) WTAP promotes ACTN4 expression in a dose-dependent manner. Huh7 cells were transfected with pFlag-ACTN4 and increasing amounts of pHA-WTAP. Levels of ACTN4 and WTAP were detected by WB assays using indicated Abs.

Supplementary Fig. 7.


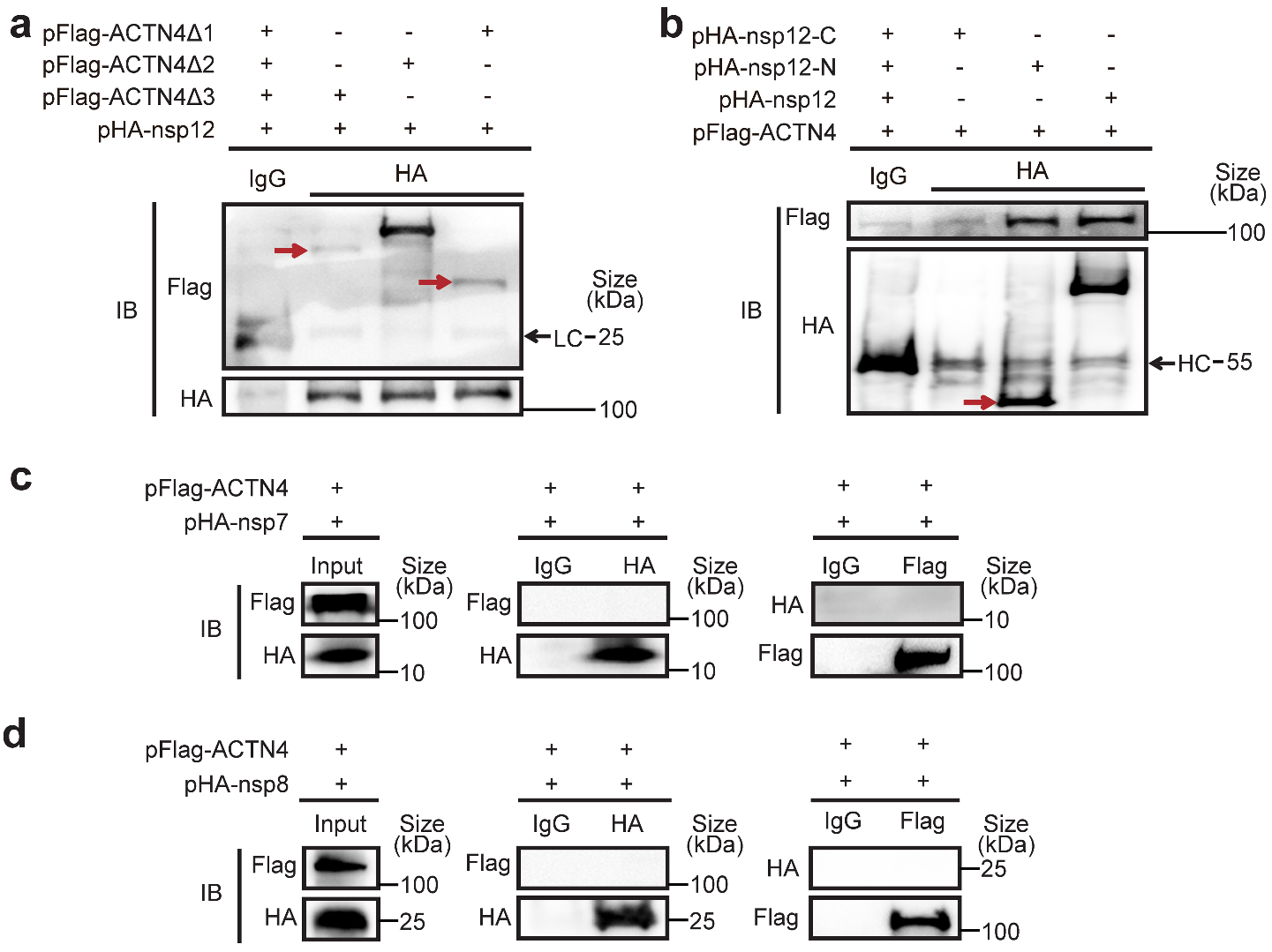


**Supplementary Fig. 7 Domains of ACTN4 and nap12 responsible for their interactions were mapped (related to Fig. 4)**. (a, b) Co-immunoprecipitation (Co-IP) was performed using anti-HA Abs, IgG was set as the control. The immuno-blots were probed with the anti-Flag or anti-HA Abs. ‘HC’ represents heavy chain, ‘LC’ represents light chain. (c, d) ACTN4 does not interact with nsp7 or nsp8. Huh7 cells were co-transfected with pFlag-ACTN4 and pHA-nsp7 (c) or pHA-nsp8 (d). Co-IP was performed using anti-HA (middle) or anti-Flag (right) Abs. IgG was set as the control. Left was input. The immuno-blots were probed with the anti-Flag or anti-HA Abs.

Supplementary Fig. 8.


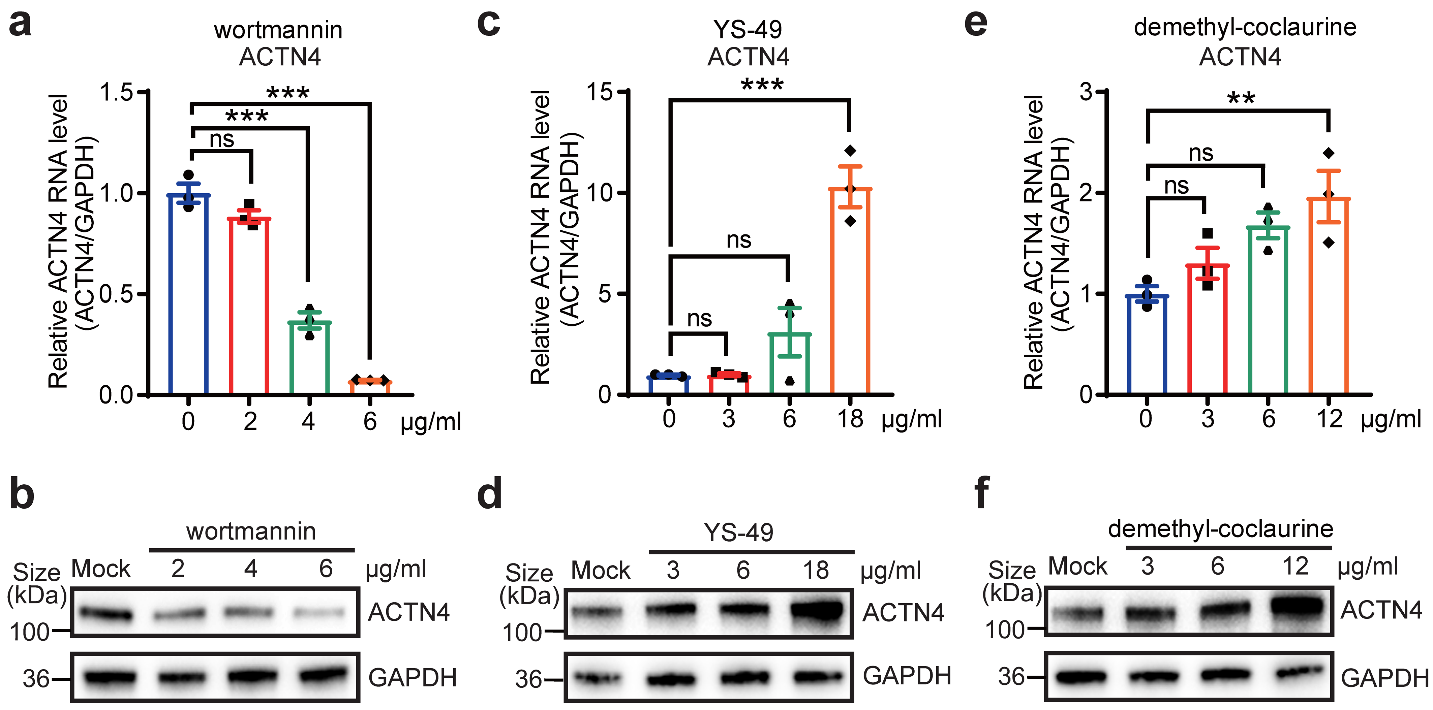


**Supplementary Fig. 8** **ACTN4 inhibitors and agonists affect ACTN4 expression (related to Fig. 5).**

(a, c, e) Wortmannin inhibited ACTN4 transcription (a), YS-49 (c) and demethyl-coclaurine (e) promoted ACTN4 transcription. RT-qPCR was used to detect relative ACTN4 mRNA levels after Huh7 cells were treated with these compounds for 48 h. Data are means ± SEMs (*n =* 3). ***P* ≤ 0.01, ****P* < 0.001, ns: not significant, one-way ANOVA. (b, d, f) WB assays of ACTN4 expression in Huh7 cells after treated with wortmannin (b), YS-49 (d) and demethyl-coclaurine (f) were conducted using anti-ACTN4 Abs. GAPDH was set as the control.

Supplementary Fig. 9.


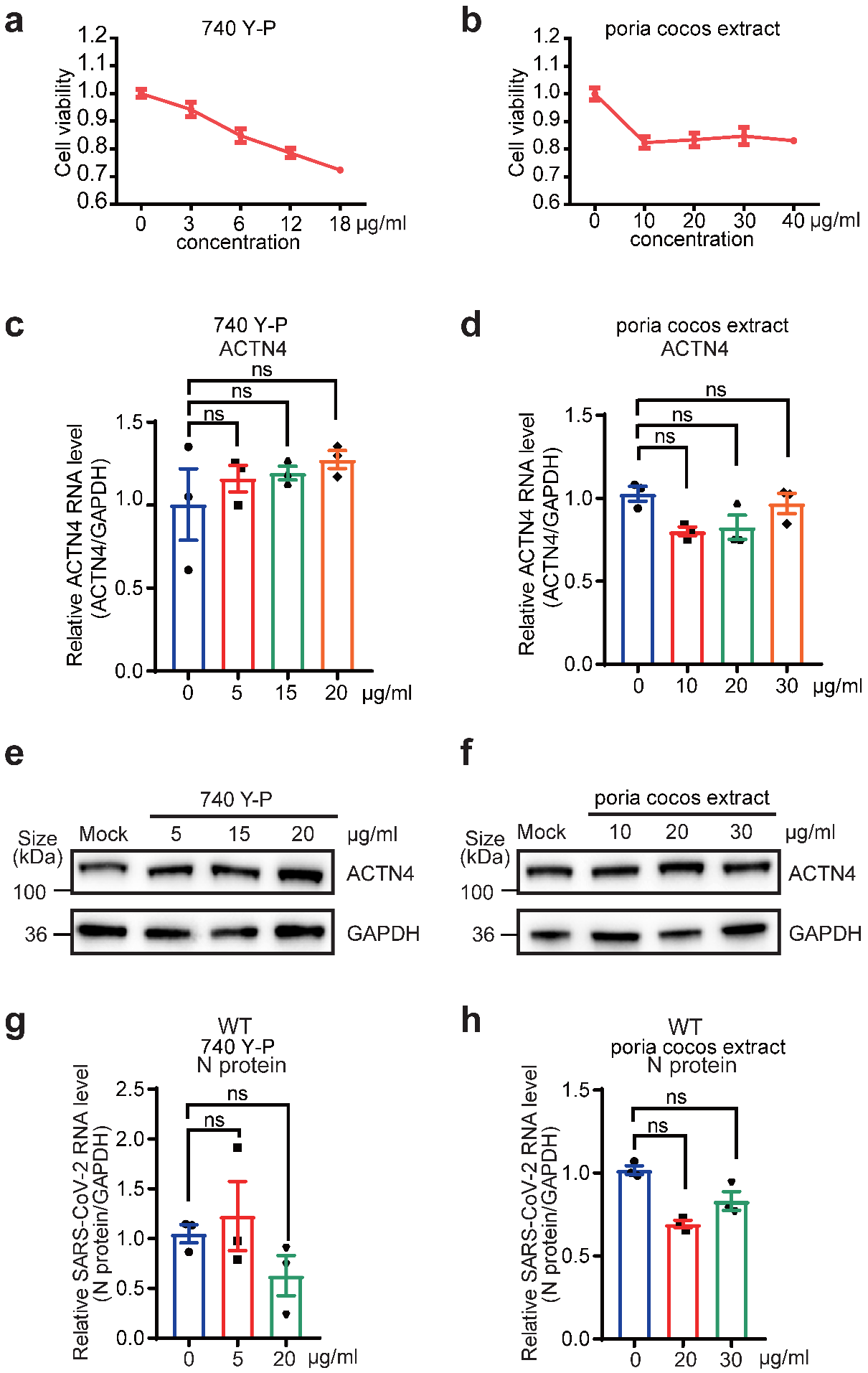


**Supplementary Fig. 9** **The PI3K agonists 740 Y-P and poria cocos extract affect neither the expression of ACTN4 nor the WT SARS-CoV-2 replication (related to Fig. 5).**

(a, b) Huh7 cells were treated with increasing amounts of 740 Y-P (a) or poria cocos extract (b) for 48 h. Cell viability was assessed by the CCK-8 assay. Error bars denote mean ± sd of 3 independent replicates. (c, d) RT-qPCR was used to detect relative ACTN4 mRNA levels after Huh7 cells treated with 740 Y-P (c) and poria cocos extract (d) for 48 h. Data are means ± SEMs (*n =* 3). ns: not significant, one-way ANOVA. (e, f) The ACTN4 expression in Huh7 cells after treated with 740 Y-P (e) and poria cocos extract (f) was detected by WB assays using anti-ACTN4 Abs. GAPDH was set as the control. (g, h) Huh7 cells were treated with increasing amounts of 740 Y-P (g) or poria cocos extract (h) for 24 h, then infected with WT SARS-CoV-2 at MOI =1 for another 48 h. The relative viral RNA levels were measured using RT-qPCR with specific primers targeting the *N* gene, Data are means ± SEMs (*n =* 3). ns: not significant, one-way ANOVA.

Supplementary Fig. 10.


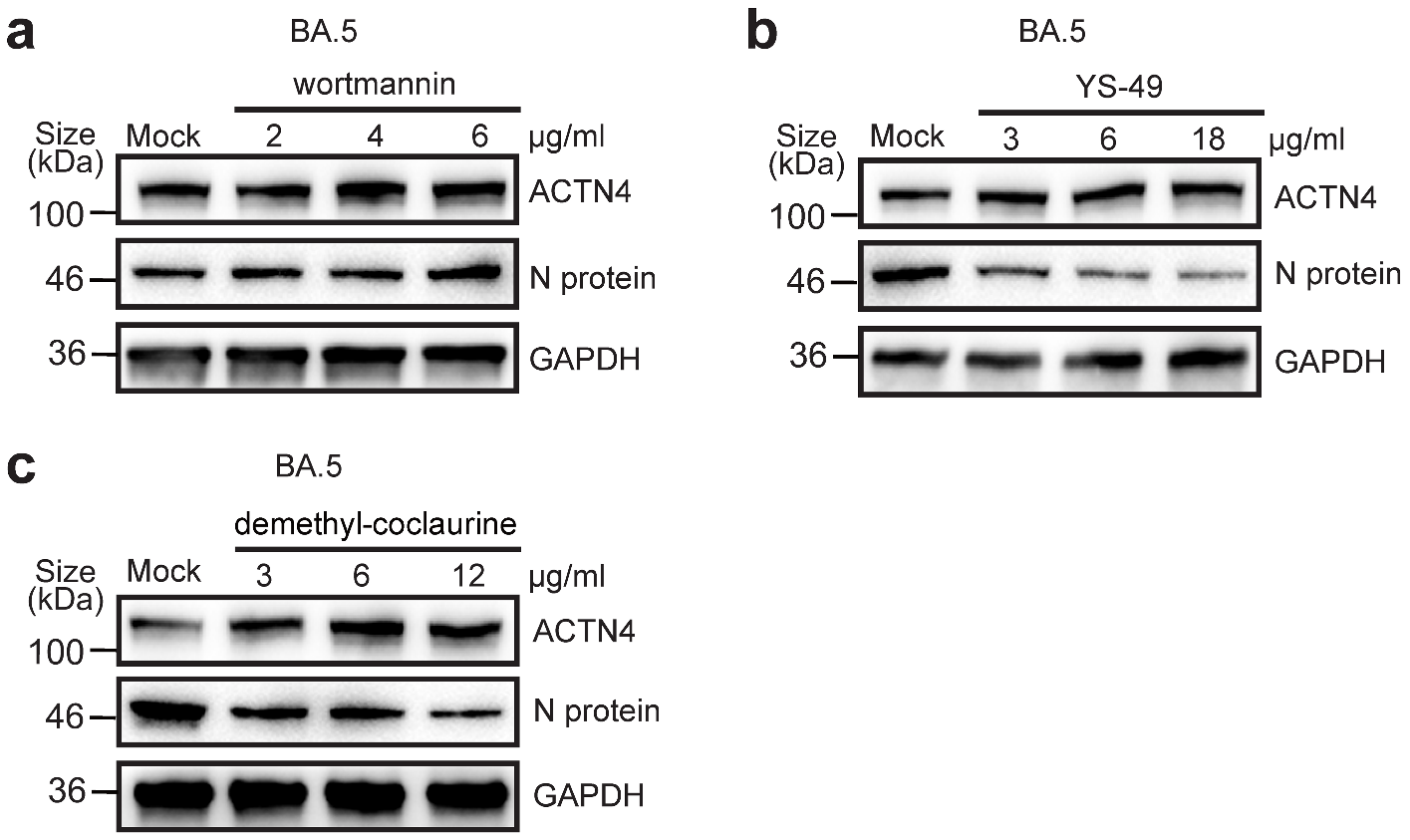


**Supplementary Fig. 10** **The inhibitors and agonists of ACTN4 affect the expression of the Omicron BA.5 variant encoded N protein (related to Fig. 5).**

(a-c) Huh7 cells were treated with increasing amounts of wortmannin, YS-49 or demethyl-coclaurine for 24 h, then infected with Omicron BA.5 variant at MOI =1 for another 48 h. Cells were lysed and subjected into WB assays. Levels of ACTN4, N protein and GAPDH were detected using the indicated Abs.

Supplementary Fig. 11.


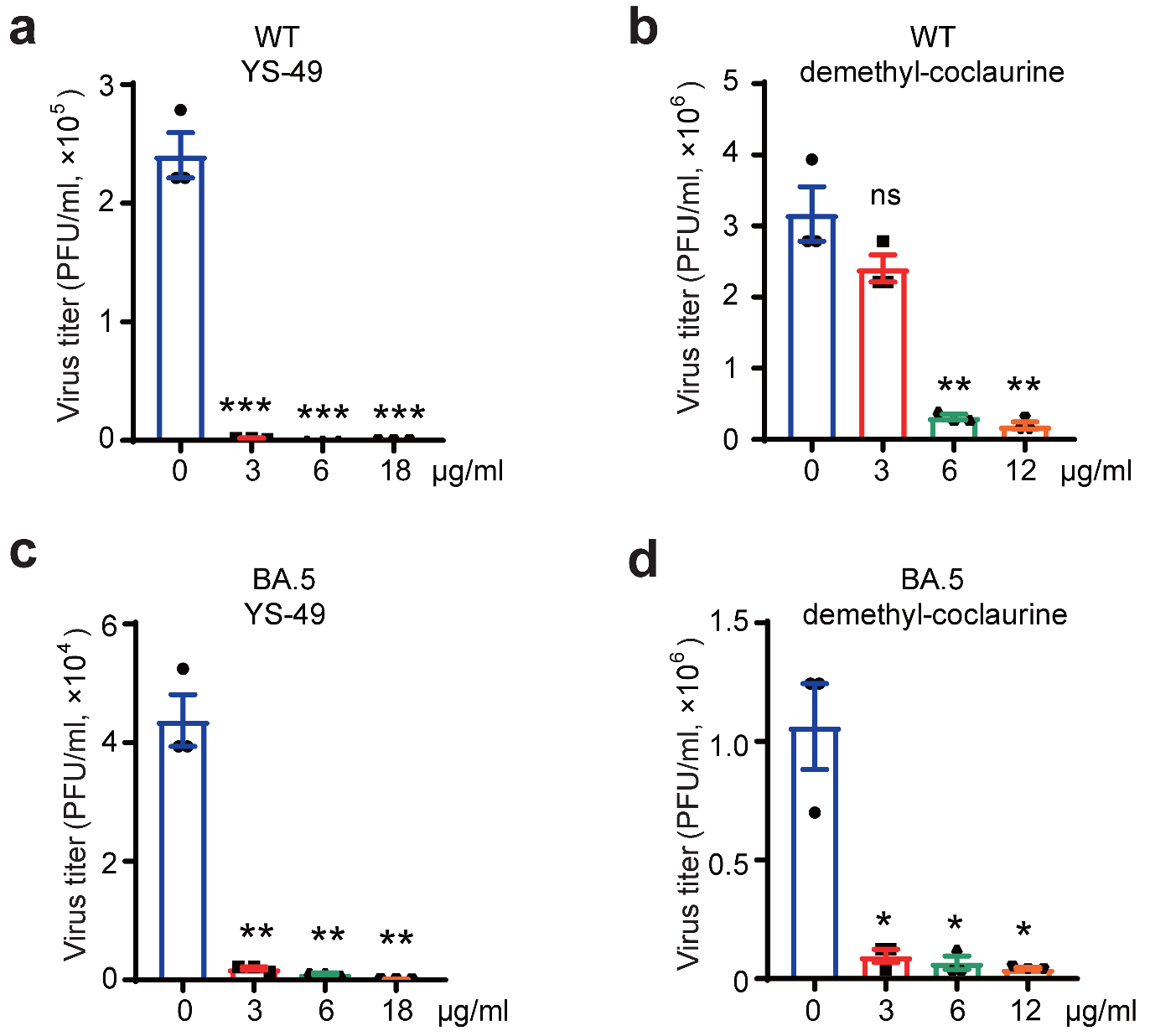


**Supplementary Fig. 11** **Effects on virus titer by ACTN4 agonists in Huh7 cells were measured (related to Fig. 5).**

Plaque assay was performed to detect SARS-CoV-2 WT (a, b) or BA.5 (c, d) titer affected by YS-49 (a, c) and demethyl-coclaurine (b, d). Data are means ± SEMs (*n =* 3). **P* ≤ 0.05, ***P* ≤ 0.01, ****P* < 0.001, ns: not significant, one-way ANOVA.

Supplementary Fig. 12.


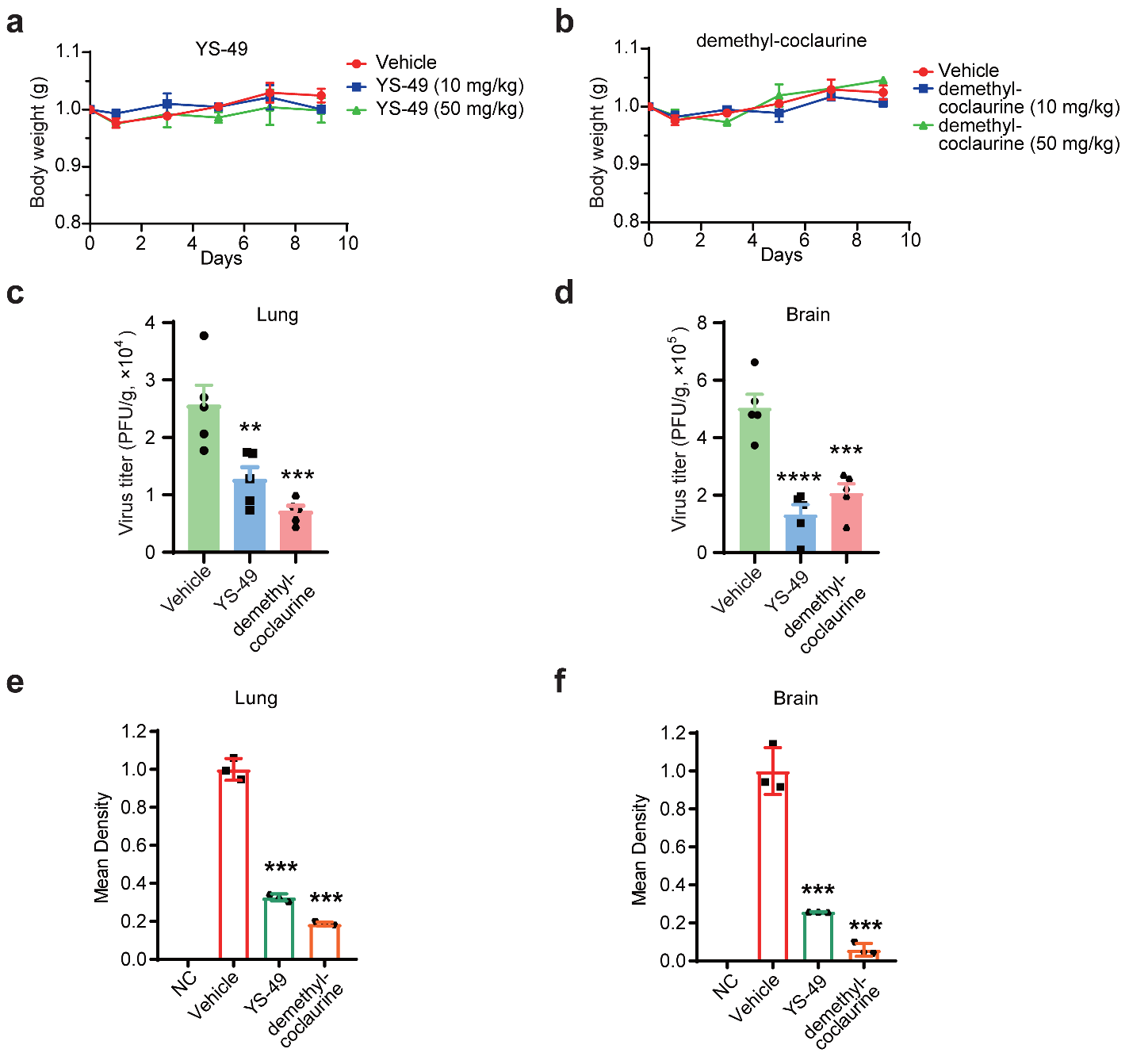


**Supplementary Fig. 12 Effects of ACTN4 agonists against SARS-CoV-2 infection to mice were measured (related to Fig. 6).**

(a, b) Mice were treated with different amounts (10 mg/kg and 50 mg/kg) of YS-49 (a) or demethyl-coclaurine (b) or DMSO (Vehicle) once a day, the body weights were recorded daily, the data presented as the line charts. Error bars denote mean ± sd of 3 independent replicates. (c, d) Effects on virus titer by ACTN4 agonists in mice tissues. Virus titer in lung (c) and brain (d) tissue were detected by plaque assay. Data are means ± SEMs (*n =* 3). ***P* ≤ 0.01, ****P* < 0.001, *****P* < 0.0001, one-way ANOVA. (e, f) The statistical results of the SARS-CoV-2 N proteins in lung and brain tissues using immunofluorescence from the Fig. 6h, i. Data are means ± SEMs. ****P* < 0.001, one-way ANOVA.

| ID | NBL3-2023067 | |  |  |  |  |  | | |  |
| --- | --- | --- | --- | --- | --- | --- | --- | --- | --- | --- |
| Cage number | Group | ID | Ear tag | Autonomous activity | Neural response | hair | hunchback | Breath | Total points | Note |
| 2 | Vehicle | DC-1 | 1473 | 0.5 | 0.5 | 0.5 | 0.5 | 0 | 2 |  |
|  |  | DC-2 | 1464 | 0.5 | 0.5 | 0.5 | 0.5 | 0 | 2 |  |
|  |  | DC-3 | 1462 | 0.5 | 0.5 | 0.5 | 0.5 | 0 | 2 | Poop in the right eye |
|  |  | DC-4 | 1494 | 0.5 | 0.5 | 0.5 | 0.5 | 0 | 2 |  |
|  |  | DC-5 | 1472 | 0.5 | 0.5 | 0.5 | 0.5 | 0 | 2 |  |
| 3 | YS-49 | YS-1 | 1499 | 0.5 | 0.5 | 0.5 | 0 | 0 | 1.5 |  |
|  |  | YS-2 | 1467 | 0.5 | 0.5 | 0.5 | 0.5 | 0 | 2 |  |
|  |  | YS-3 |  | 0.5 | 05 | 0.5 | 0 | 0 | 1.5 |  |
|  |  | YS-4 | 1459 | 1 | 1 | 0.5 | 0 | 0 | 2.5 |  |
|  |  | YS-5 | 1465 | 1 | 1 | 0.5 | 0 | 0 | 2.5 |  |
| 4 | demethyl-coclaurine | Co-1 | 870 | 0.5 | 0.5 | 0.5 | 0 | 0 | 1.5 |  |
|  |  | Co-2 |  | 0.5 | 0.5 | 0.5 | 0 | 0 | 1.5 |  |
|  |  | Co-3 | 687 | 0.5 | 0.5 | 0.5 | 0 | 0 | 1.5 |  |
|  |  | Co-4 | 1479 | 0.5 | 0.5 | 0.5 | 0 | 0 | 1.5 |  |
|  |  | Co-5 | 875 | 0.5 | 0.5 | 05 | 0 | 0 | 1.5 |  |
| (Speed of movement, normal slowness, ability to turn over) (touch response, normal/slow) (normal/hair) (normal/hunchback) (normal/express shallow breathing) (The score is between 0 and 2) | | | | | | | | | | |
|  |  |  |  |  |  |  |  |  |  |  |

**Supplementary Table 1.**

Health is scored on a scale of 0-10, with 2 points for each item, 0 being normal and 10 being dying or died. The test items included: 1. the autonomic activity status of mice (normal or slow movement speed, whether they could turn over); 2. the state of nervous response (normal or slow touch response); 3. the hair state (state or fried hair); 4. the hunchback posture (normal or hunchback); 5. the breathing status (normal or express shallow breathing).
