# Supplementary material for: Characterization of ACTN4 as a novel antiviral target against SARS-CoV-2
Source: Signal Transduct Target Ther. 2024 Sep 18;9:243. doi: 10.1038/s41392-024-01956-4 (PMC11408661; doi:10.1038/s41392-024-01956-4)
Supplement: Supplementary file 2 — WB original image [file 41392_2024_1956_MOESM2_ESM.pdf]

Figure 1e

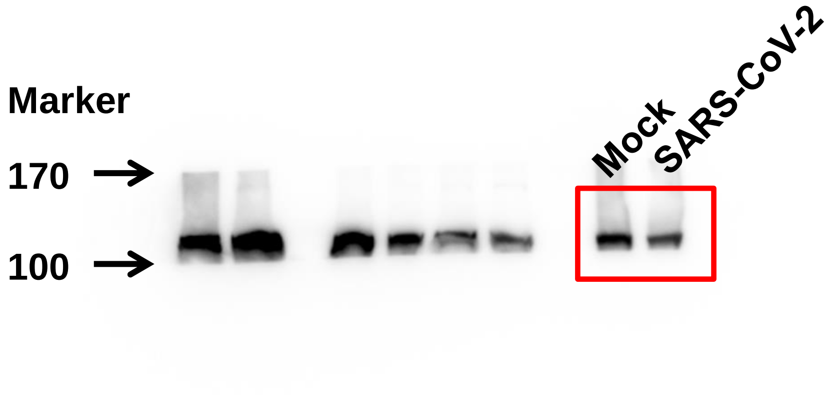

After the membrane was transferred, the membrane was cut from 100 kd, and the part of the membrane on 100 kd was incubated with **ACTN4** antibody, and band detection was performed.

Figure 1e

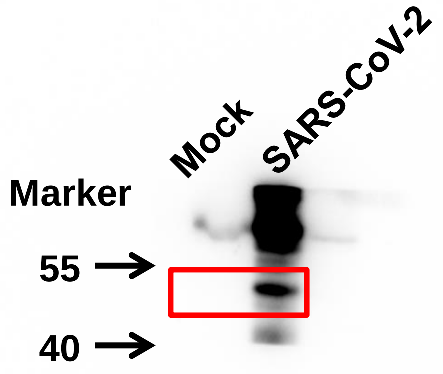

After the membrane was transferred, the membrane was cut from 40-55 kd, and the part of the membrane on 40-55 kd was incubated with **SARS-CoV-2 Nucleoprotein Rabbit pAb** antibody, and band detection was performed.

Figure 1e

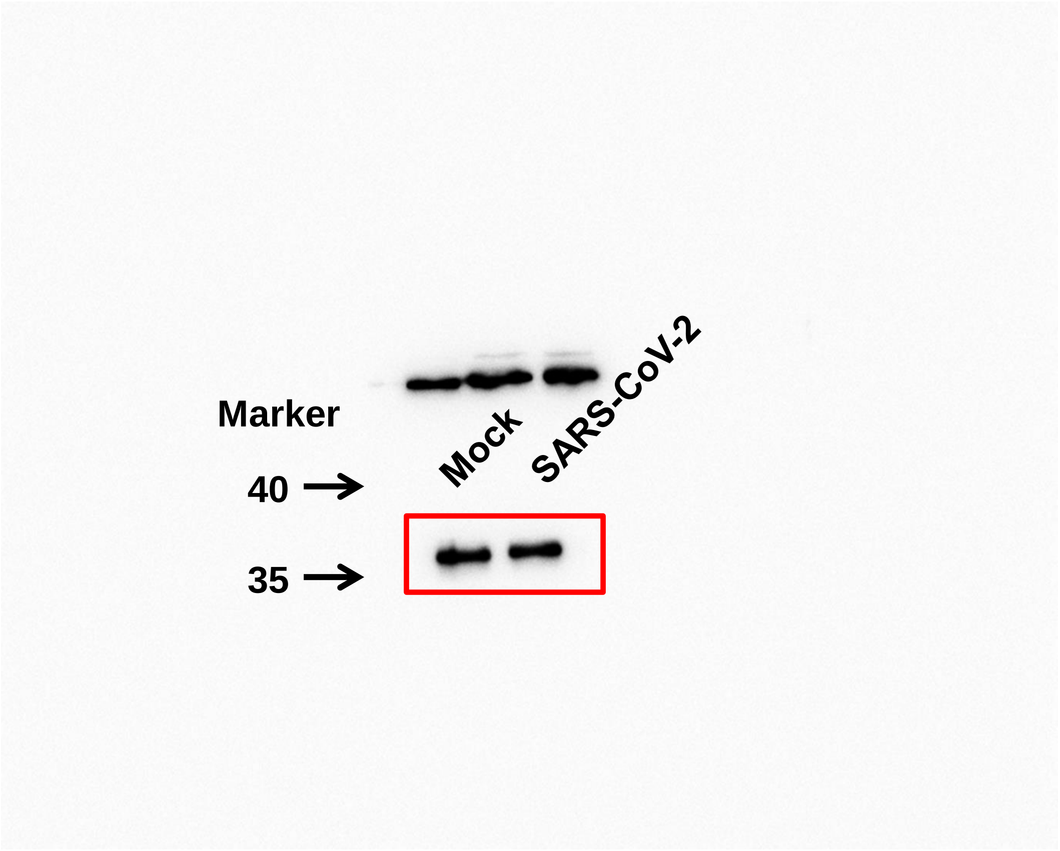

After the membrane was transferred, the membrane was cut from 35-40 kd, and the part of the membrane on 35-40 kd was incubated with **GAPDH** antibody, and band detection was performed. The two membranes were taken together.

Figure 2a

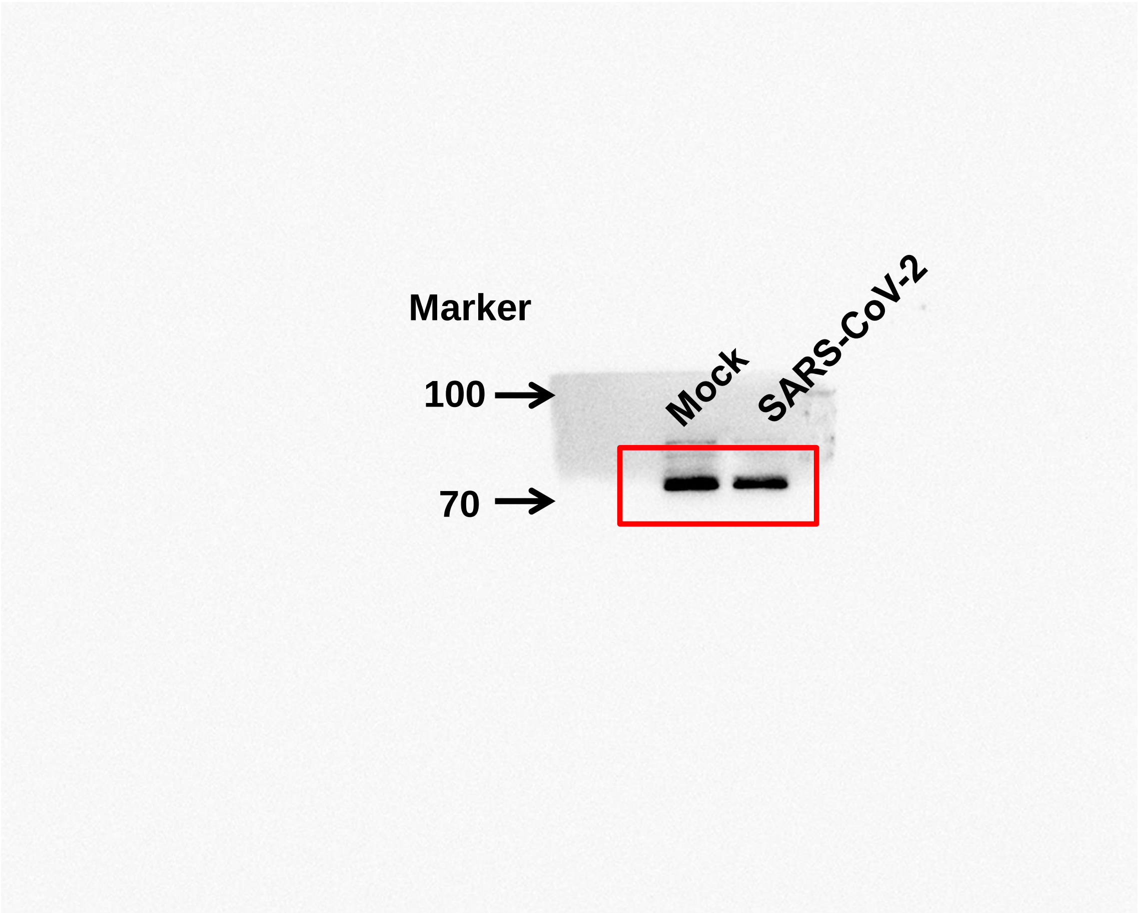

After the membrane was transferred, the membrane was cut from 70-100 kd, and the part of the membrane on 70-100 kd was incubated with **METTL3** antibody, and band detection was performed.

Figure 2a

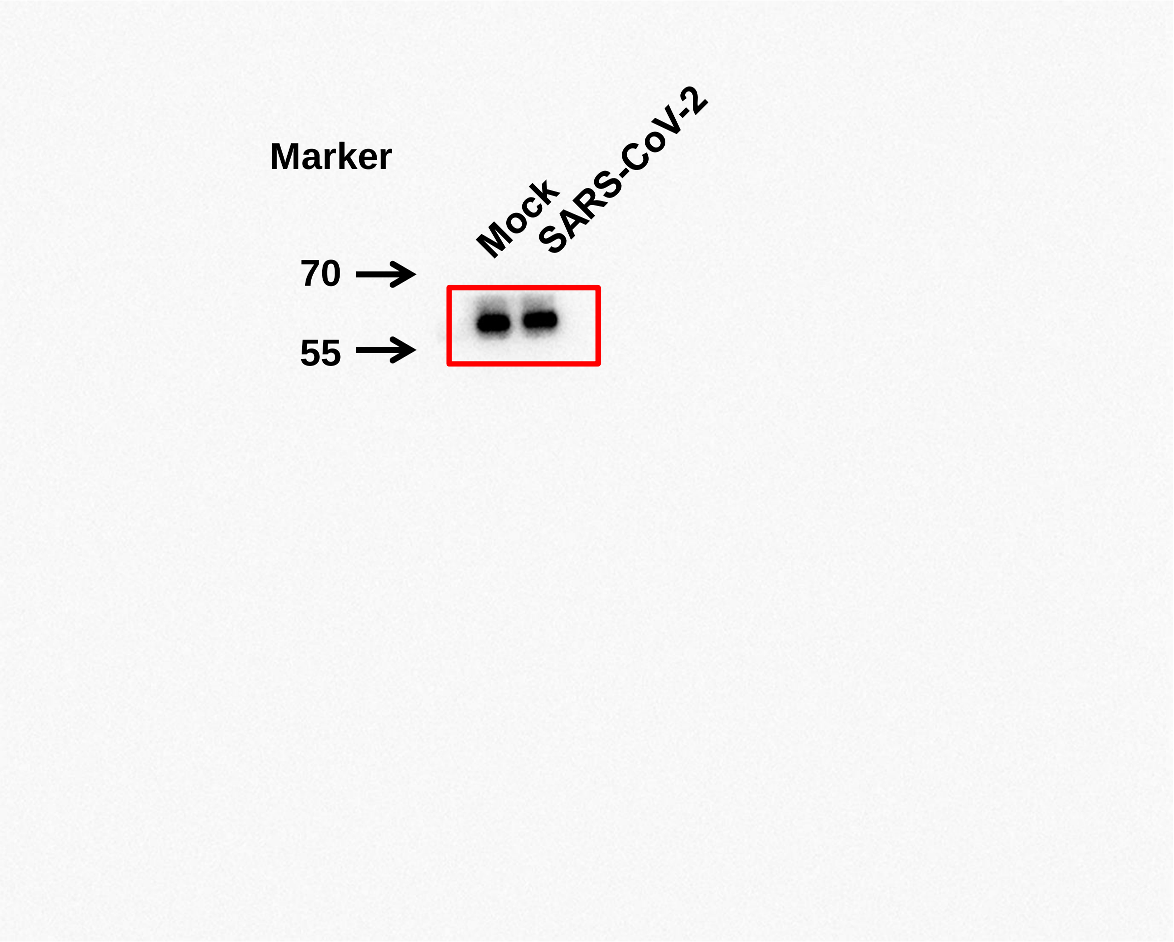

After the membrane was transferred, the membrane was cut from 55-70 kd, and the part of the membrane on 55-70 kd was incubated with **METTL14** antibody, and band detection was performed.

Figure 2a

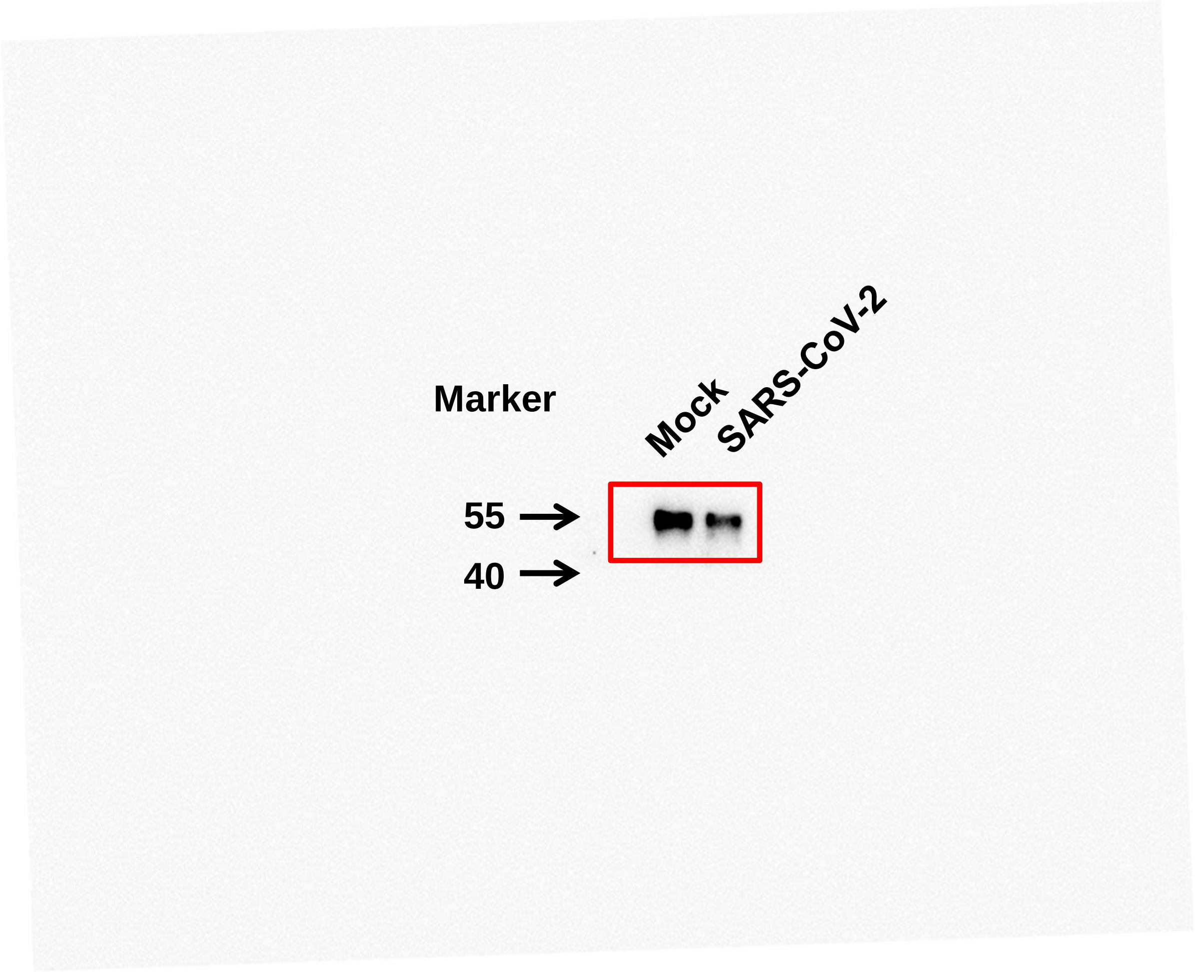

After the membrane was transferred, the membrane was cut from 40-55 kd, and the part of the membrane on 40-55 kd was incubated with **WTAP** antibody, and band detection was performed.

Figure 2a

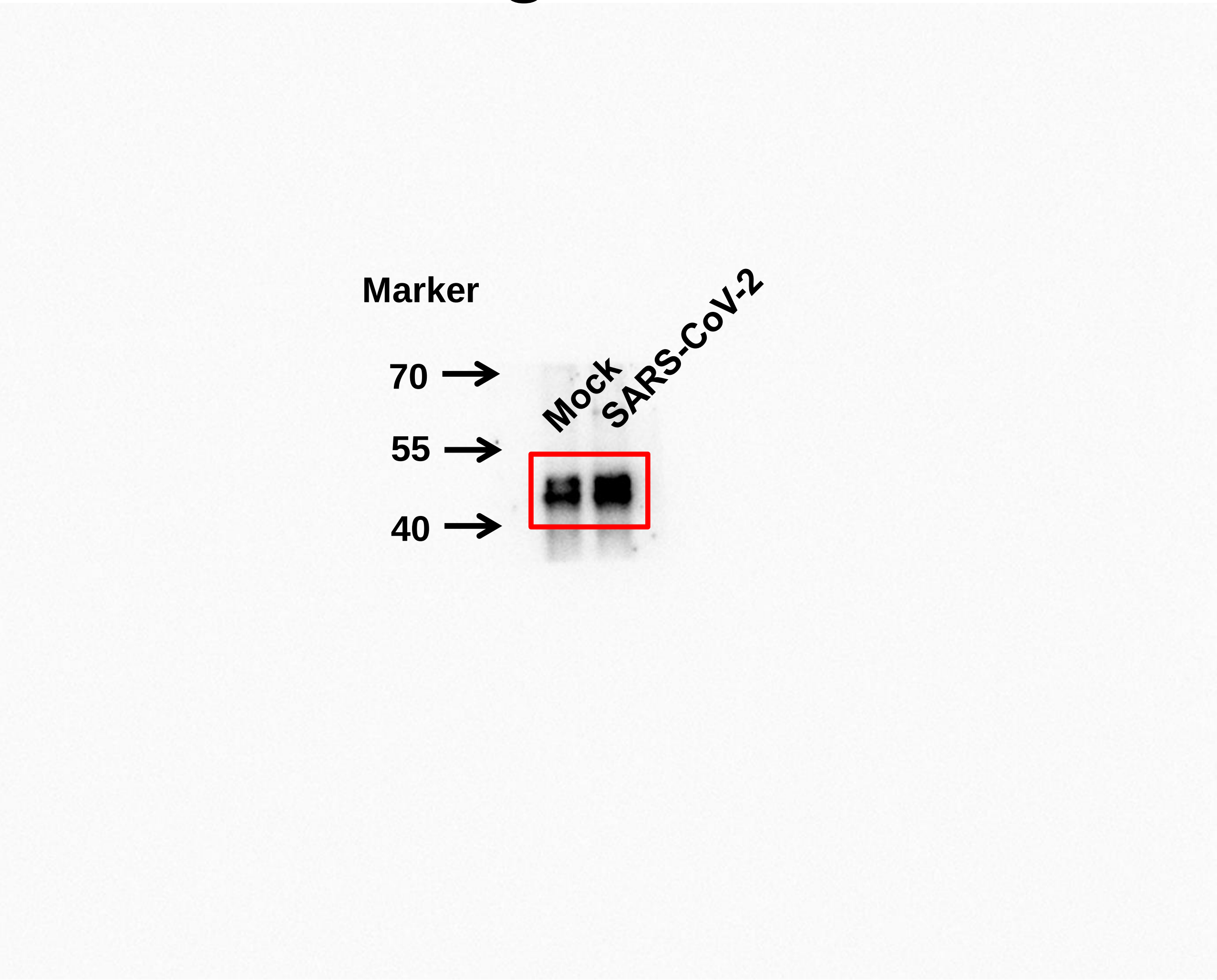

After the membrane was transferred, the membrane was cut from 40-70 kd, and the part of the membrane on 40-70 kd was incubated with **ALKBH5** antibody, and band detection was performed.

Figure 2a

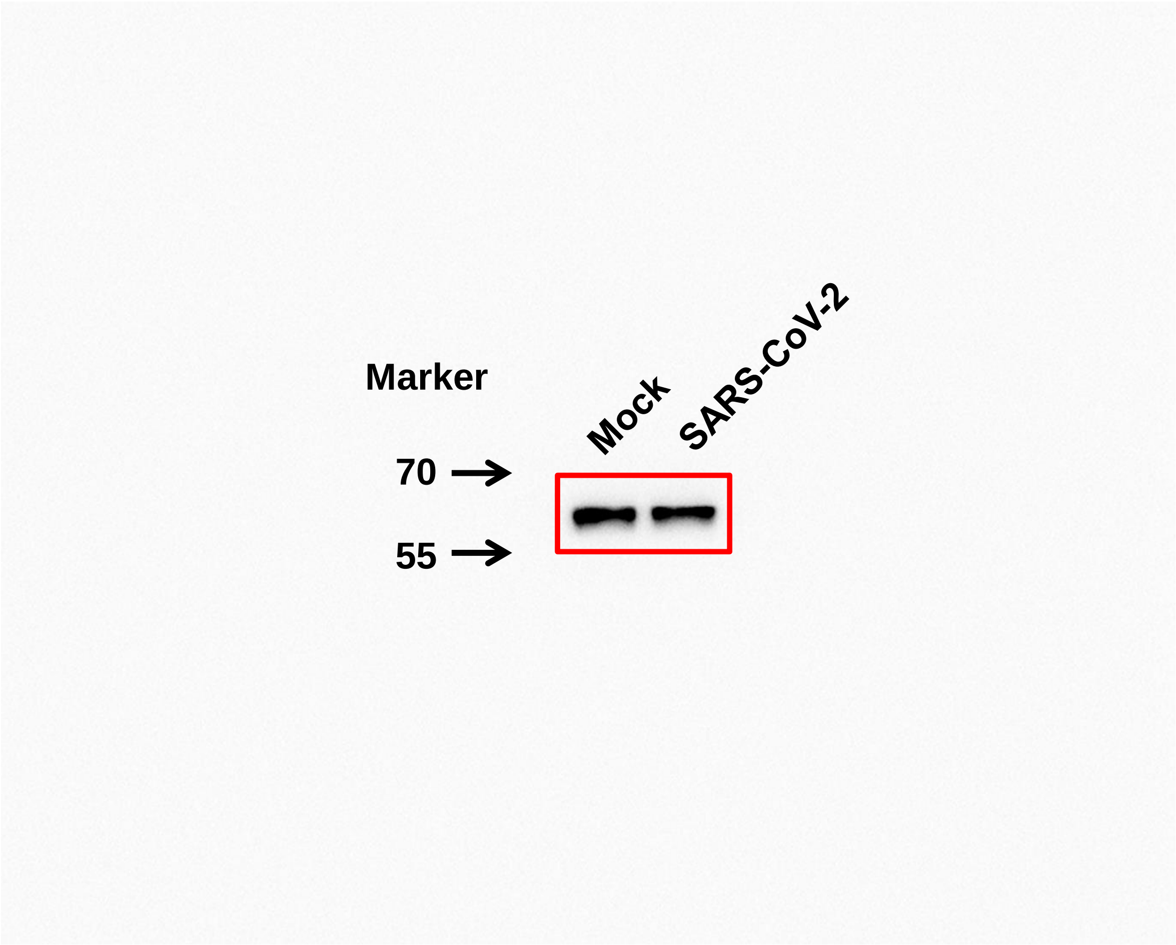

After the membrane was transferred, the membrane was cut from 55-70 kd, and the part of the membrane on 55-70 kd was incubated with **FTO** antibody, and band detection was performed.

Figure 2a

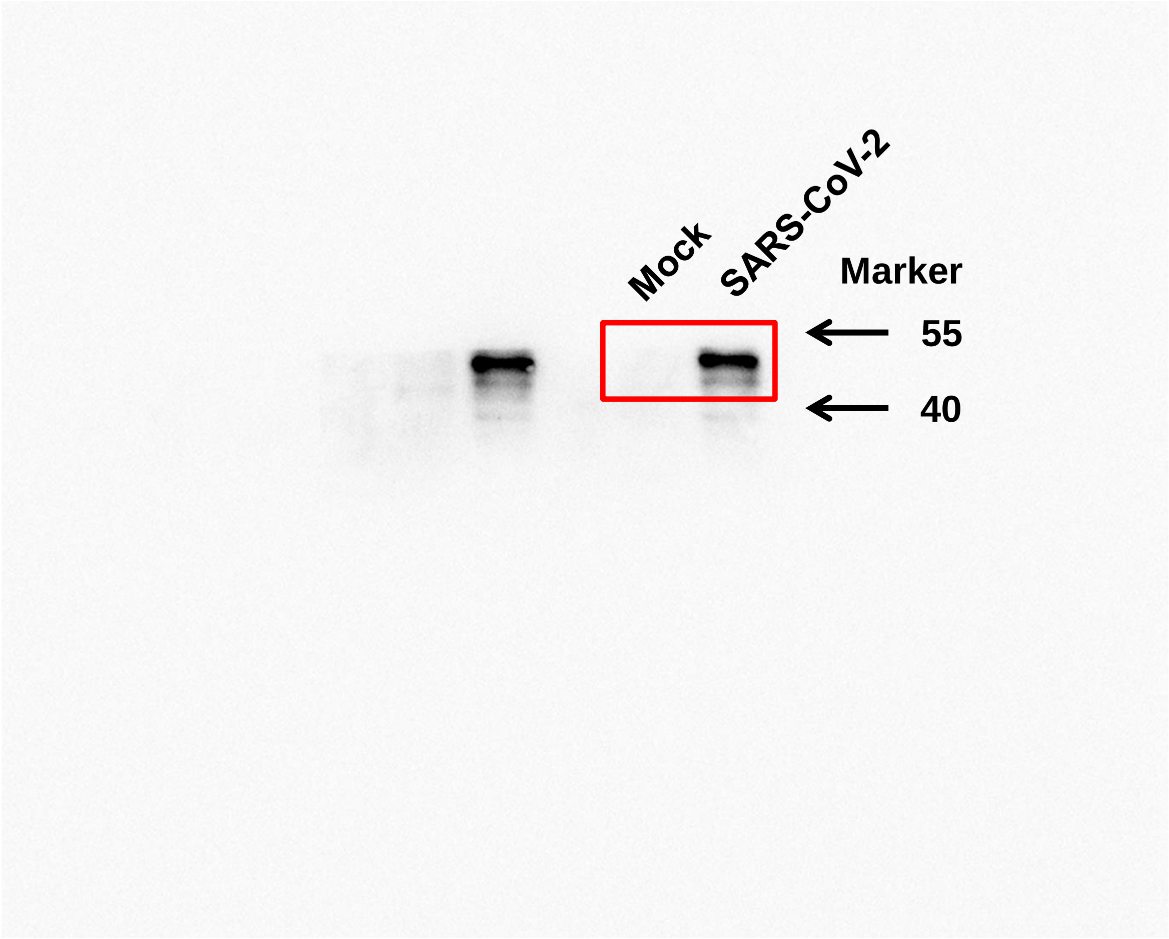

After the membrane was transferred, the membrane was cut from 40-55 kd, and the part of the membrane on 40-55 kd was incubated with **SARS-CoV-2 Nucleoprotein Rabbit pAb** antibody, and band detection was performed.

Figure 2a

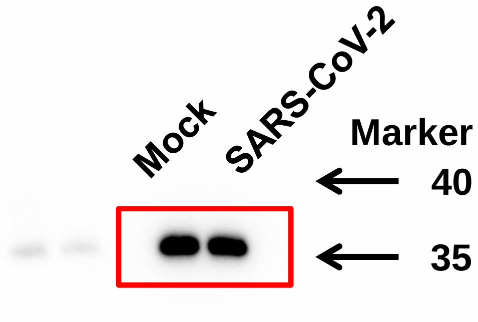

After the membrane was transferred, the membrane was cut from 35-40 kd, and the part of the membrane on 35-40 kd was incubated with **GAPDH** antibody, and band detection was performed.

Figure 2b

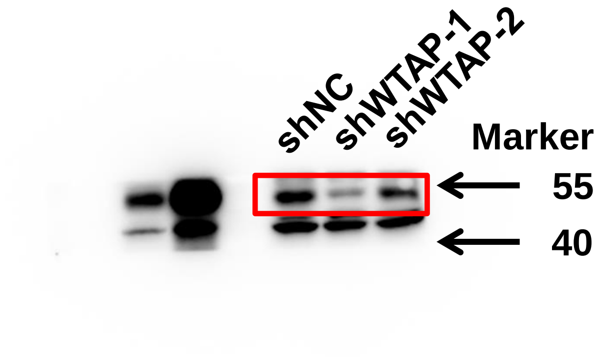

After the membrane was transferred, the membrane was cut from 40-55 kd, and the part of the membrane on 40-55 kd was incubated with **WTAP** antibody, and band detection was performed.

Figure 2b

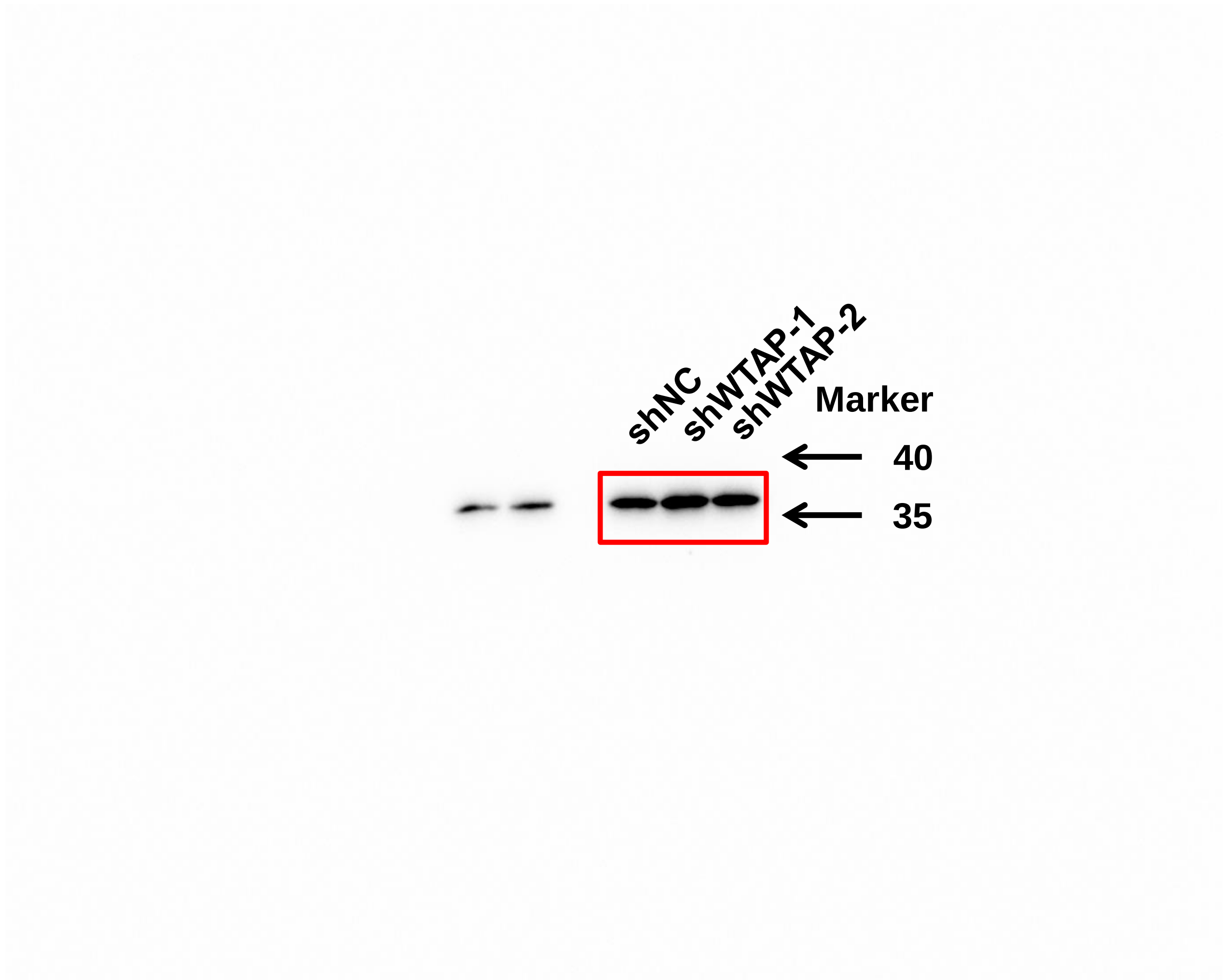

After the membrane was transferred, the membrane was cut from 35-40 kd, and the part of the membrane on 35-40 kd was incubated with **GAPDH** antibody, and band detection was performed.

Figure 2d

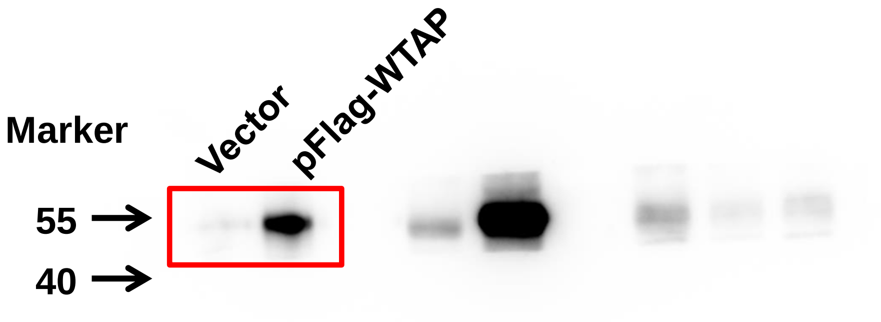

After the membrane was transferred, the membrane was cut from 40-55 kd, and the part of the membrane on 40-55 kd was incubated with **WTAP** antibody, and band detection was performed.

Figure 2d

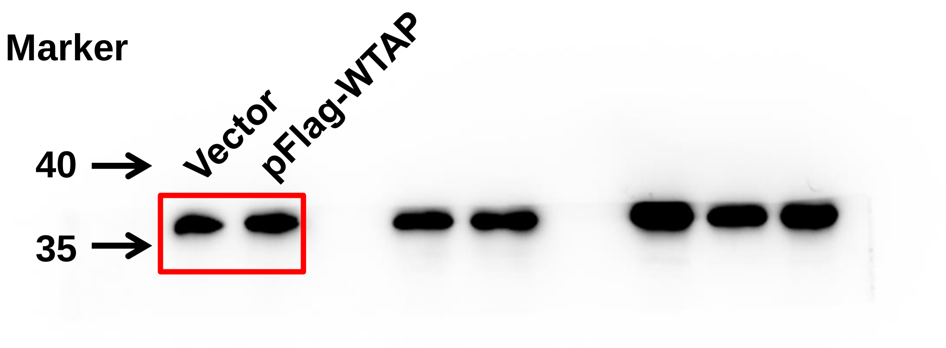

After the membrane was transferred, the membrane was cut from 35-40 kd, and the part of the membrane on 35-40 kd was incubated with **GAPDH** antibody, and band detection was performed.

Figure 2g

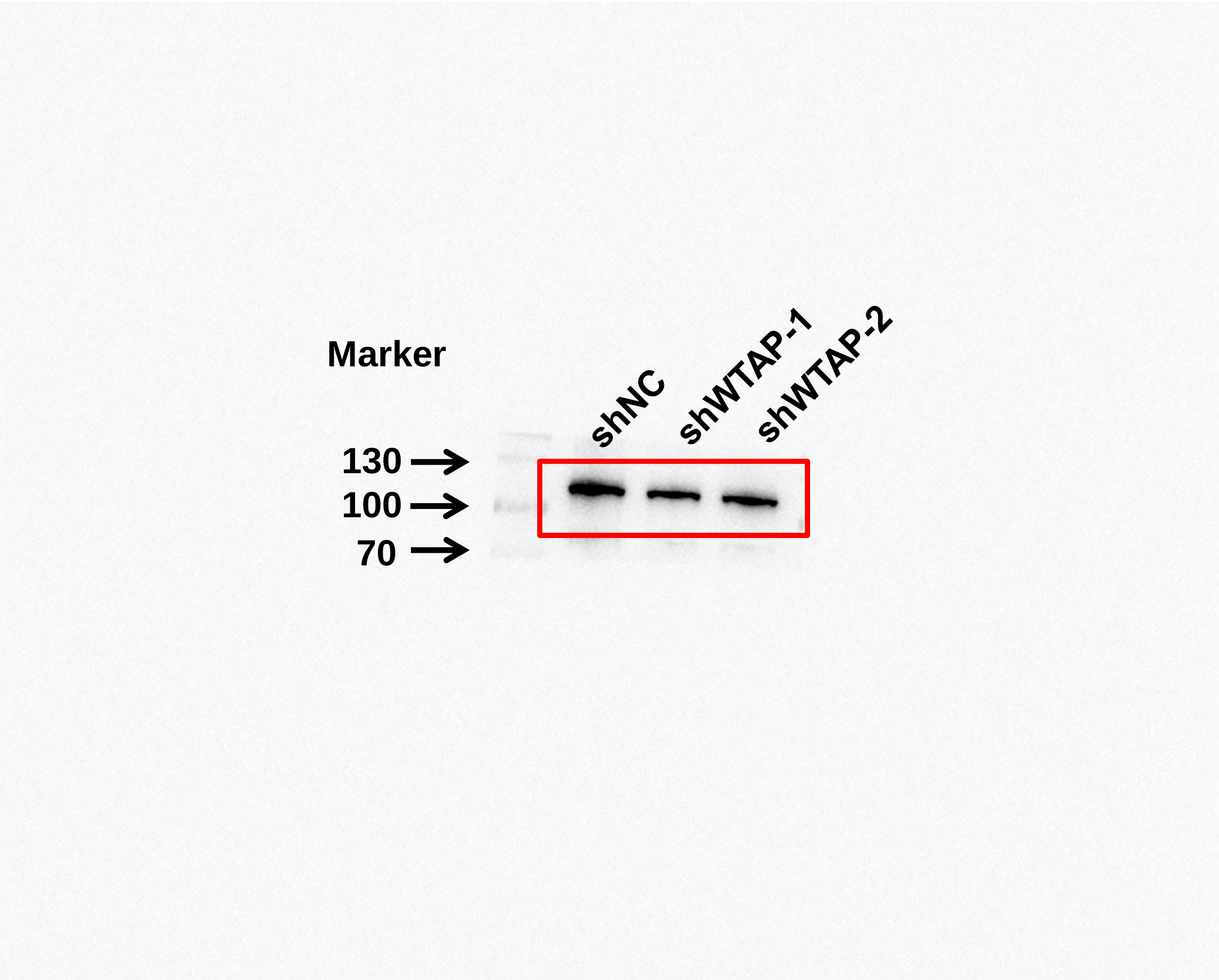

After the membrane was transferred, the membrane was cut from 70-100 kd, and the part of the membrane on 70-100 kd was incubated with **ACTN4** antibody, and band detection was performed.

Figure 2g

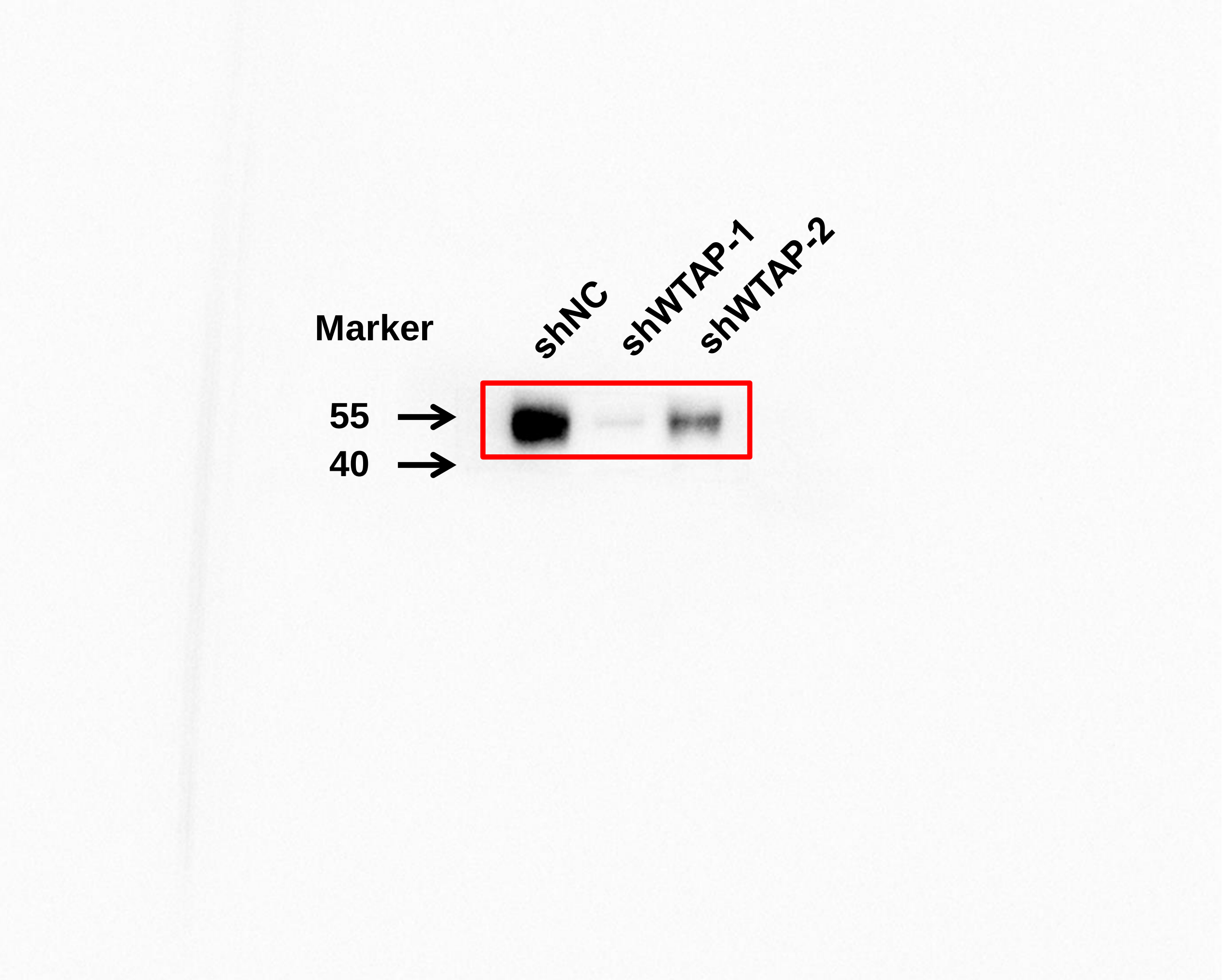

After the membrane was transferred, the membrane was cut from 40-55 kd, and the part of the membrane on 40-55 kd was incubated with **WTAP** antibody, and band detection was performed.

Figure 2g

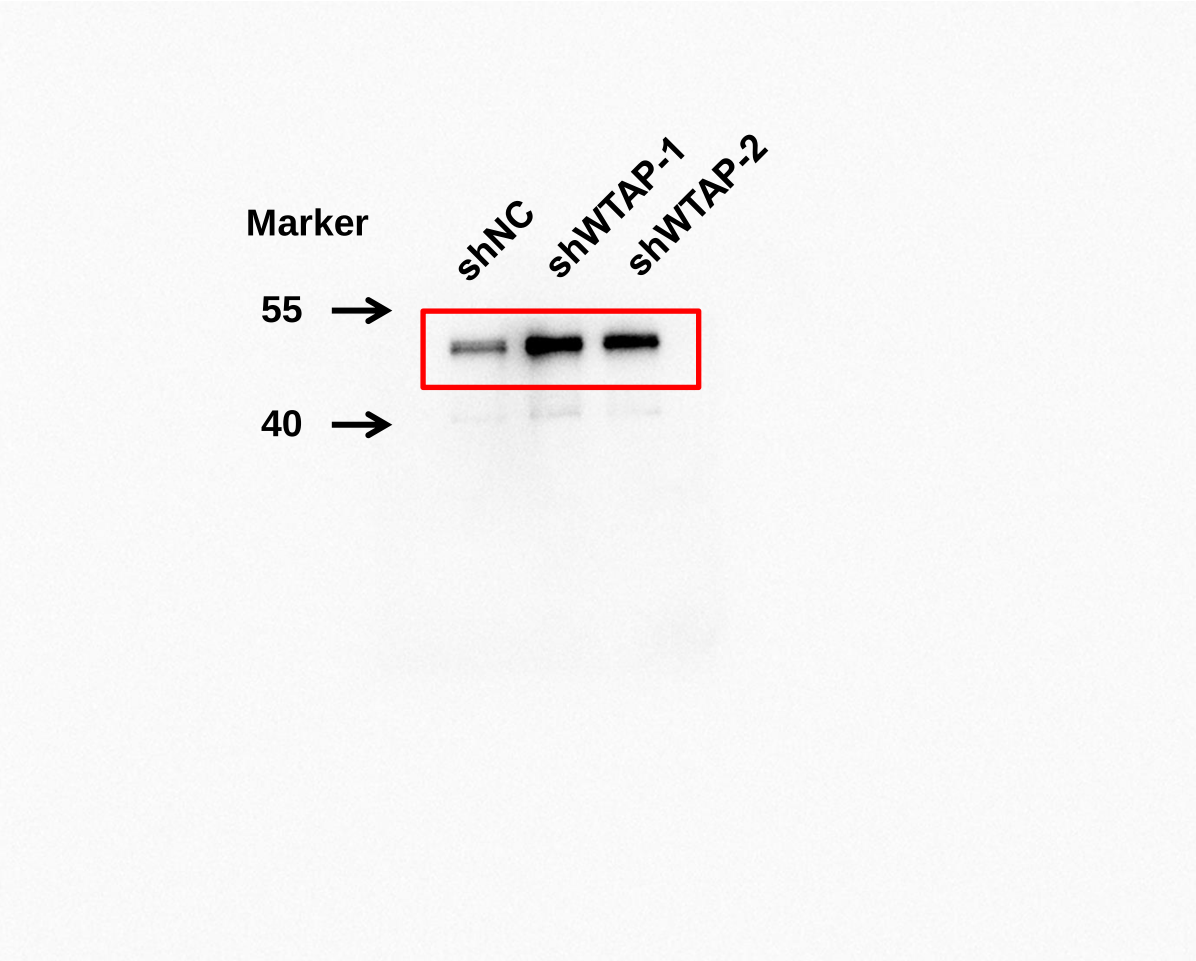

After the membrane was transferred, the membrane was cut from 40-55 kd, and the part of the membrane on 40-55 kd was incubated with **SARS-CoV-2 Nucleoprotein Rabbit pAb** antibody, and band detection was performed.

Figure 2g

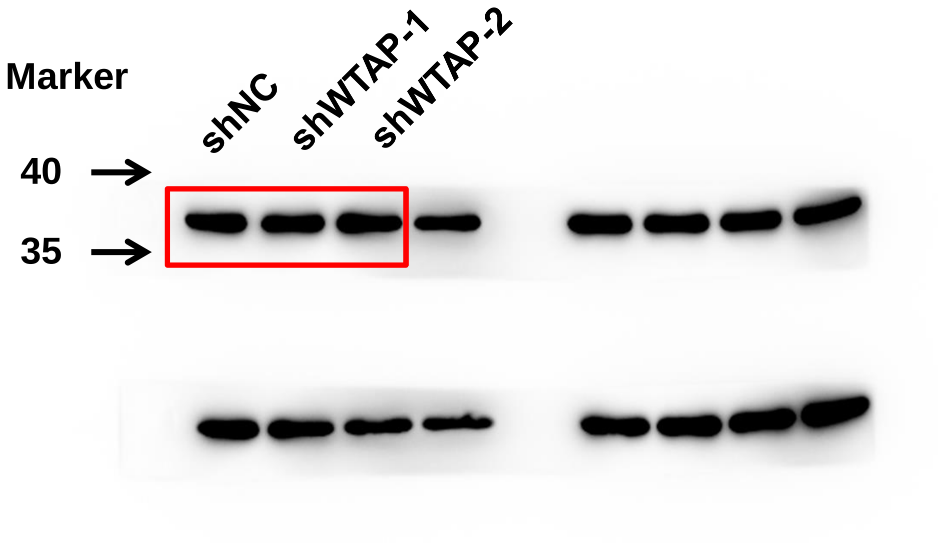

After the membrane was transferred, the membrane was cut from 35-40 kd, and the part of the membrane on 35-40 kd was incubated with **GAPDH** antibody, and band detection was performed.

Figure 3a

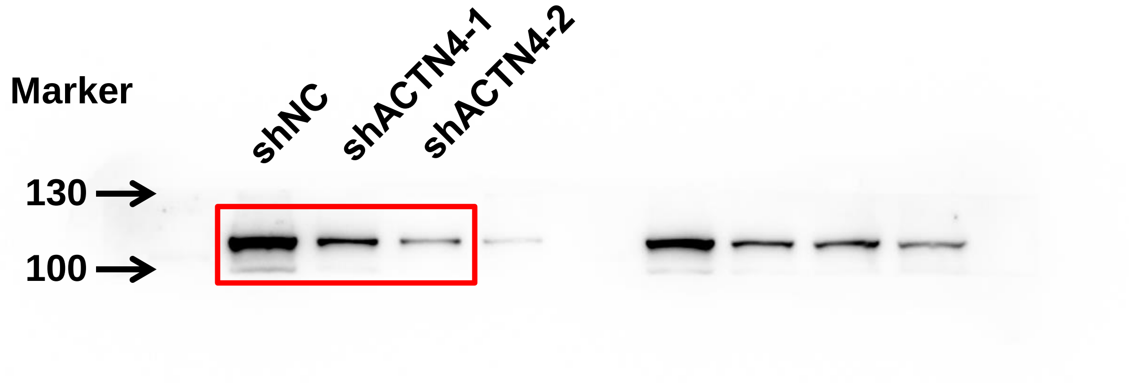

After the membrane was transferred, the membrane was cut from 100-130 kd, and the part of the membrane on 100-130 kd was incubated with **ACTN4** antibody, and band detection was performed.

Figure 3a

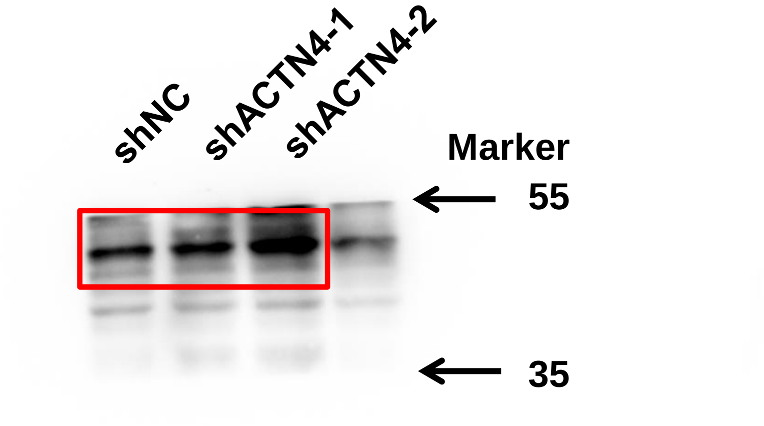

After the membrane was transferred, the membrane was cut from 35-55 kd, and the part of the membrane on 35-55 kd was incubated with **SARS-CoV-2 Nucleoprotein Rabbit pAb** antibody, and band detection was performed.

Figure 3a

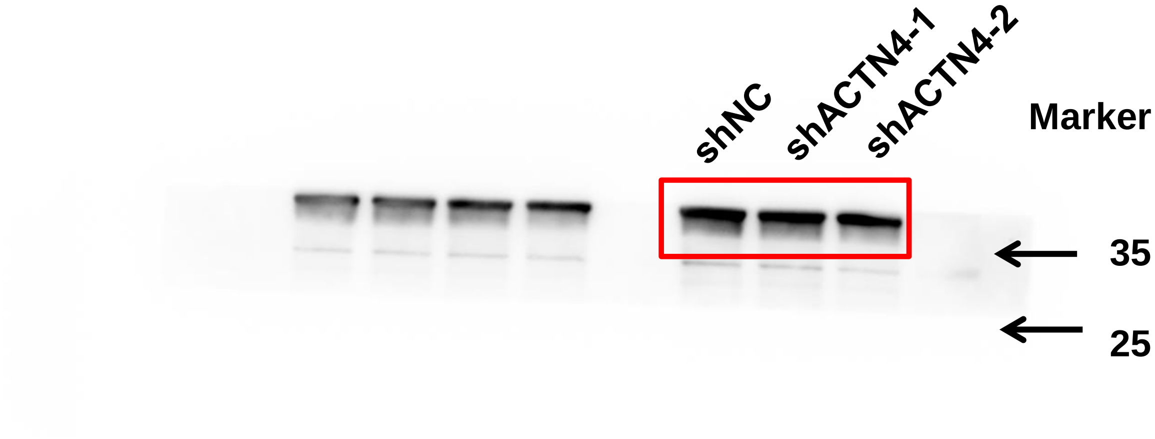

After the membrane was transferred, the membrane was cut from 25-35 kd, and the part of the membrane on 25-35 kd was incubated with **GAPDH** antibody, and band detection was performed.

Figure 3a

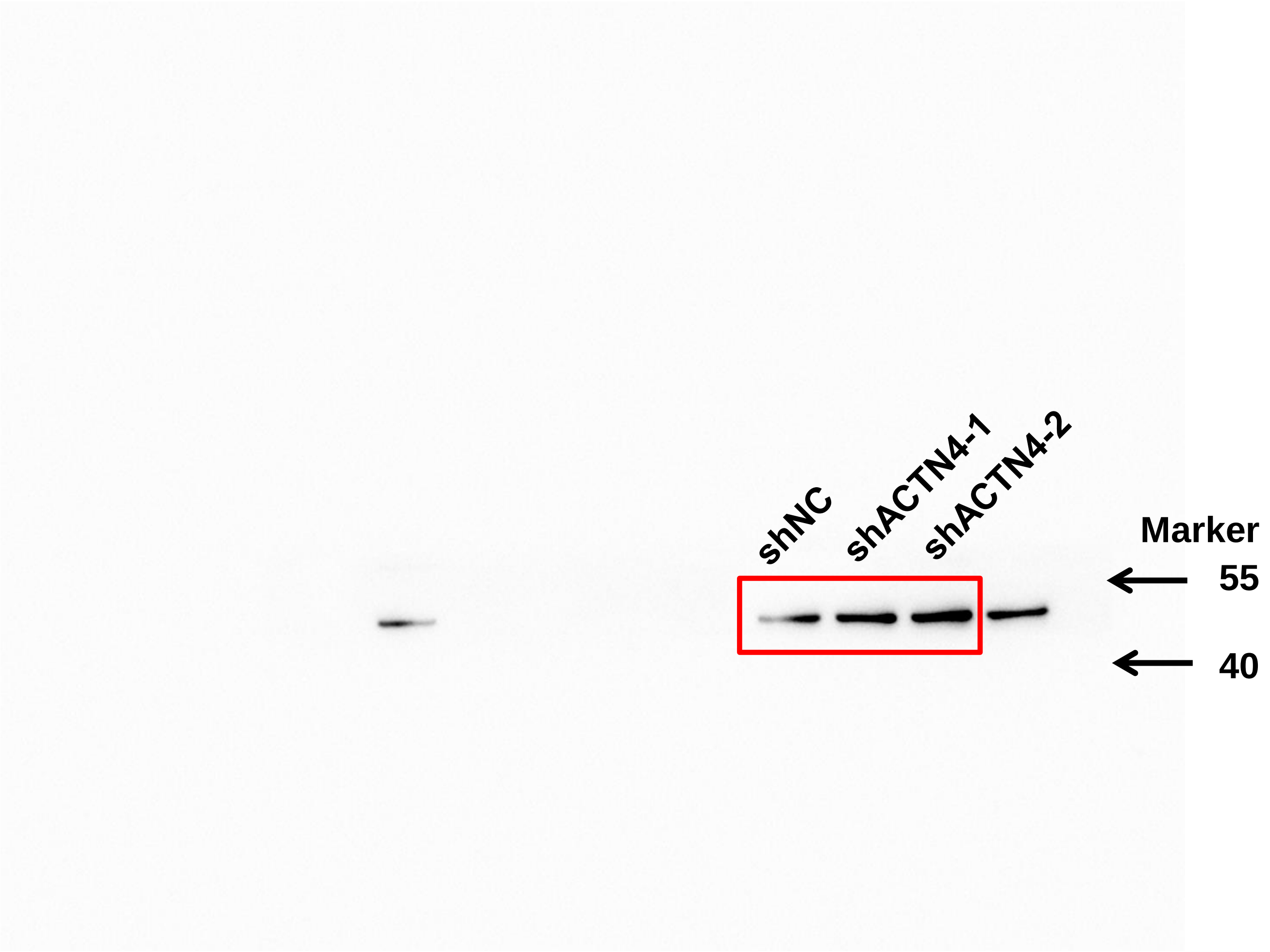

After the membrane was transferred, the membrane was cut from 40-55 kd, and the part of the membrane on 40-55 kd was incubated with **SARS-CoV-2 Nucleoprotein Rabbit pAb** antibody, and band detection was performed.

Figure 3a

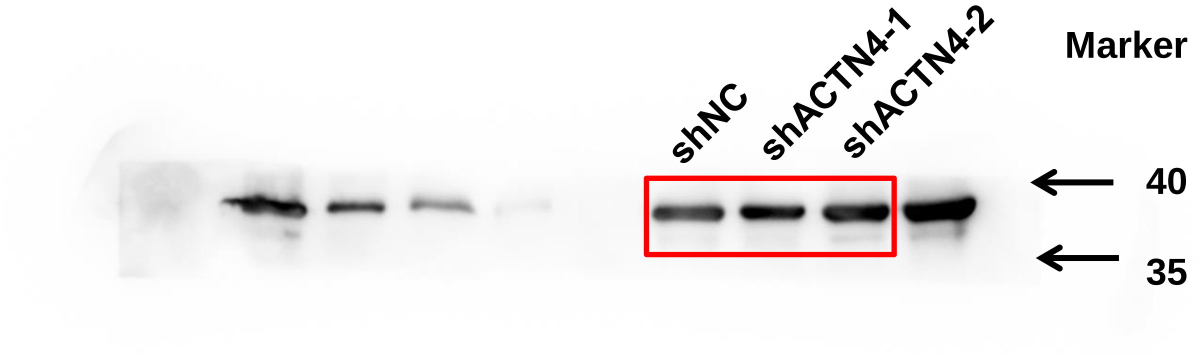

After the membrane was transferred, the membrane was cut from 35-40kd, and the part of the membrane on 35-40 kd was incubated with **GAPDH** antibody, and band detection was performed.

Figure 3d

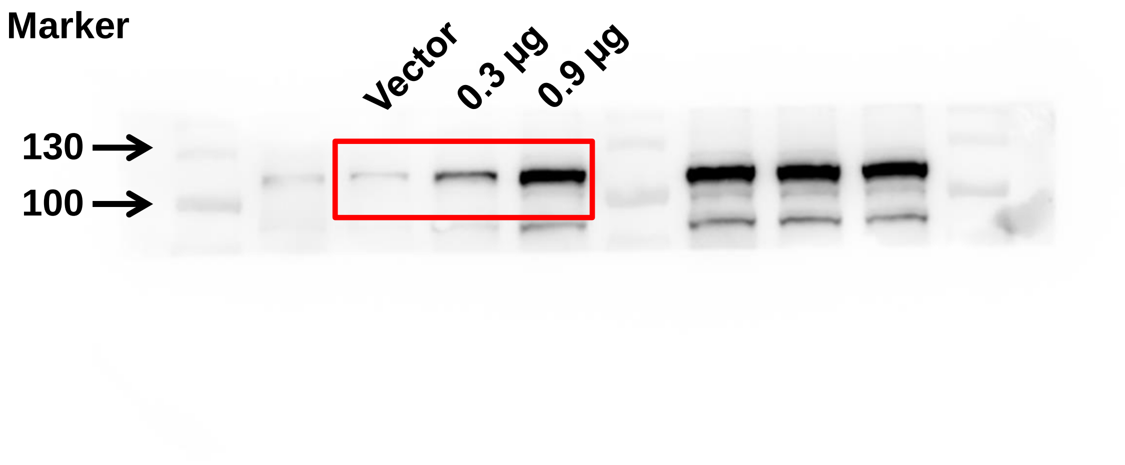

After the membrane was transferred, the membrane was cut from 100-130 kd, and the part of the membrane on 100-130 kd was incubated with **ACTN4** antibody, and band detection was performed.

Figure 3d

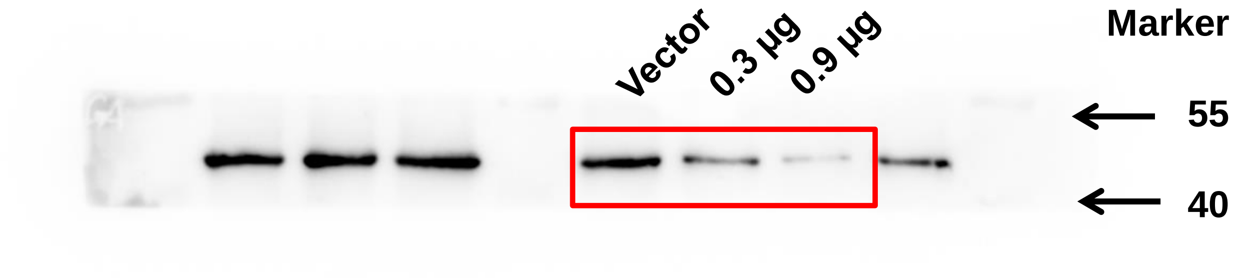

After the membrane was transferred, the membrane was cut from 40-55 kd, and the part of the membrane on 40-55 kd was incubated with **SARS-CoV-2 Nucleoprotein Rabbit pAb** antibody, and band detection was performed.

Figure 3d

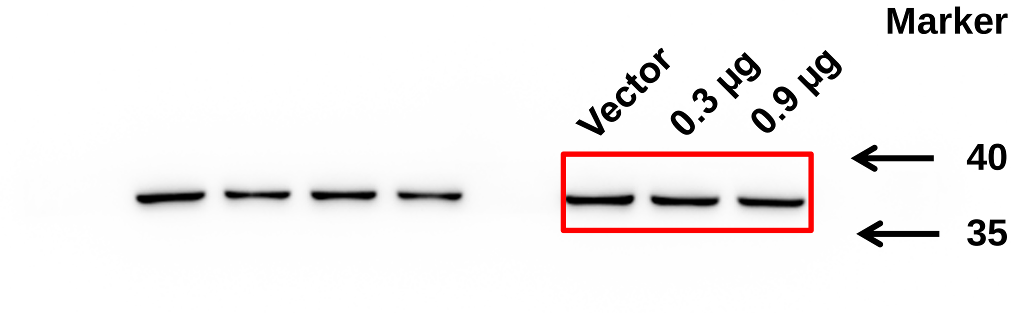

After the membrane was transferred, the membrane was cut from 35-40kd, and the part of the membrane on 35-40 kd was incubated with **GAPDH** antibody, and band detection was performed.

Figure 3d

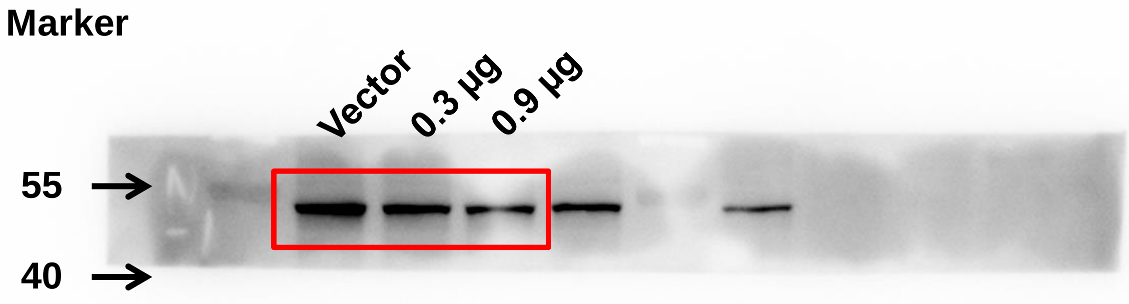

After the membrane was transferred, the membrane was cut from 40-55 kd, and the part of the membrane on 40-55 kd was incubated with **SARS-CoV-2 Nucleoprotein Rabbit pAb** antibody, and band detection was performed.

Figure 3d

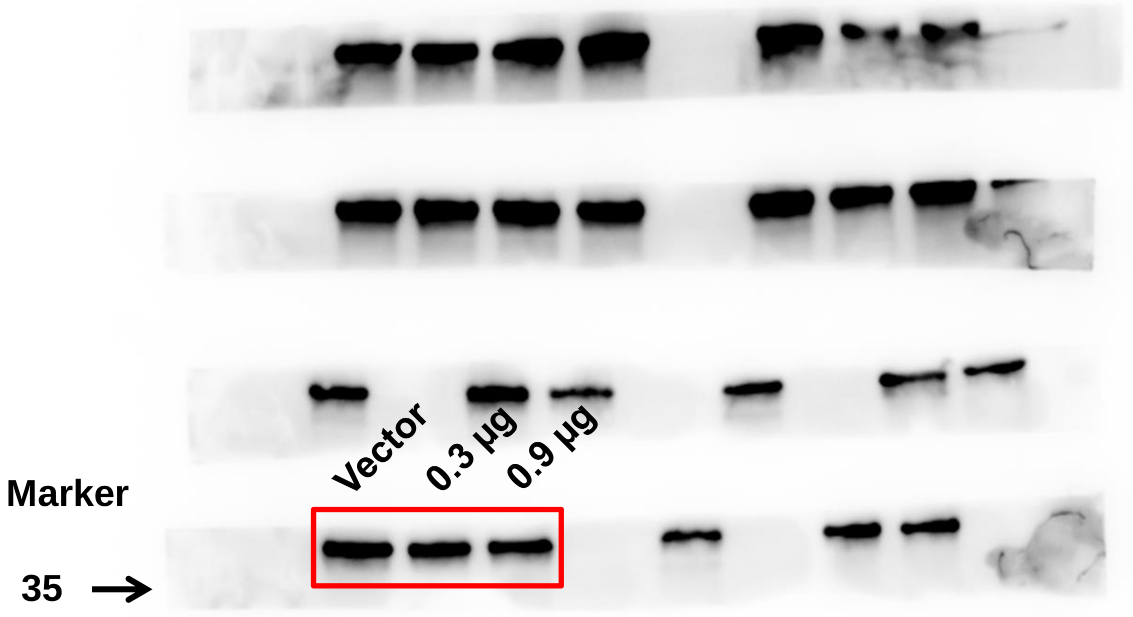

After the membrane was transferred, the membrane was cut from 35-40kd, and the part of the membrane on 35-40 kd was incubated with **GAPDH** antibody, and band detection was performed.

Figure 3g and h

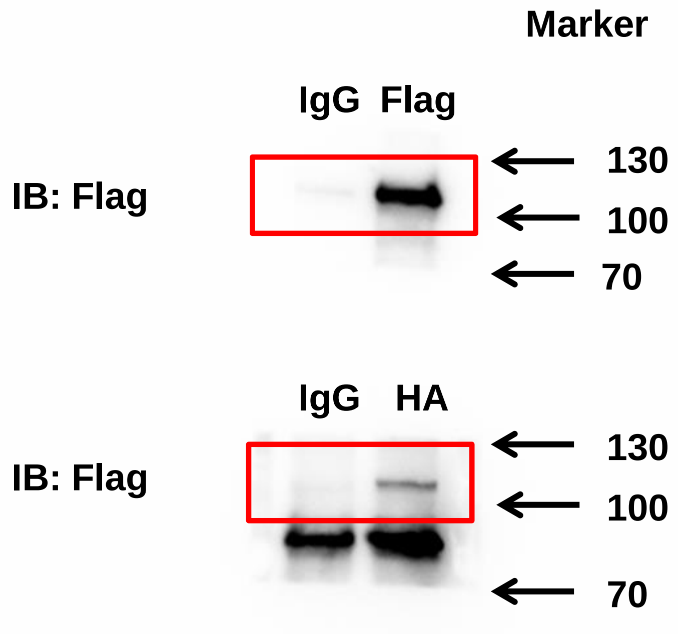

After the membrane was transferred, the membrane was cut from 70-130kd, and the part of the membrane on 70-130 kd was incubated with **Flag** antibody, and band detection was performed.

Figure 3g and h

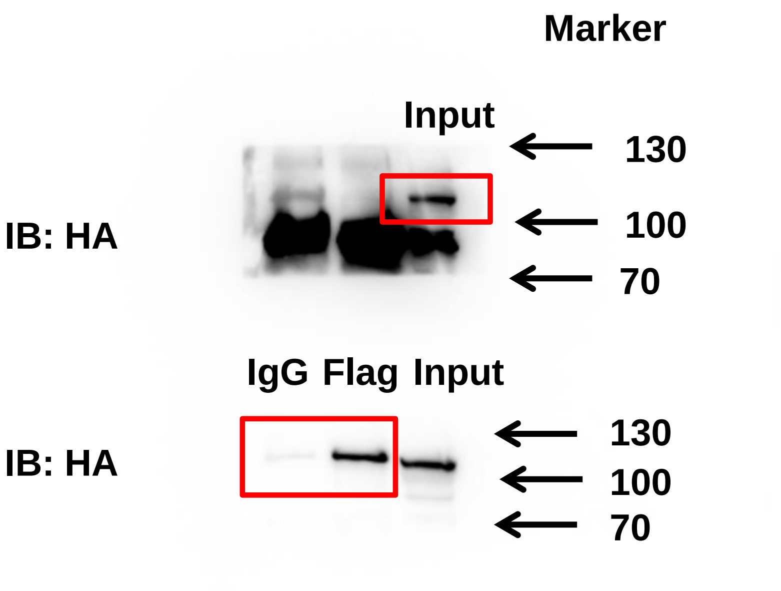

After the membrane was transferred, the membrane was cut from 70-130kd, and the part of the membrane on 70-130 kd was incubated with **HA** antibody, and band detection was performed.

Figure 3g and h

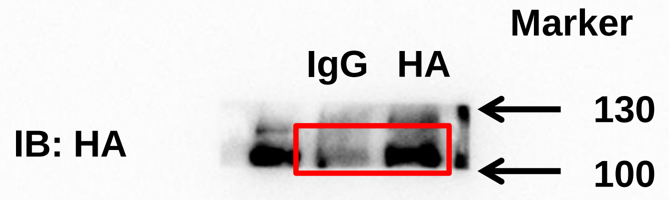

After the membrane was transferred, the membrane was cut from 100-130kd, and the part of the membrane on 100-130 kd was incubated with **HA** antibody, and band detection was performed.

Figure 4b

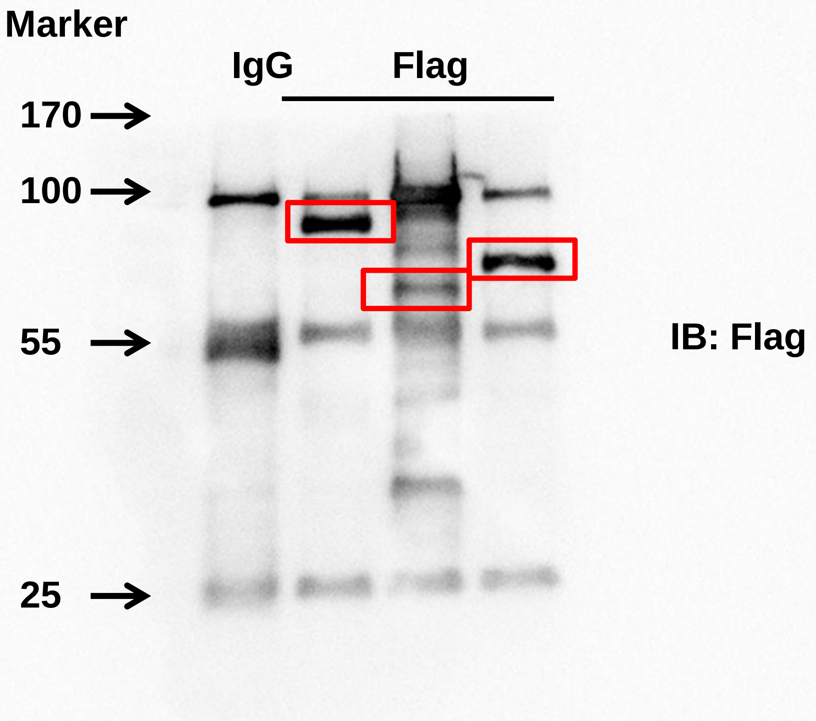

After the membrane was transferred, the membrane was cut from 25-170 kd, and the part of the membrane on 25-170 kd was incubated with **Flag** antibody, and band detection was performed.

Figure 4b

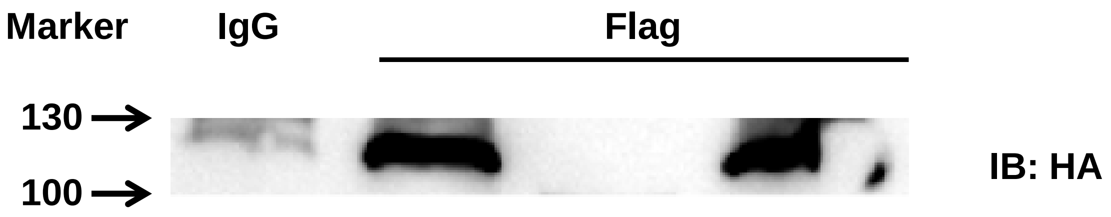

After the membrane was transferred, the membrane was cut from 100-130 kd, and the part of the membrane on 100-130 kd was incubated with **HA** antibody, and band detection was performed.

Figure 4d

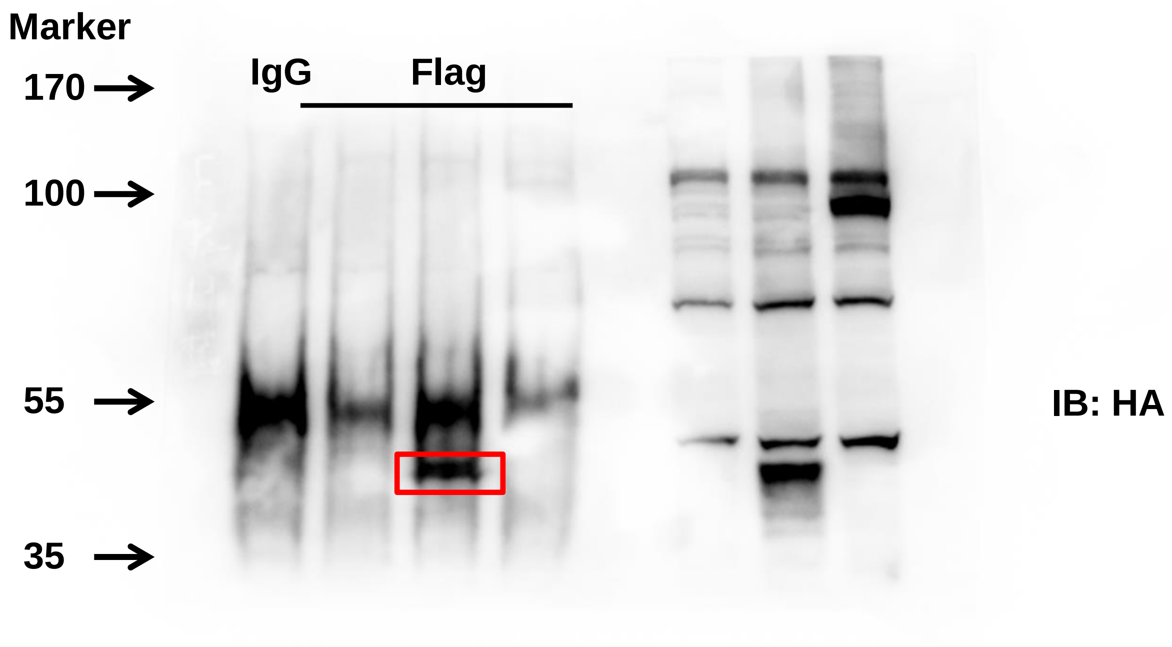

After the membrane was transferred, the membrane was cut from 35-170 kd, and the part of the membrane on 35-170 kd was incubated with **HA** antibody, and band detection was performed.

Figure 4d

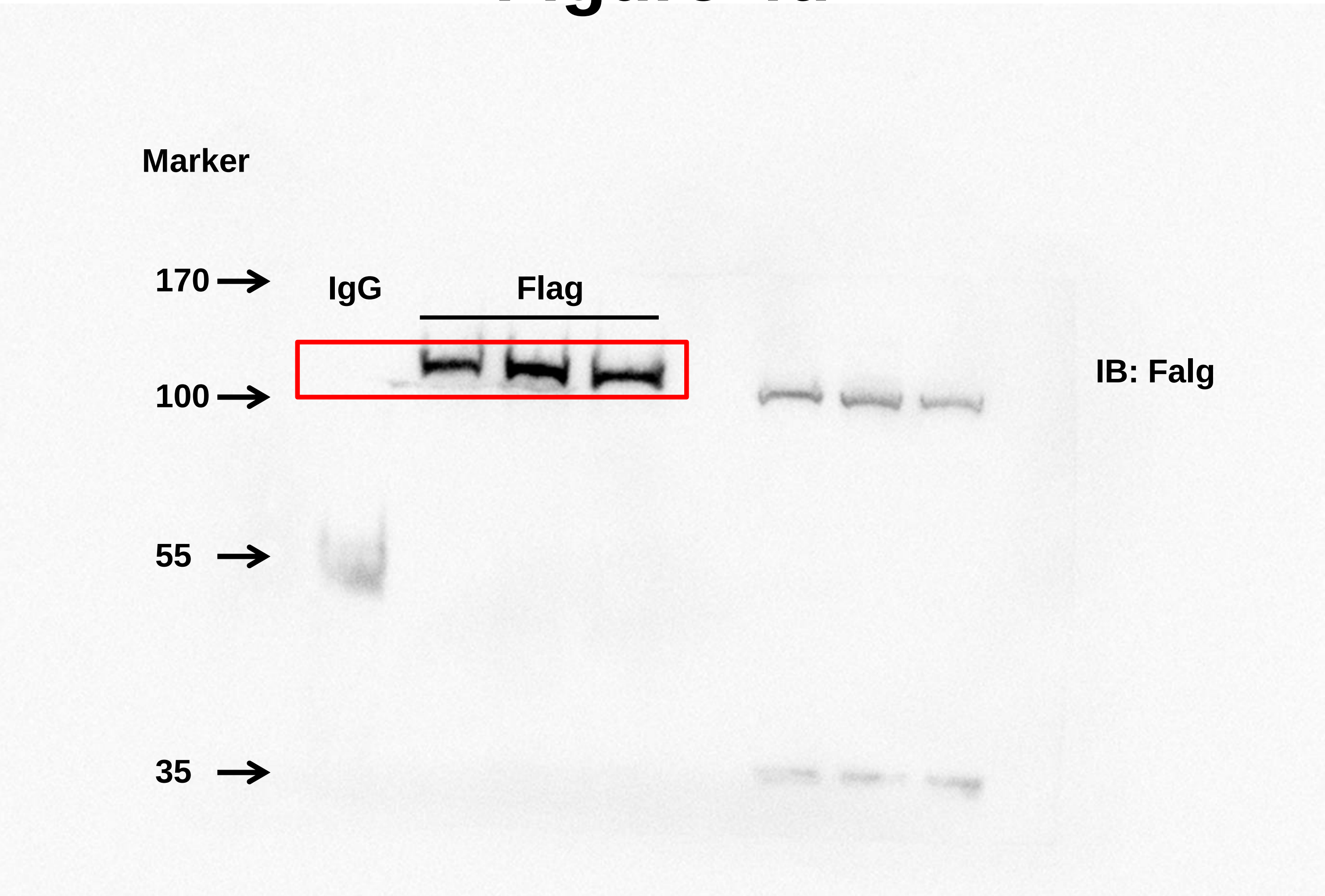

After the membrane was transferred, the membrane was cut from 35-170 kd, and the part of the membrane on 35-170 kd was incubated with **Flag** antibody, and band detection was performed.

Figure 4e

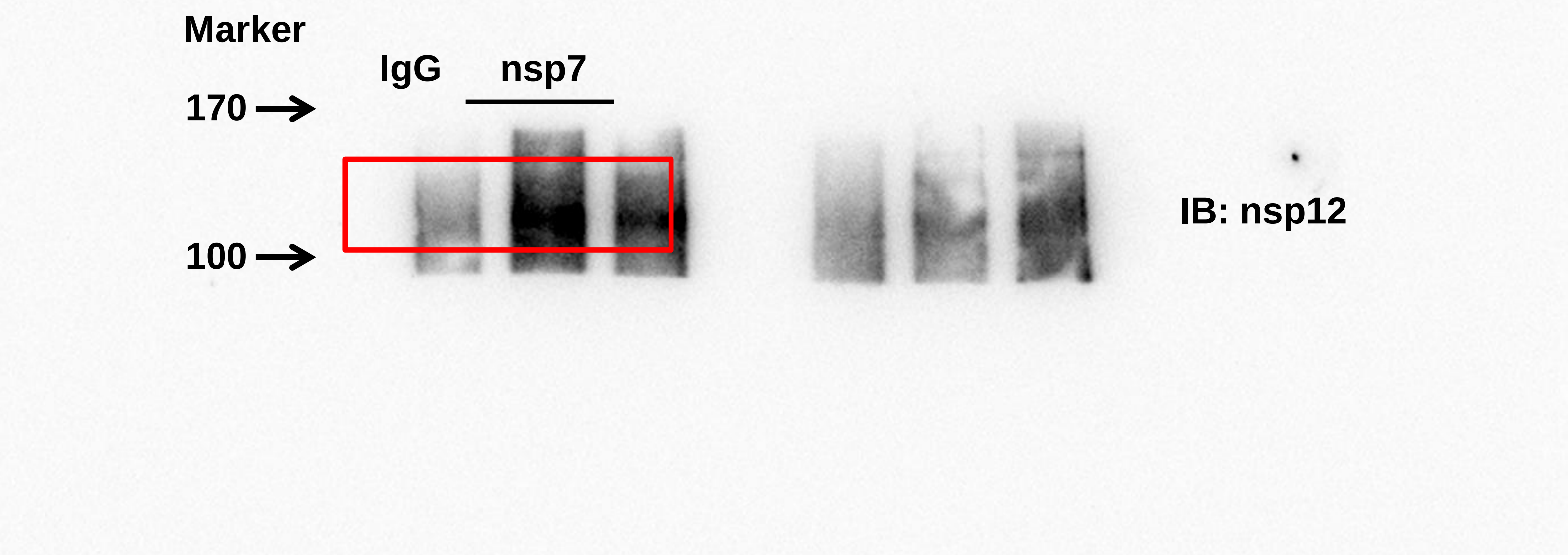

After the membrane was transferred, the membrane was cut from 100-170 kd, and the part of the membrane on 100-170 kd was incubated with **nsp12** antibody, and band detection was performed.

Figure 4e

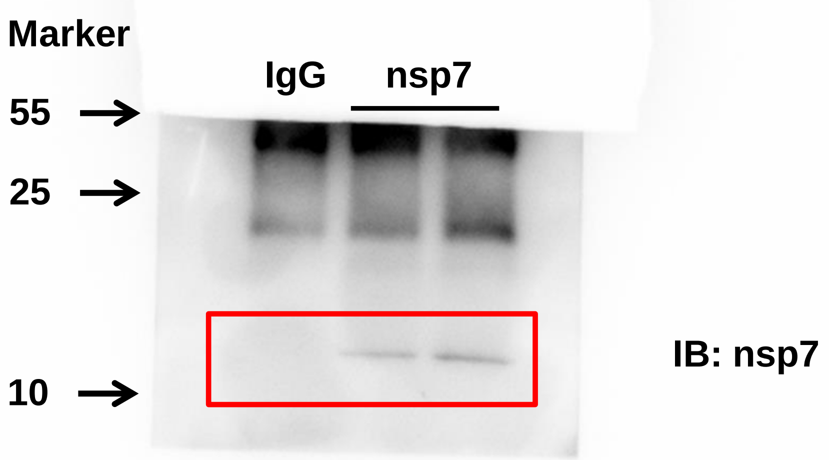

After the membrane was transferred, the membrane was cut from 10-55 kd, and the part of the membrane on 10-25 kd was incubated with **nsp7** antibody, and band detection was performed.

Figure 4e

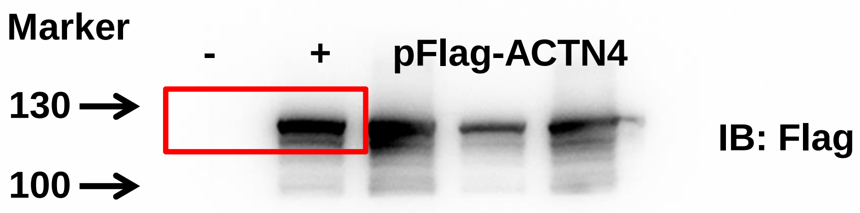

After the membrane was transferred, the membrane was cut from 100-130 kd, and the part of the membrane on 100-130 kd was incubated with **Flag** antibody, and band detection was performed.

Figure 4e

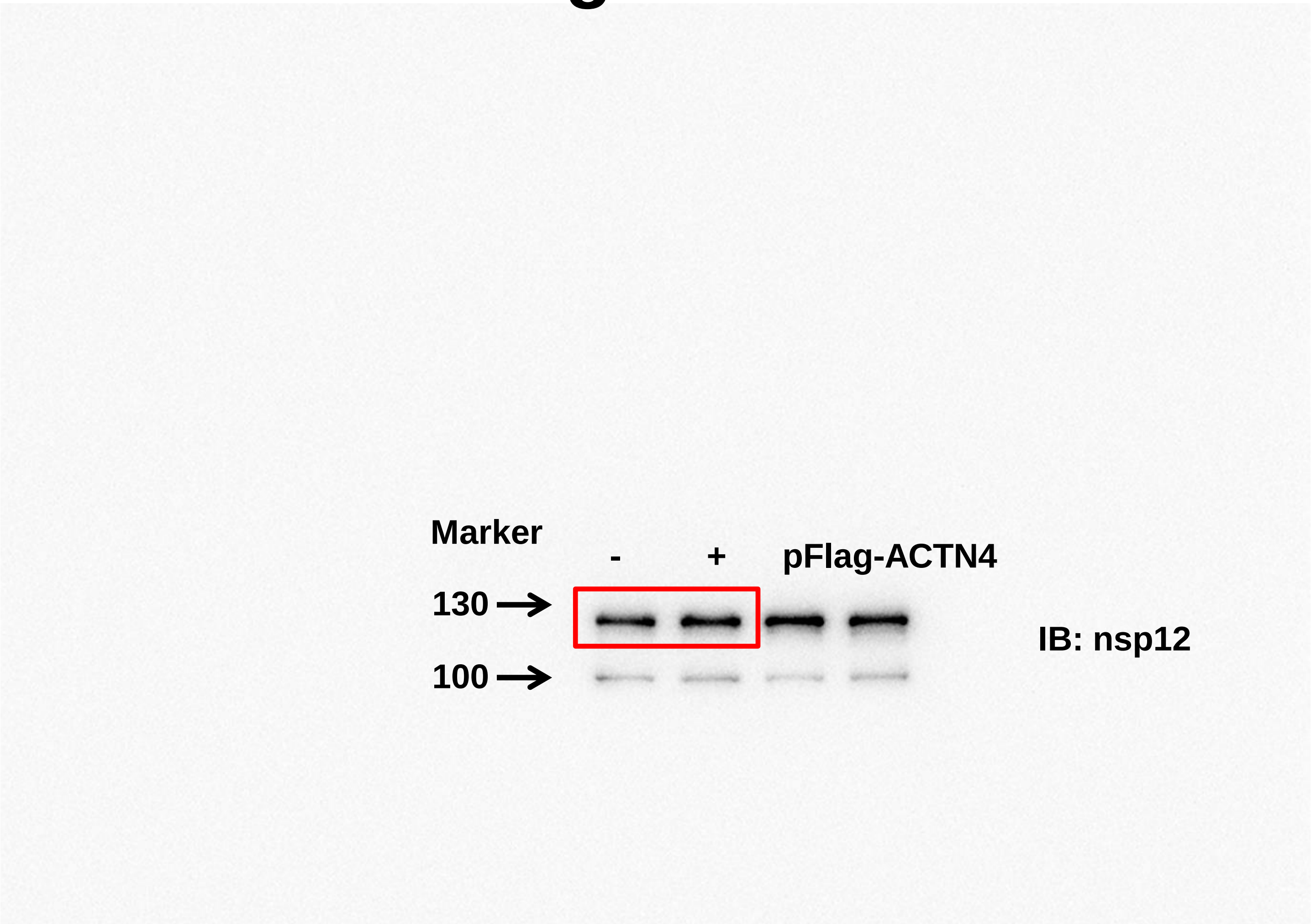

After the membrane was transferred, the membrane was cut from 100-130 kd, and the part of the membrane on 100-130 kd was incubated with **nsp12** antibody, and band detection was performed.

Figure 4e

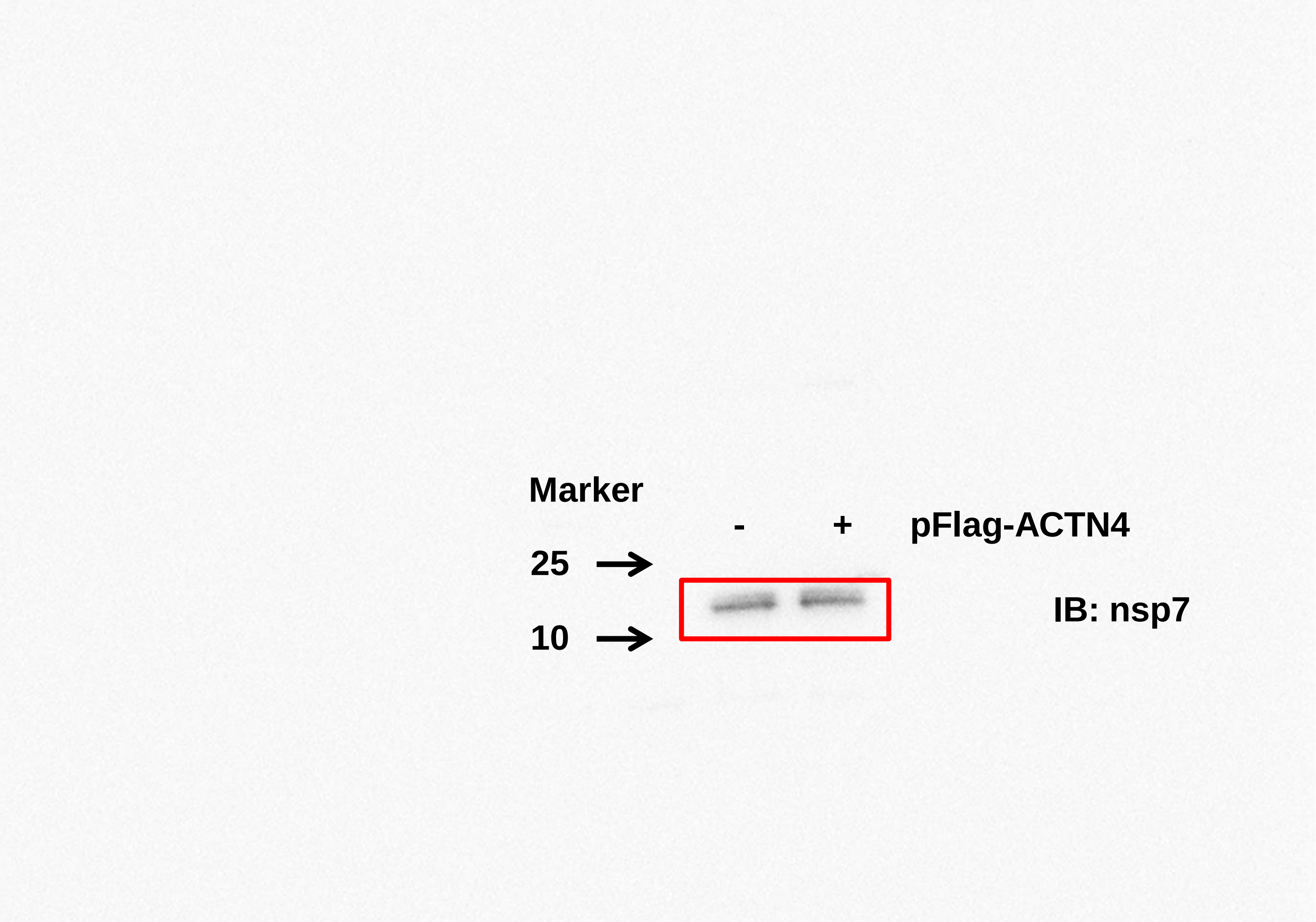

After the membrane was transferred, the membrane was cut from 10-25 kd, and the part of the membrane on 10-25 kd was incubated with **nsp7** antibody, and band detection was performed.

Figure 4f

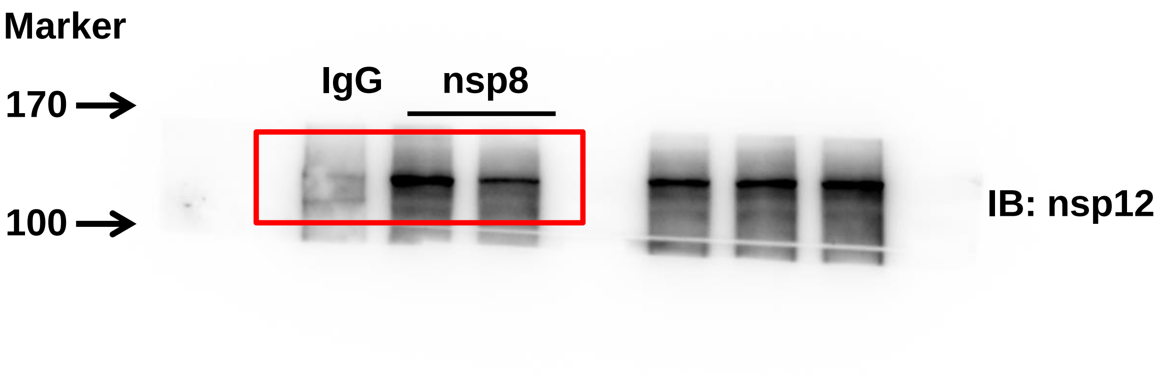

After the membrane was transferred, the membrane was cut from 100-170 kd, and the part of the membrane on 100-170 kd was incubated with **nsp12** antibody, and band detection was performed.

Figure 4f

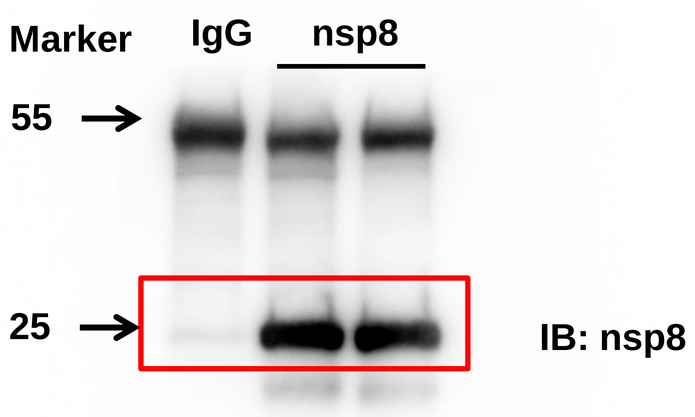

After the membrane was transferred, the membrane was cut from 10-55 kd, and the part of the membrane on 10-55 kd was incubated with **nsp8** antibody, and band detection was performed.

Figure 4f

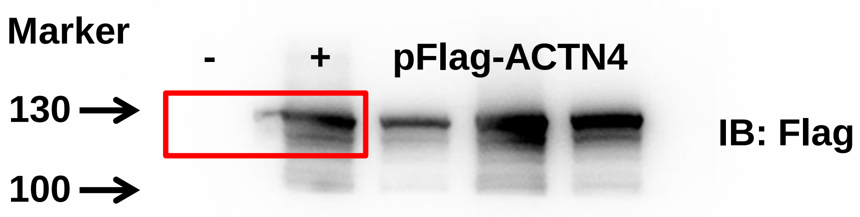

After the membrane was transferred, the membrane was cut from 100-130 kd, and the part of the membrane on 100-130 kd was incubated with **Flag** antibody, and band detection was performed.

Figure 4f

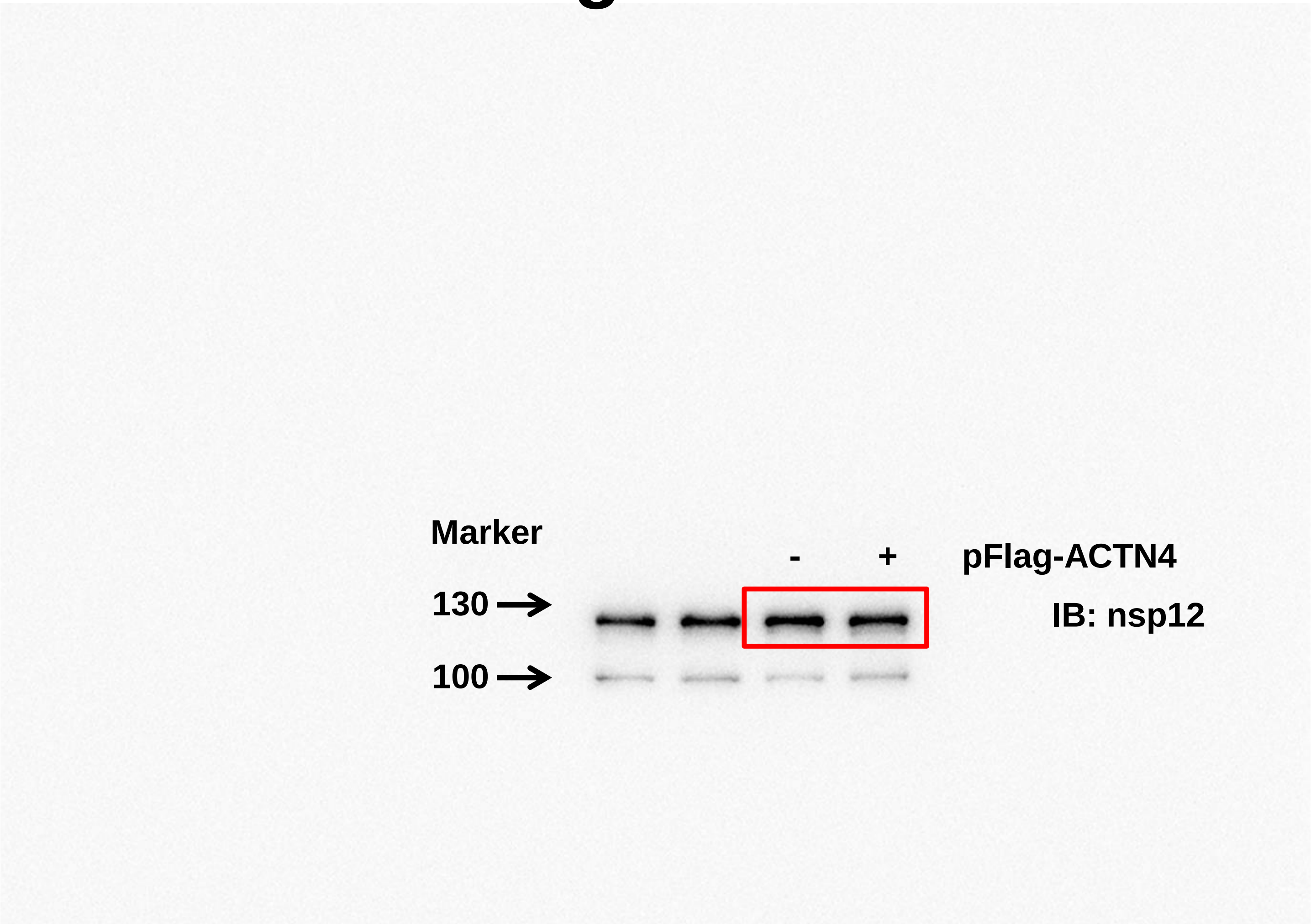

After the membrane was transferred, the membrane was cut from 100-130 kd, and the part of the membrane on 100-130 kd was incubated with **nsp12** antibody, and band detection was performed.

Figure 4f

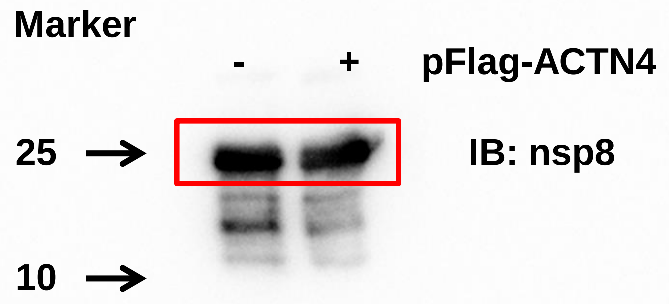

After the membrane was transferred, the membrane was cut from 10-25 kd, and the part of the membrane on 10-25 kd was incubated with **nsp8** antibody, and band detection was performed.

Figure 5d

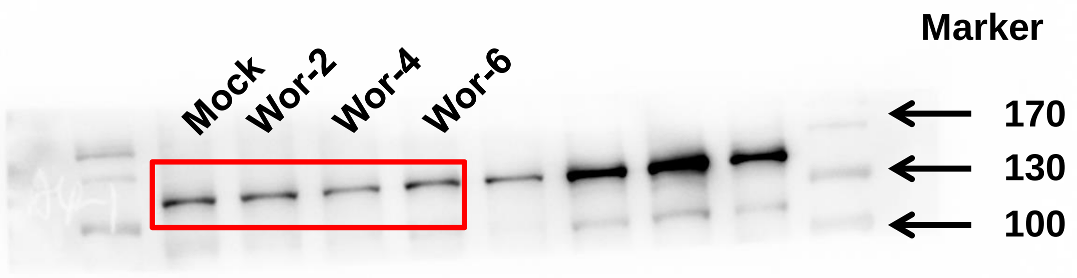

After the membrane was transferred, the membrane was cut from 100-170kd, and the part of the membrane on 100-170 kd was incubated with **ACTN4** antibody, and band detection was performed.

Figure 5d

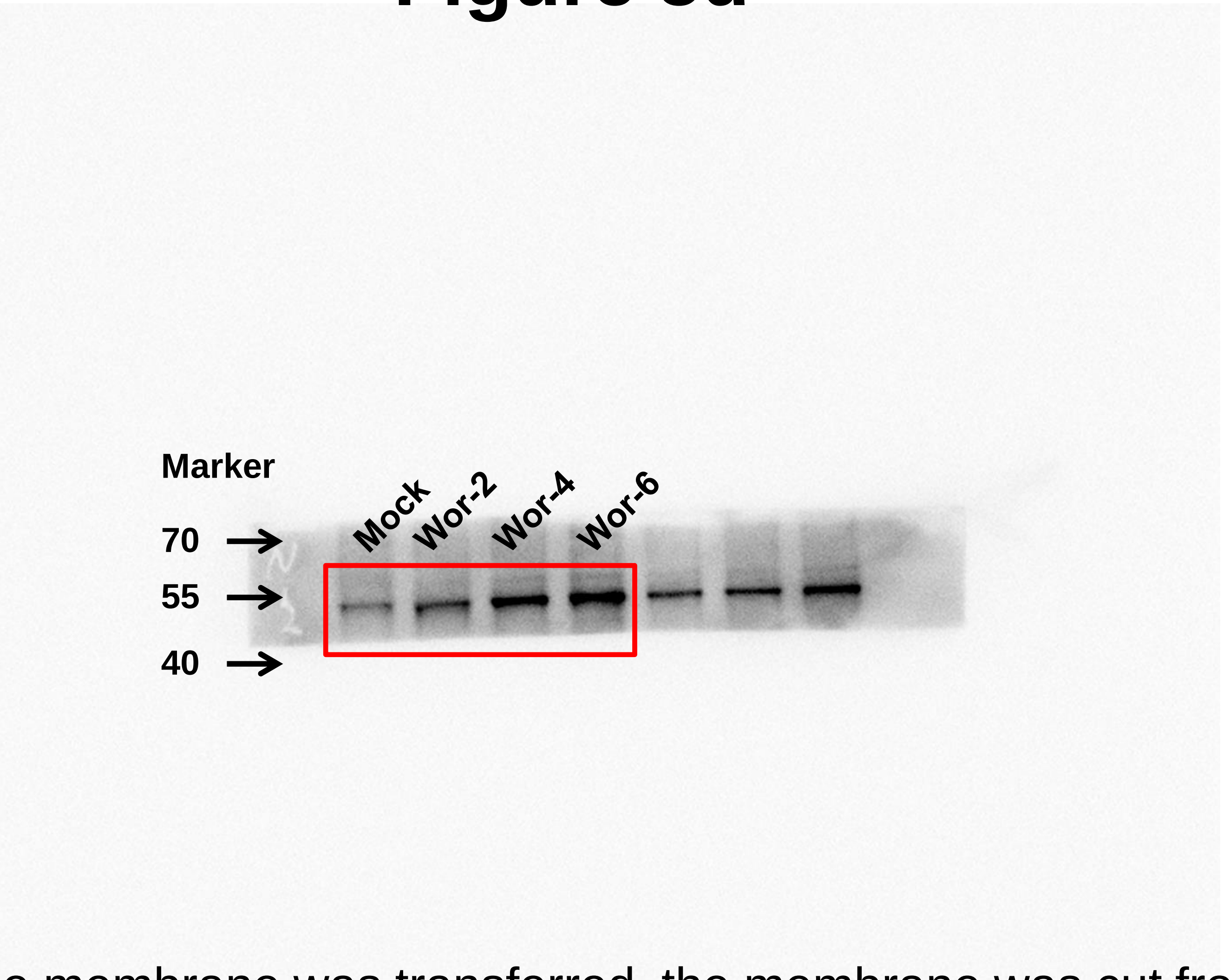

After the membrane was transferred, the membrane was cut from 40-70kd, and the part of the membrane on 40-70 kd was incubated with **SARS-CoV-2 Nucleoprotein Rabbit pAb** antibody, and band detection was performed.

Figure 5d

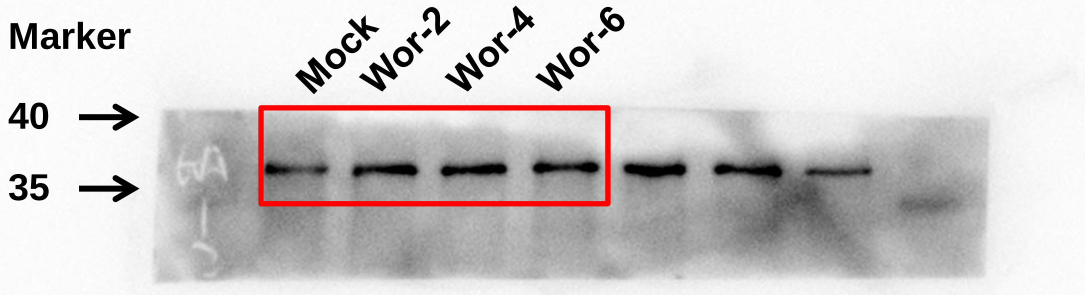

After the membrane was transferred, the membrane was cut from 35-40kd, and the part of the membrane on 35-40 kd was incubated with **GAPDH** antibody, and band detection was performed.

Figure 5h

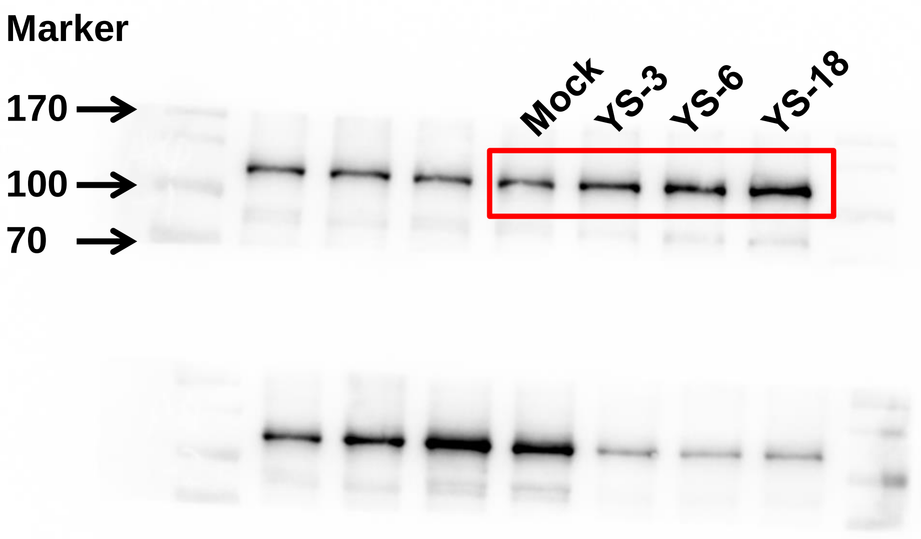

After the membrane was transferred, the membrane was cut from 70-170kd, and the part of the membrane on 70-170 kd was incubated with **ACTN4** antibody, and band detection was performed.

Figure 5h

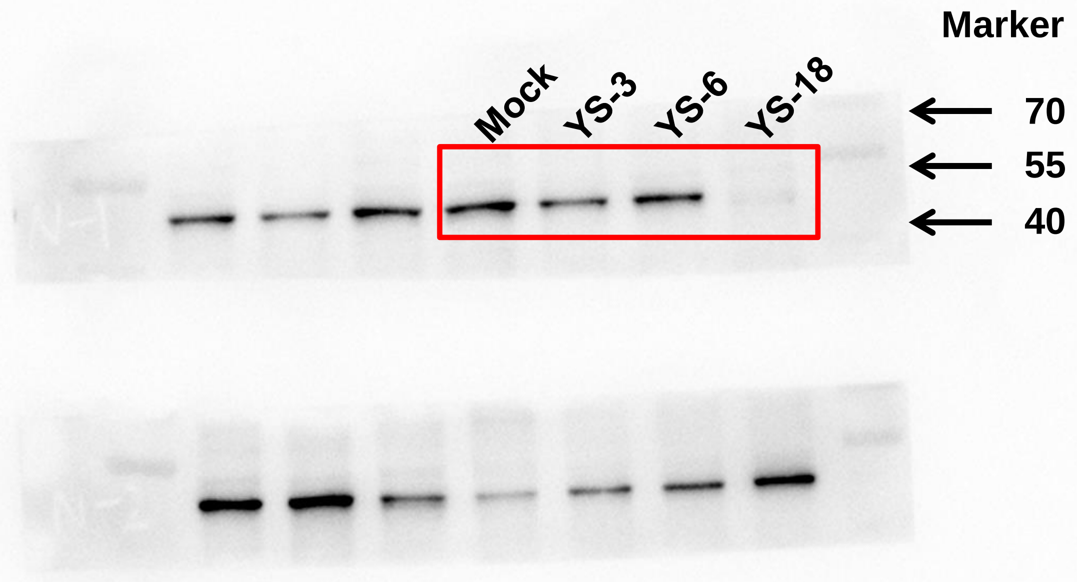

After the membrane was transferred, the membrane was cut from 40-70kd, and the part of the membrane on 40-70 kd was incubated with **SARS-CoV-2 Nucleoprotein Rabbit pAb** antibody, and band detection was performed.

Figure 5h

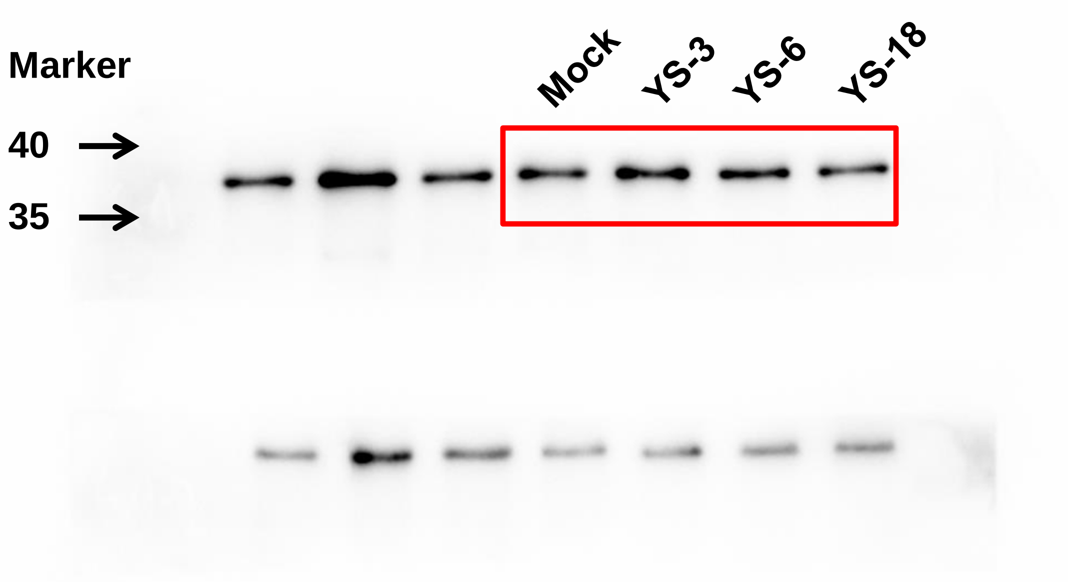

After the membrane was transferred, the membrane was cut from 35-40kd, and the part of the membrane on 35-40 kd was incubated with **GAPDH** antibody, and band detection was performed.

Figure 5l

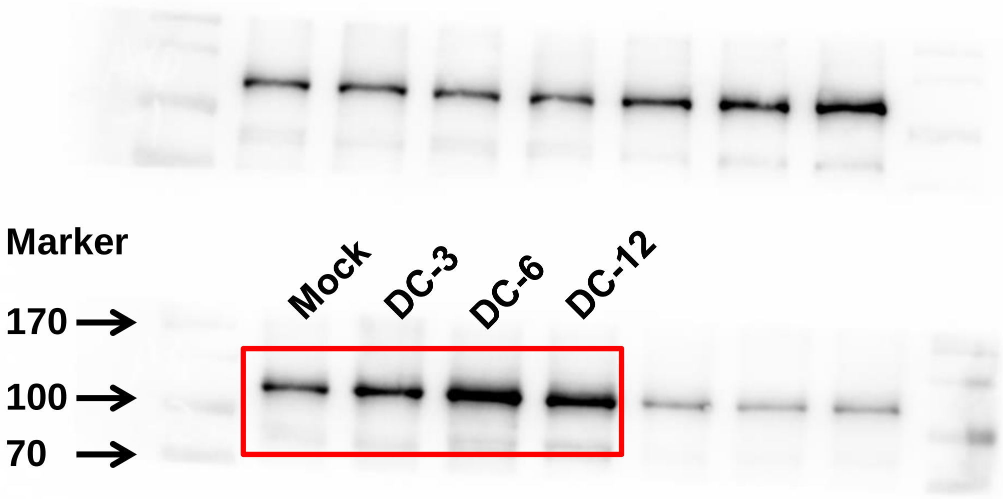

After the membrane was transferred, the membrane was cut from 70-170kd, and the part of the membrane on 70-170 kd was incubated with **ACTN4** antibody, and band detection was performed.

Figure 5I

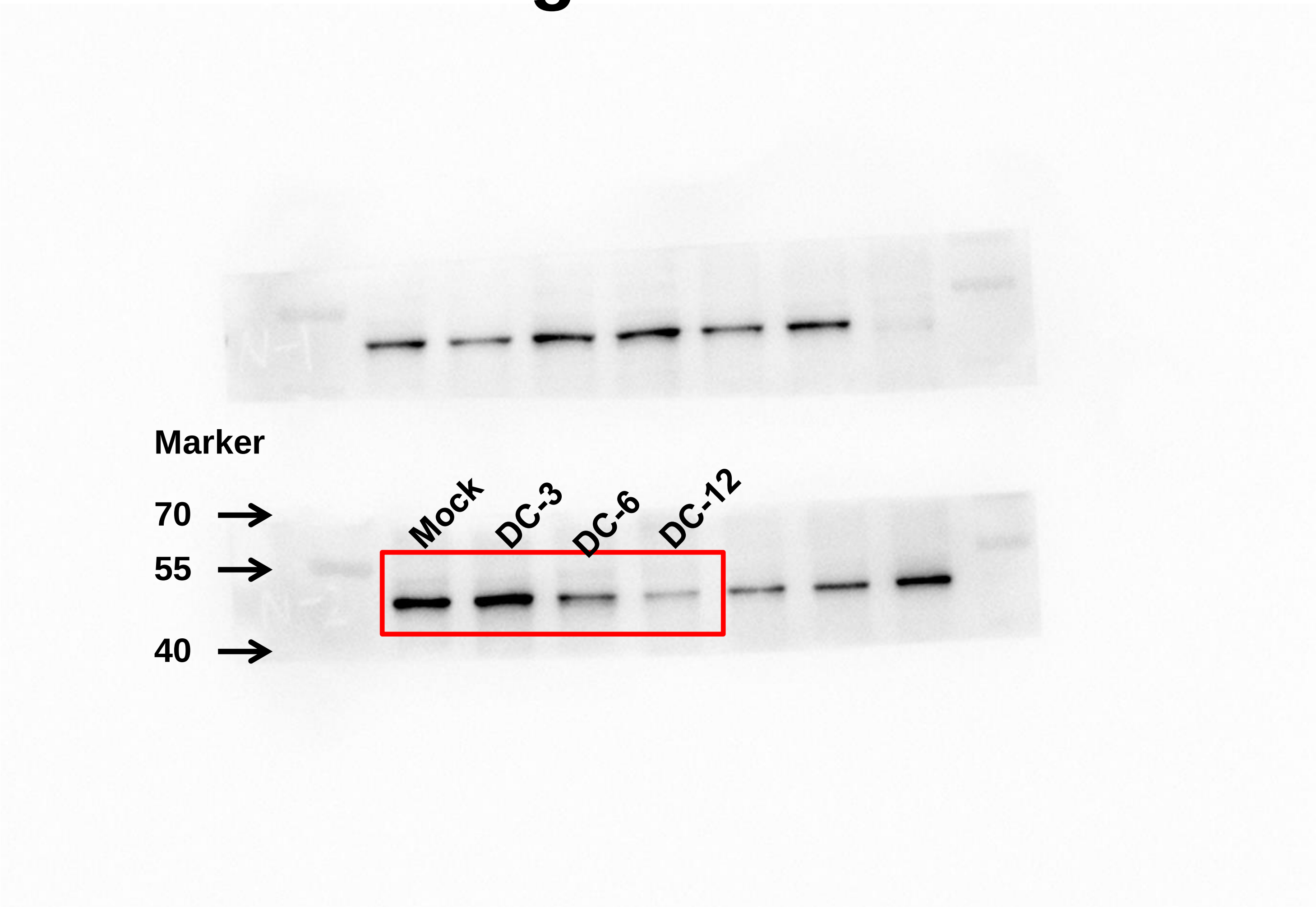

After the membrane was transferred, the membrane was cut from 40-70kd, and the part of the membrane on 40-70 kd was incubated with **SARS-CoV-2 Nucleoprotein Rabbit pAb** antibody, and band detection was performed.

Figure 5l

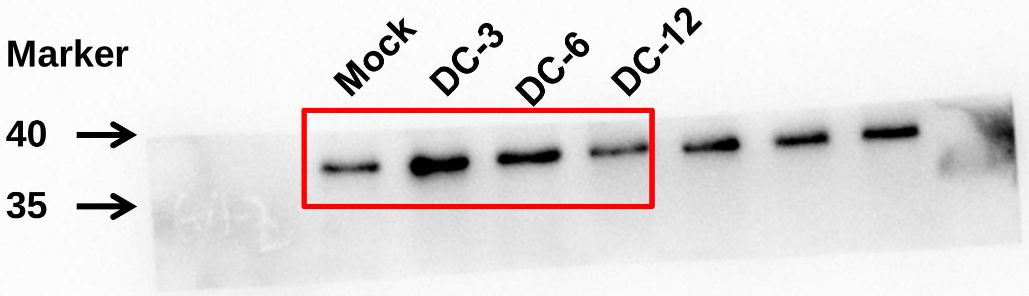

After the membrane was transferred, the membrane was cut from 35-40kd, and the part of the membrane on 35-40 kd was incubated with **GAPDH** antibody, and band detection was performed.

Figure S1e

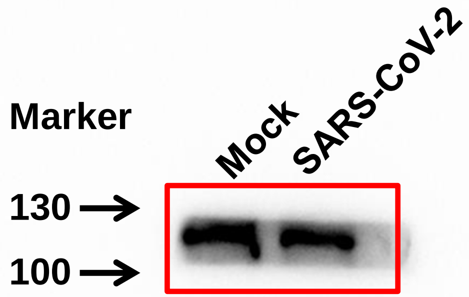

After the membrane was transferred, the membrane was cut from 100-130kd, and the part of the membrane on 100-130 kd was incubated with **ACTN4** antibody, and band detection was performed.

Figure S1e

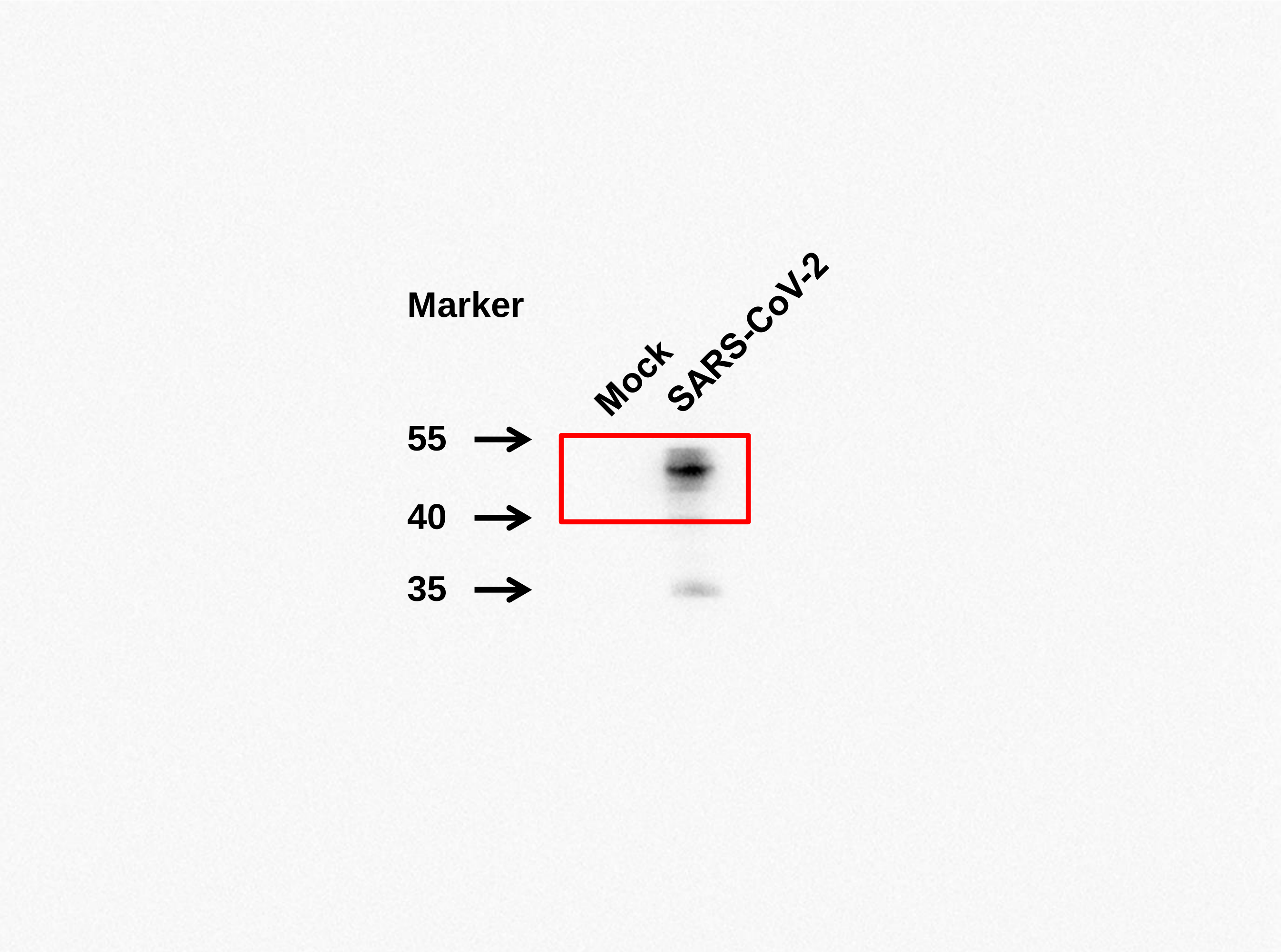

After the membrane was transferred, the membrane was cut from 35-55kd, and the part of the membrane on 35-55 kd was incubated with **SARS-CoV-2 Nucleoprotein Rabbit pAb** antibody, and band detection was performed.

Figure S1e

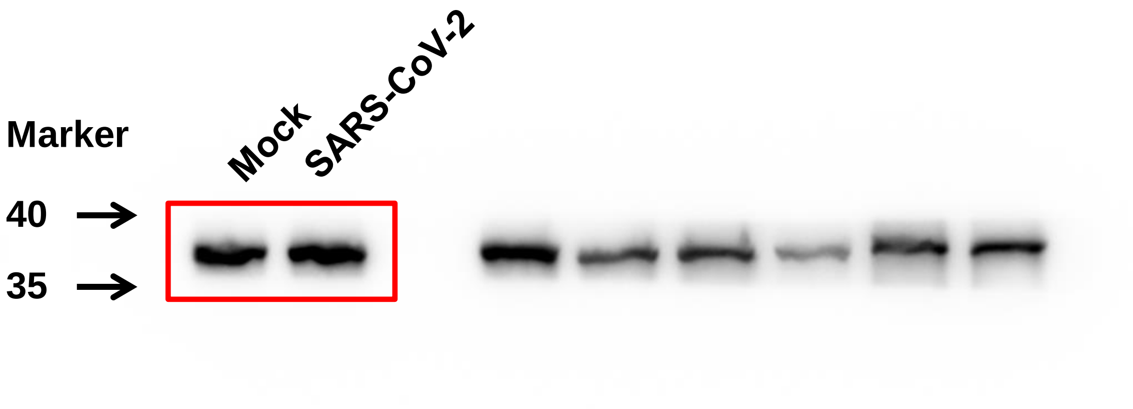

After the membrane was transferred, the membrane was cut from 35-40kd, and the part of the membrane on 35-40 kd was incubated with **GAPDH** antibody, and band detection was performed.

Figure S2c

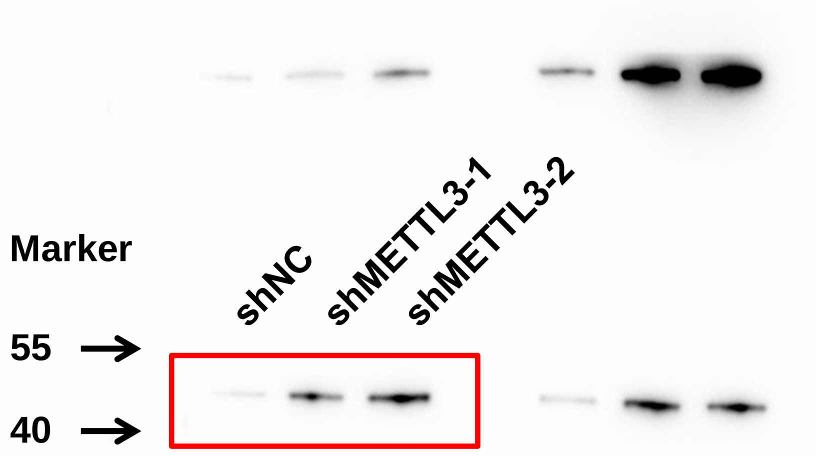

After the membrane was transferred, the membrane was cut from 40-55kd, and the part of the membrane on 40-55 kd was incubated with **SARS-CoV-2 Nucleoprotein Rabbit pAb** antibody, and band detection was performed.

Figure S2c

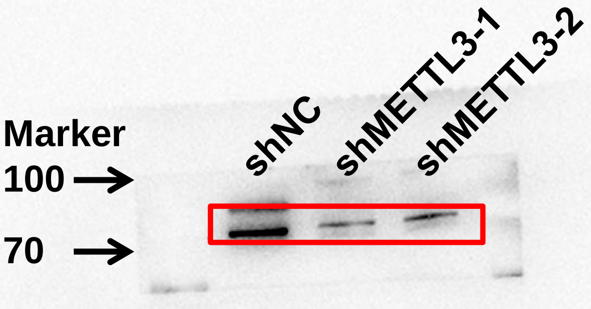

After the membrane was transferred, the membrane was cut from 70-100kd, and the part of the membrane on 70-100kd was incubated with **METTL3** antibody, and band detection was performed.

Figure S2c

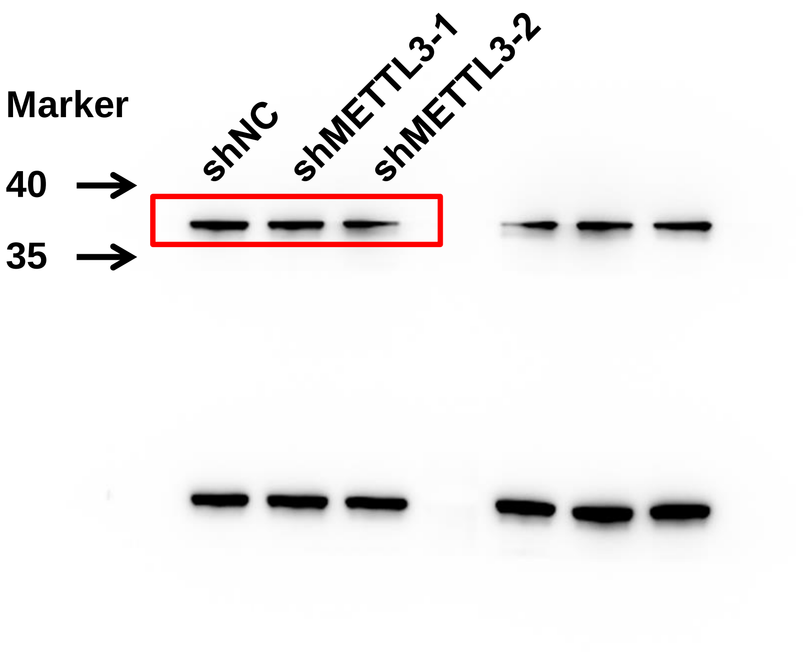

After the membrane was transferred, the membrane was cut from 35-40kd, and the part of the membrane on 35-40 kd was incubated with **GAPDH** antibody, and band detection was performed.

Figure S2f

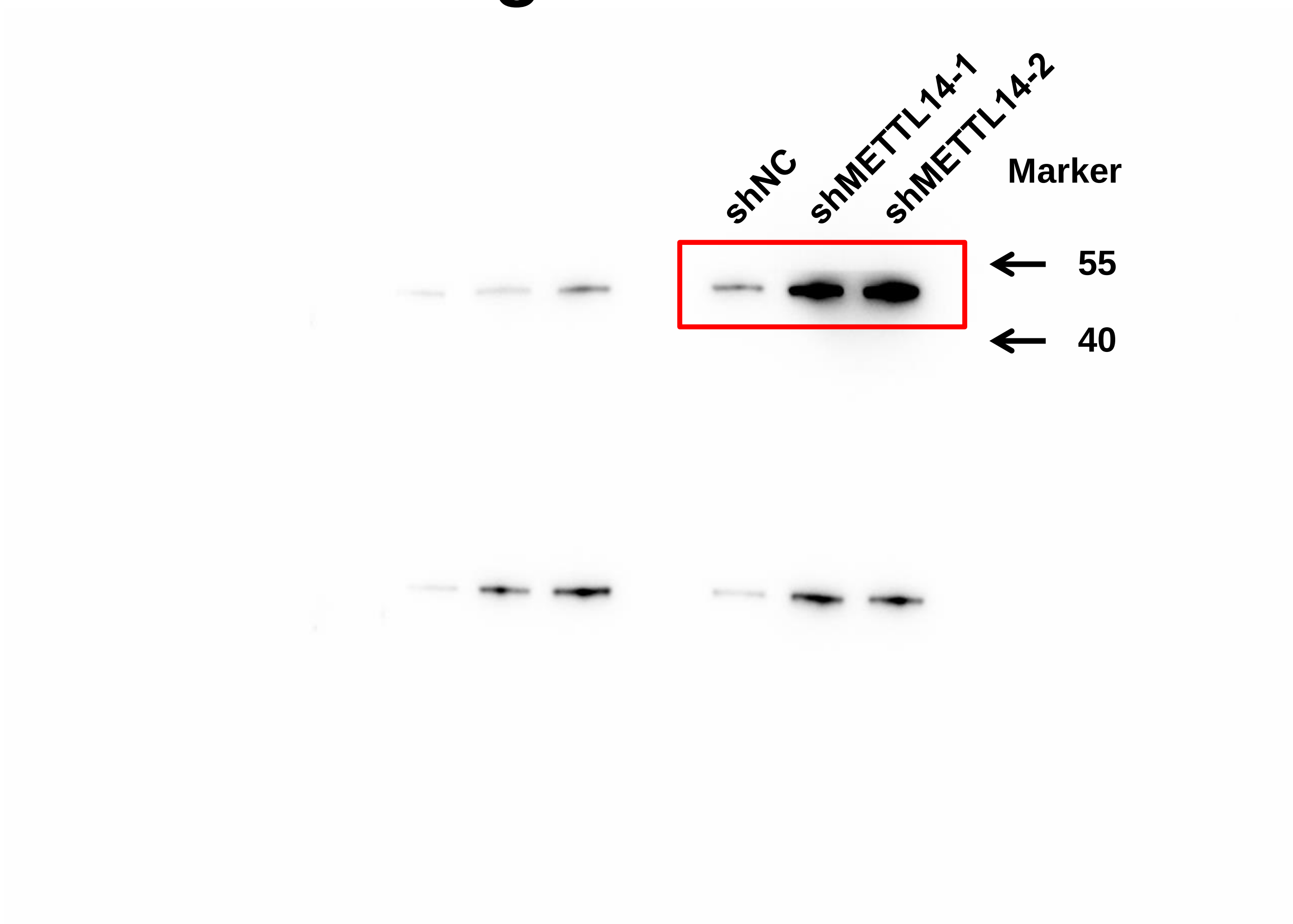

After the membrane was transferred, the membrane was cut from 40-55kd, and the part of the membrane on 40-55 kd was incubated with **SARS-CoV-2 Nucleoprotein Rabbit pAb** antibody, and band detection was performed.

Figure S2f

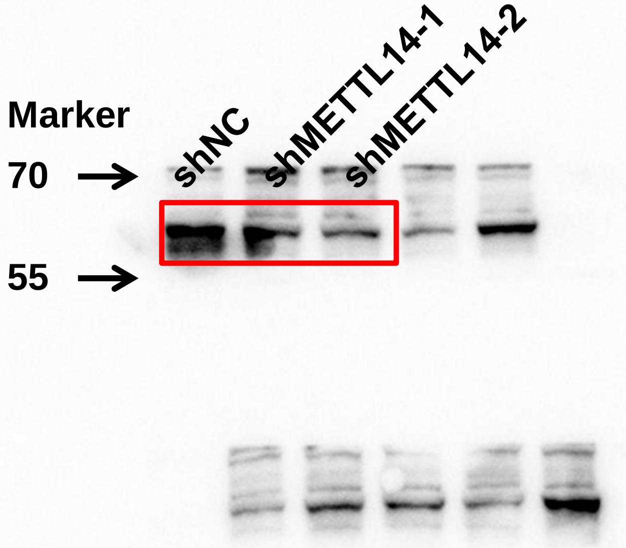

After the membrane was transferred, the membrane was cut from 55-70kd, and the part of the membrane on 55-70kd was incubated with **METTL14** antibody, and band detection was performed.

Figure S2f

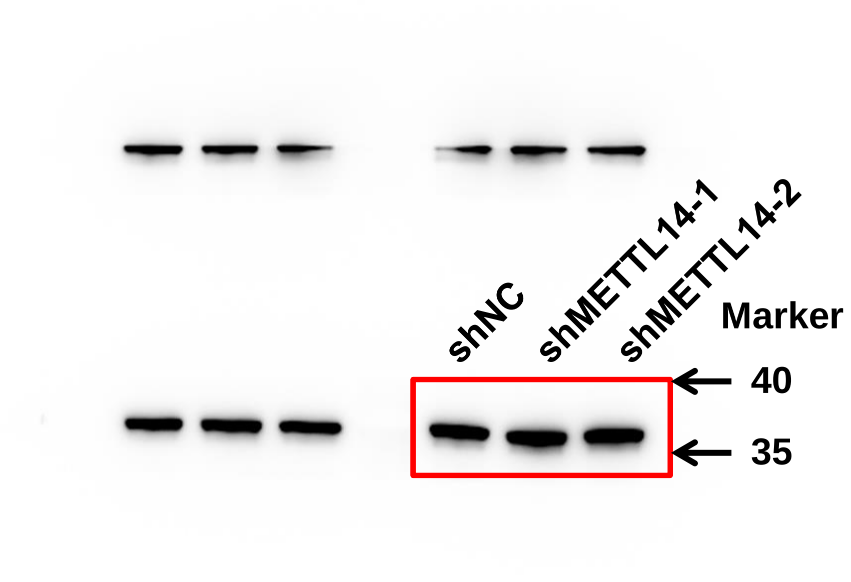

After the membrane was transferred, the membrane was cut from 35-40kd, and the part of the membrane on 35-40 kd was incubated with **GAPDH** antibody, and band detection was performed.

Figure S2i

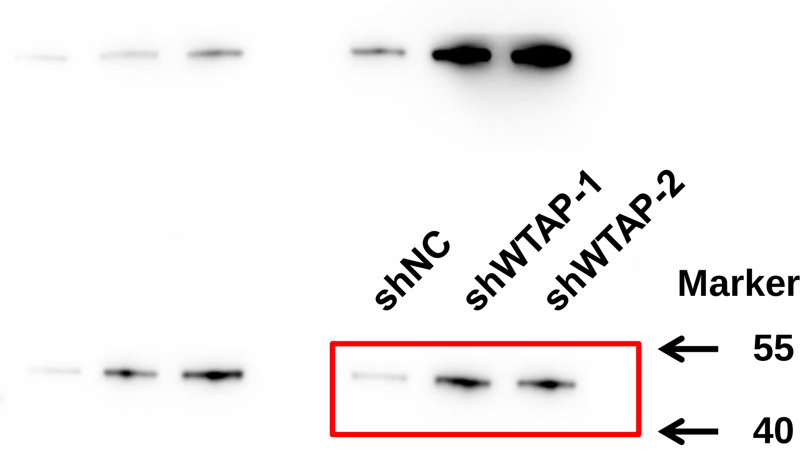

After the membrane was transferred, the membrane was cut from 40-55kd, and the part of the membrane on 40-55 kd was incubated with **SARS-CoV-2 Nucleoprotein Rabbit pAb** antibody, and band detection was performed.

Figure S2i

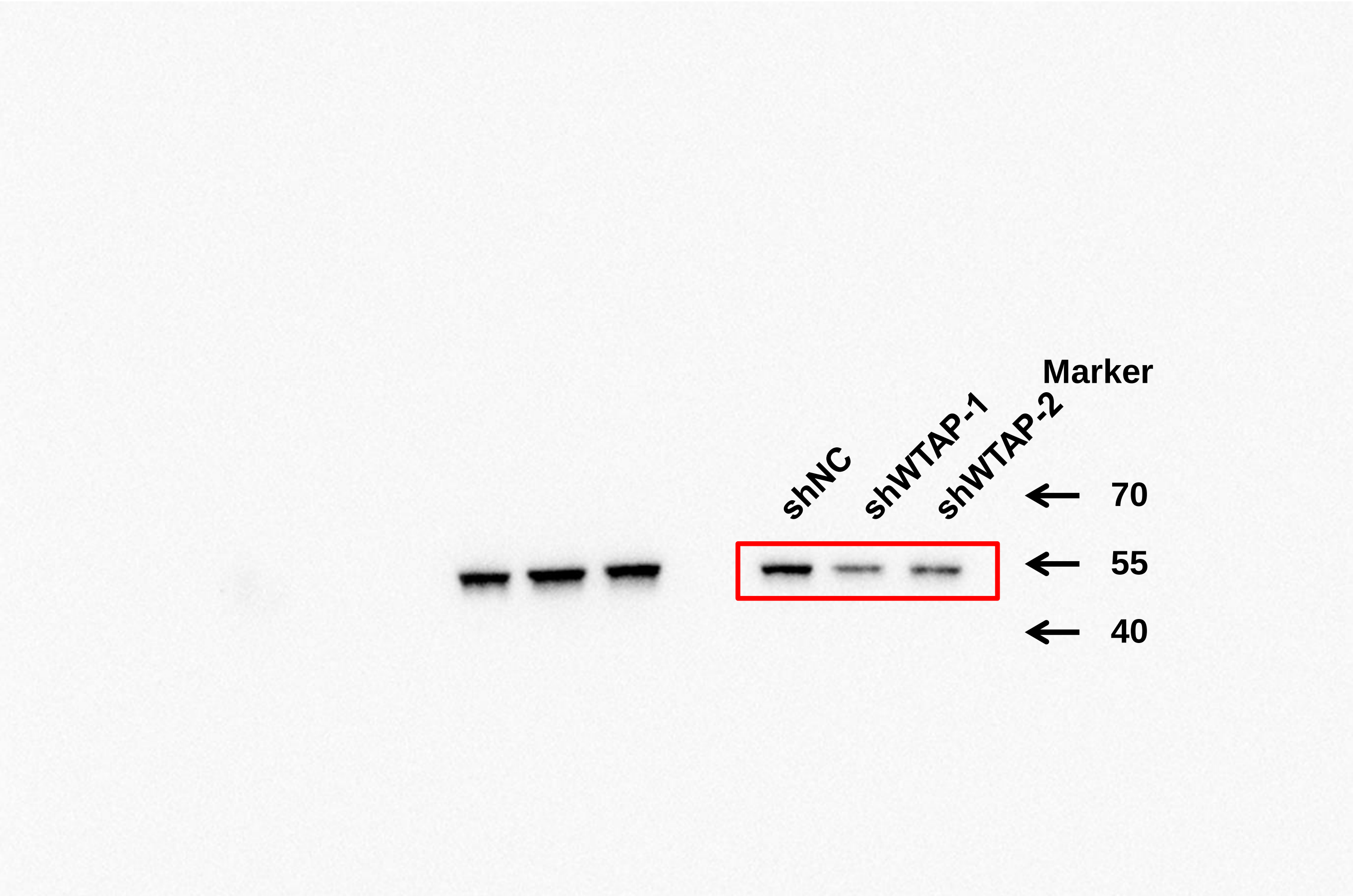

After the membrane was transferred, the membrane was cut from 40-70kd, and the part of the membrane on 40-70kd was incubated with **WTAP** antibody, and band detection was performed.

Figure S2i

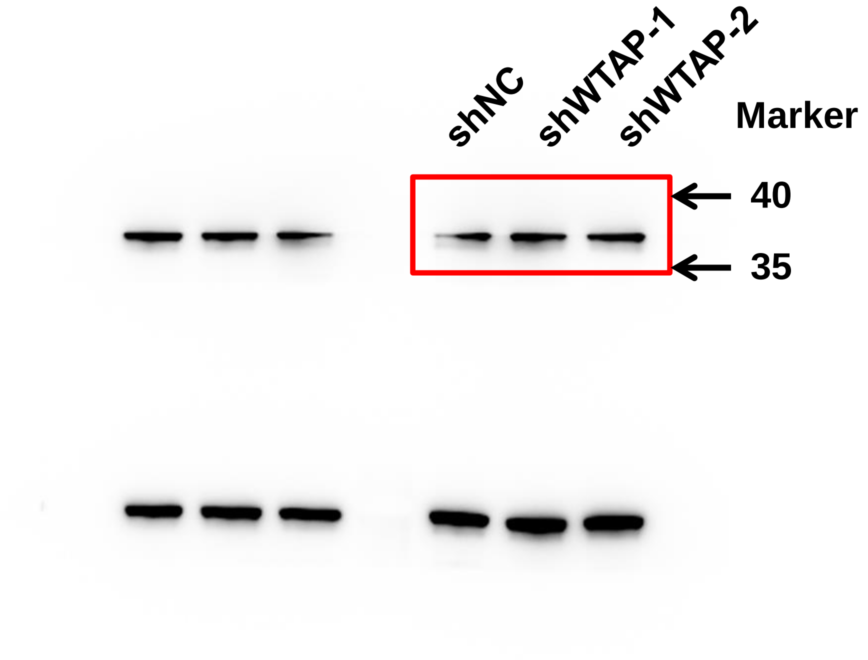

After the membrane was transferred, the membrane was cut from 35-40kd, and the part of the membrane on 35-40 kd was incubated with **GAPDH** antibody, and band detection was performed.

Figure S2I

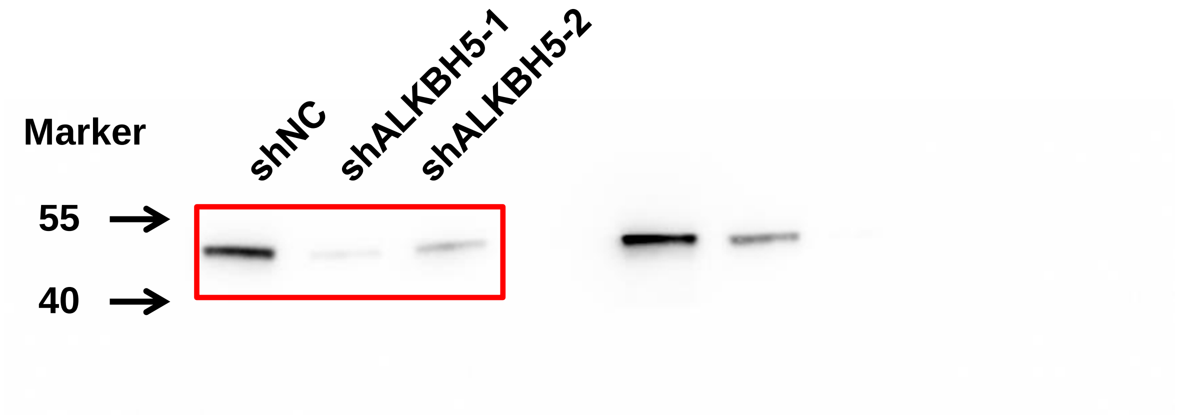

After the membrane was transferred, the membrane was cut from 40-55kd, and the part of the membrane on 40-55 kd was incubated with **SARS-CoV-2 Nucleoprotein Rabbit pAb** antibody, and band detection was performed.

Figure S2I

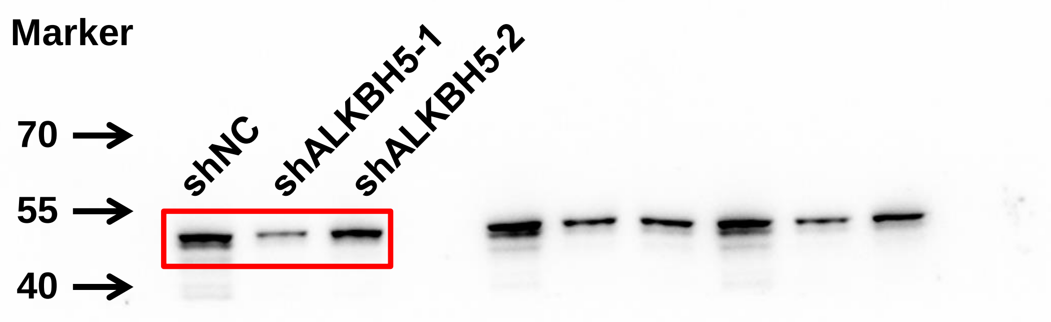

After the membrane was transferred, the membrane was cut from 40-70kd, and the part of the membrane on 40-70kd was incubated with **ALKBH5** antibody, and band detection was performed.

Figure S2I

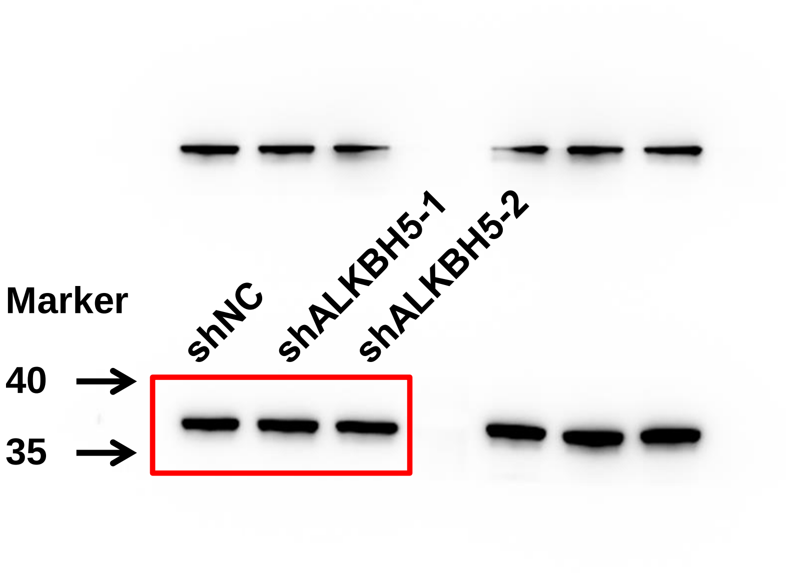

After the membrane was transferred, the membrane was cut from 35-40kd, and the part of the membrane on 35-40 kd was incubated with **GAPDH** antibody, and band detection was performed.

Figure S3a

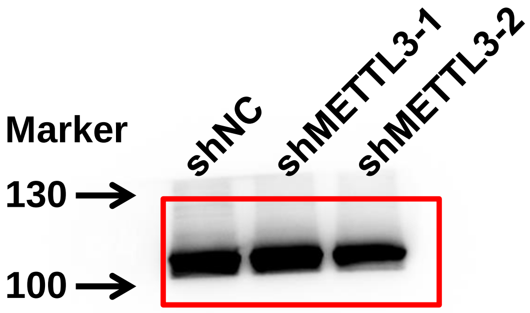

After the membrane was transferred, the membrane was cut from 100-130kd, and the part of the membrane on 100-130 kd was incubated with **ACTN4** antibody, and band detection was performed.

Figure S3a

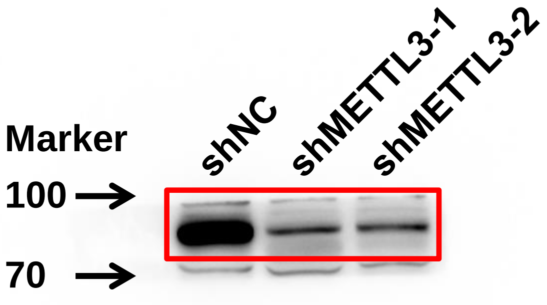

After the membrane was transferred, the membrane was cut from 70-100kd, and the part of the membrane on 70-100kd was incubated with **METTL3** antibody, and band detection was performed.

Figure S3a

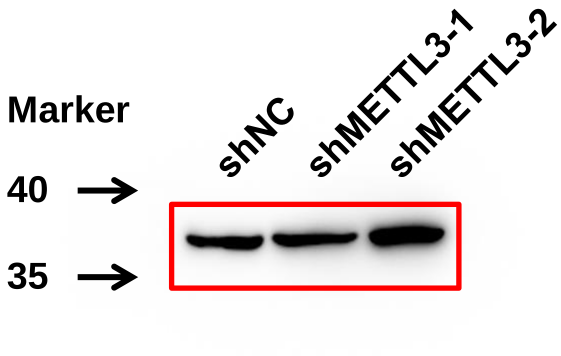

After the membrane was transferred, the membrane was cut from 35-40kd, and the part of the membrane on 35-40 kd was incubated with **GAPDH** antibody, and band detection was performed.

Figure S3c

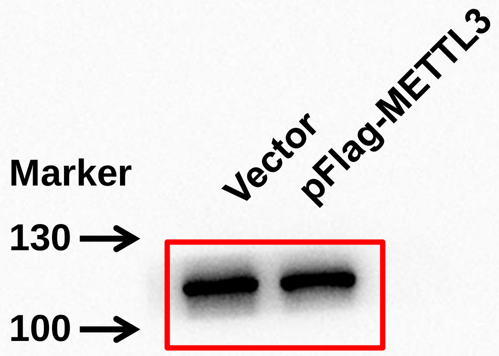

After the membrane was transferred, the membrane was cut from 100-130kd, and the part of the membrane on 100-130 kd was incubated with **ACTN4** antibody, and band detection was performed.

Figure S3c

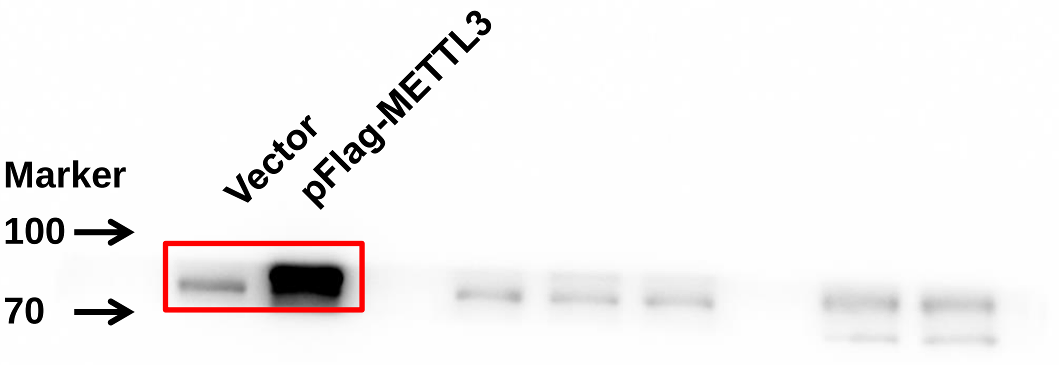

After the membrane was transferred, the membrane was cut from 70-100kd, and the part of the membrane on 70-100kd was incubated with **METTL3** antibody, and band detection was performed.

Figure S3c

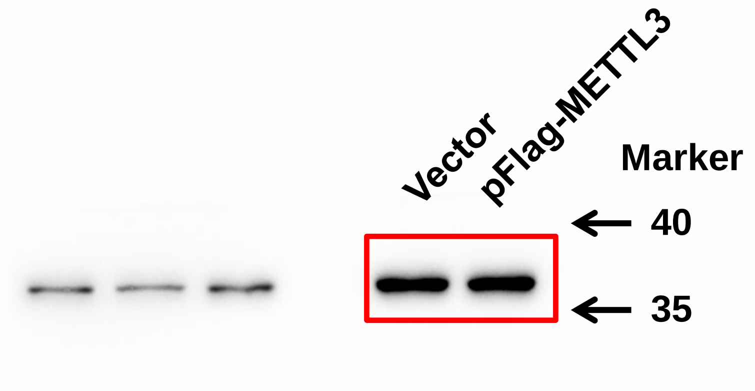

After the membrane was transferred, the membrane was cut from 35-40kd, and the part of the membrane on 35-40 kd was incubated with **GAPDH** antibody, and band detection was performed.

Figure S3e

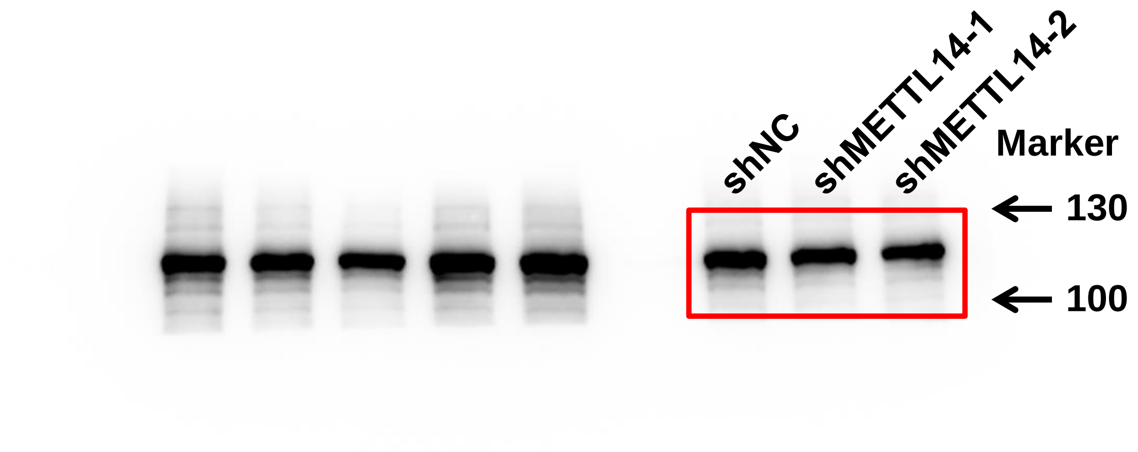

After the membrane was transferred, the membrane was cut from 100-130kd, and the part of the membrane on 100-130 kd was incubated with **ACTN4** antibody, and band detection was performed.

Figure S3e

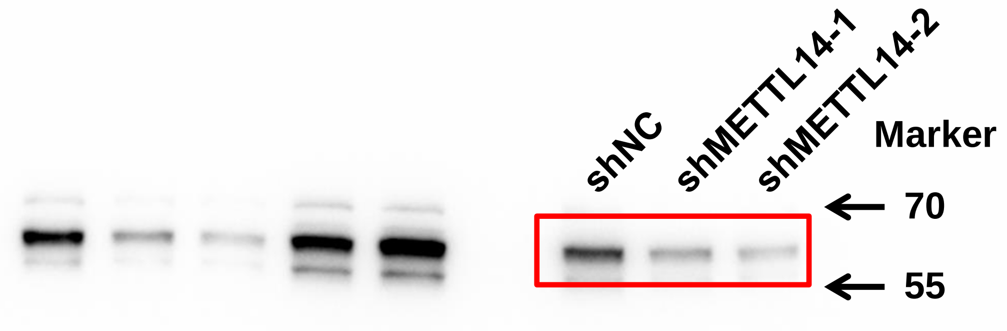

After the membrane was transferred, the membrane was cut from 55-70kd, and the part of the membrane on 55-70kd was incubated with **METTL14** antibody, and band detection was performed.

Figure S3e

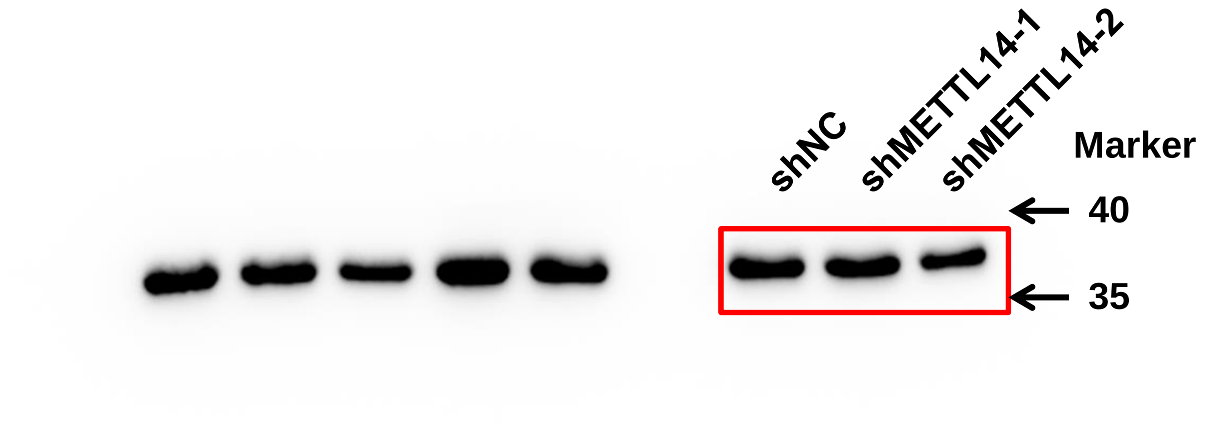

After the membrane was transferred, the membrane was cut from 35-40kd, and the part of the membrane on 35-40 kd was incubated with **GAPDH** antibody, and band detection was performed.

Figure S3g

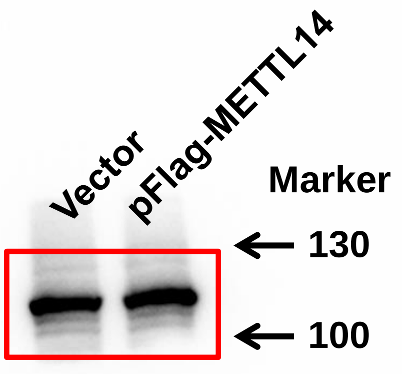

After the membrane was transferred, the membrane was cut from 100-130kd, and the part of the membrane on 100-130 kd was incubated with **ACTN4** antibody, and band detection was performed.

Figure S3g

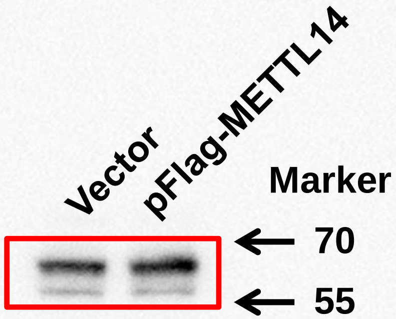

After the membrane was transferred, the membrane was cut from 55-70kd, and the part of the membrane on 55-70kd was incubated with **METTL14** antibody, and band detection was performed.

Figure S3g

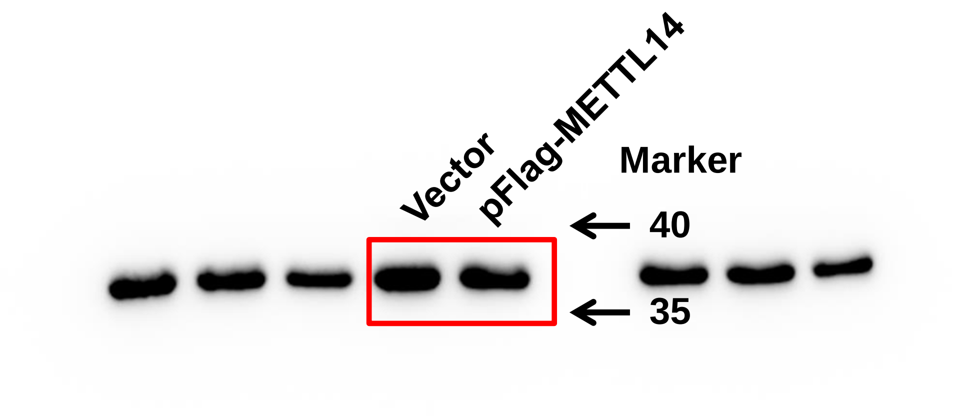

After the membrane was transferred, the membrane was cut from 35-40kd, and the part of the membrane on 35-40 kd was incubated with **GAPDH** antibody, and band detection was performed.

Figure S3i

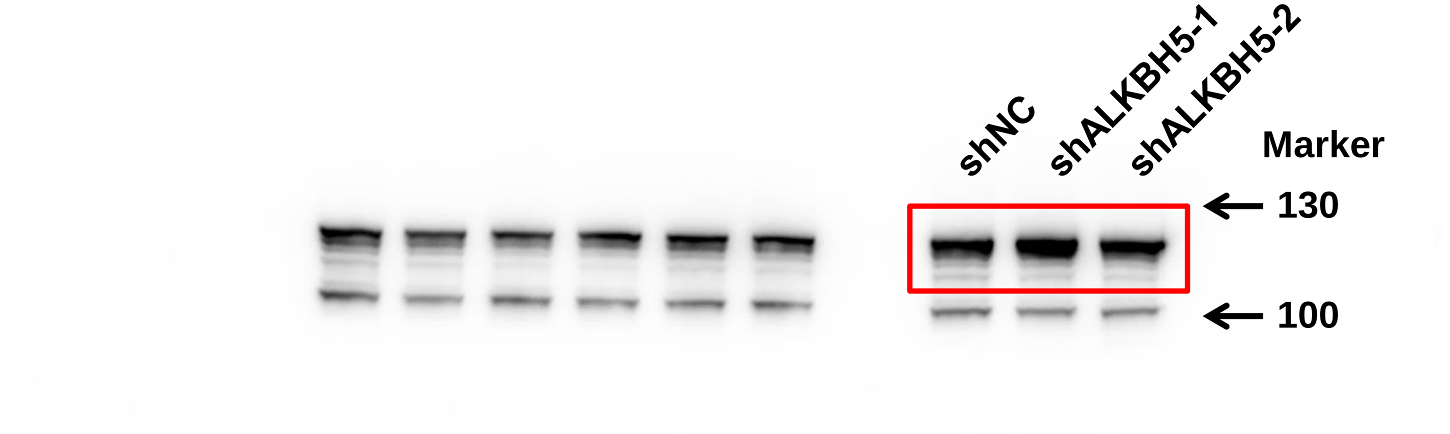

After the membrane was transferred, the membrane was cut from 100-130kd, and the part of the membrane on 100-130 kd was incubated with **ACTN4** antibody, and band detection was performed.

Figure S3i

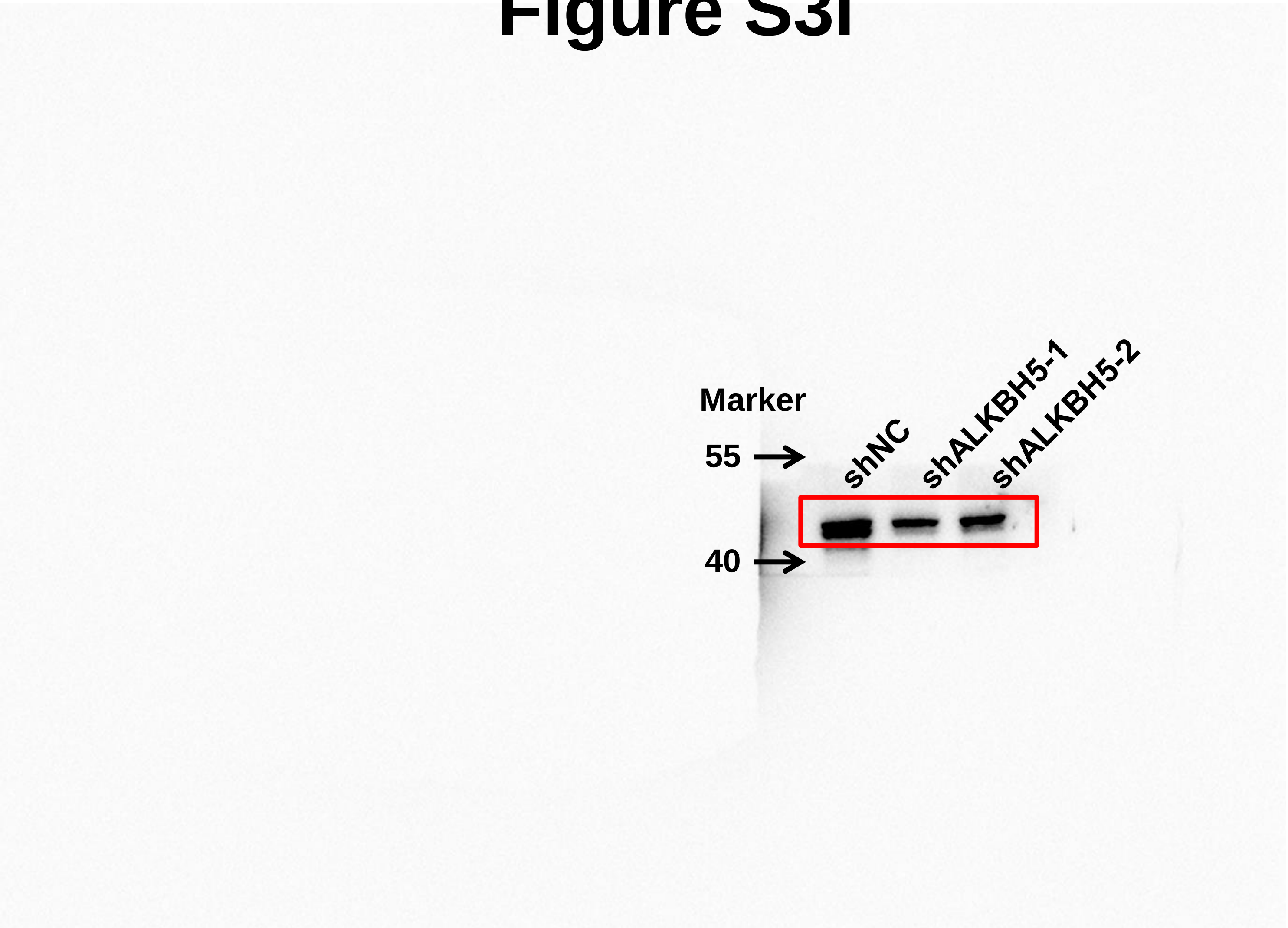

After the membrane was transferred, the membrane was cut from 40-70kd, and the part of the membrane on 40-70kd was incubated with **ALKBH5** antibody, and band detection was performed.

Figure S3i

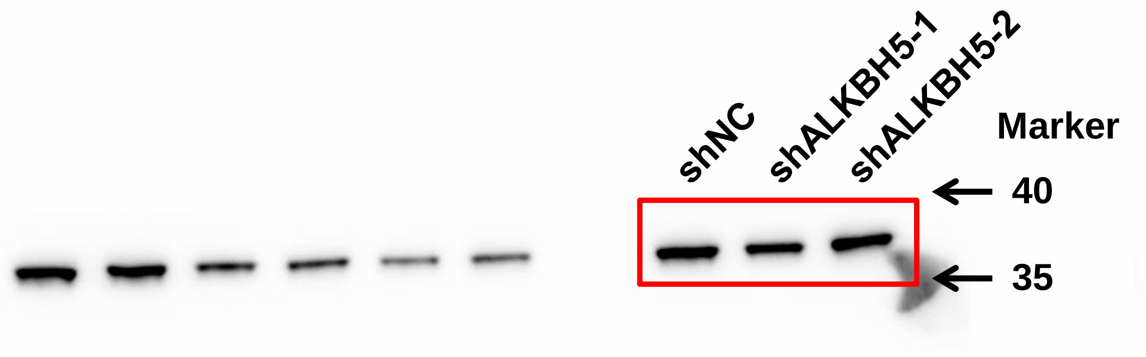

After the membrane was transferred, the membrane was cut from 35-40kd, and the part of the membrane on 35-40 kd was incubated with **GAPDH** antibody, and band detection was performed.

Figure S3k

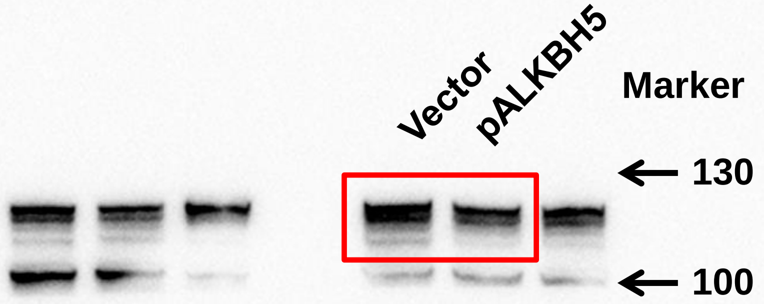

After the membrane was transferred, the membrane was cut from 100-130kd, and the part of the membrane on 100-130 kd was incubated with **ACTN4** antibody, and band detection was performed.

Figure S3k

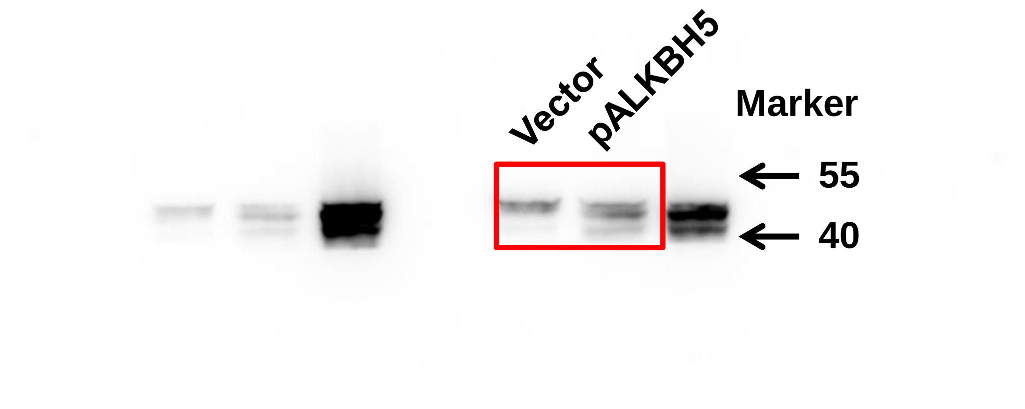

After the membrane was transferred, the membrane was cut from 40-70kd, and the part of the membrane on 40-70kd was incubated with **ALKBH5** antibody, and band detection was performed.

Figure S3k

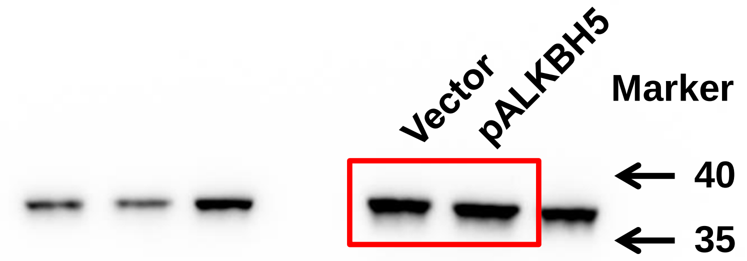

After the membrane was transferred, the membrane was cut from 35-40kd, and the part of the membrane on 35-40 kd was incubated with **GAPDH** antibody, and band detection was performed.

Figure S3m

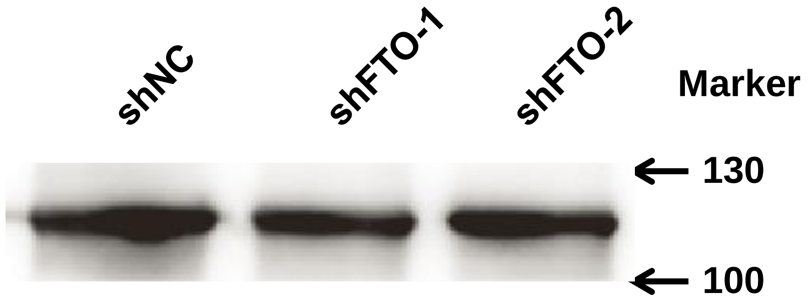

After the membrane was transferred, the membrane was cut from 100-130kd, and the part of the membrane on 100-130 kd was incubated with **ACTN4** antibody, and band detection was performed.

Figure S3m

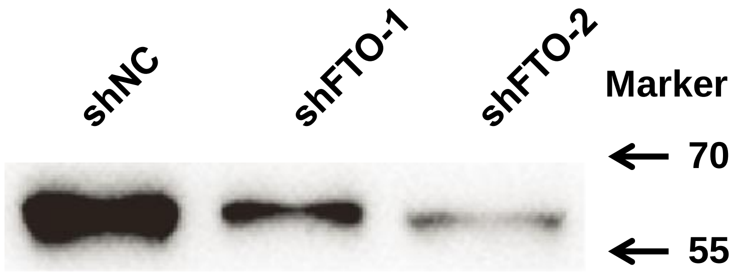

After the membrane was transferred, the membrane was cut from 55-70kd, and the part of the membrane on 55-70kd was incubated with **FTO** antibody, and band detection was performed.

Figure S3m

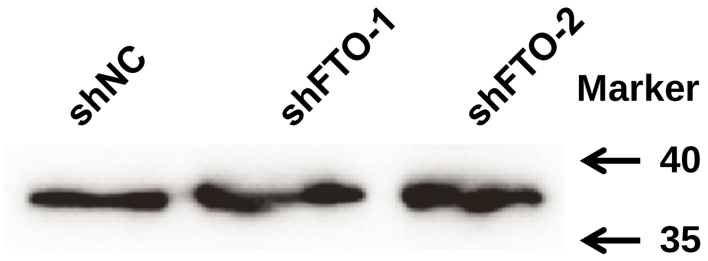

After the membrane was transferred, the membrane was cut from 35-40kd, and the part of the membrane on 35-40 kd was incubated with **GAPDH** antibody, and band detection was performed.

Figure S3o

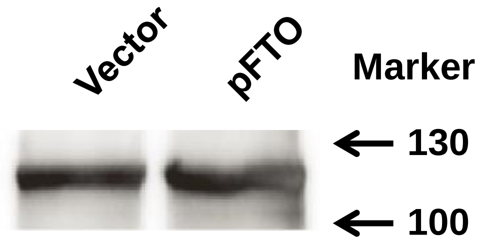

After the membrane was transferred, the membrane was cut from 100-130kd, and the part of the membrane on 100-130 kd was incubated with **ACTN4** antibody, and band detection was performed.

Figure S3o

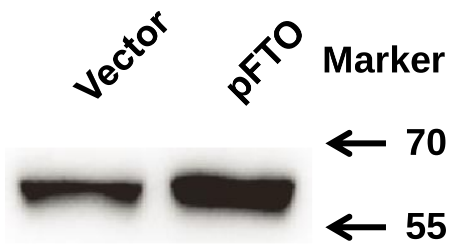

After the membrane was transferred, the membrane was cut from 40-70kd, and the part of the membrane on 40-70kd was incubated with **FTO** antibody, and band detection was performed.

Figure S3o

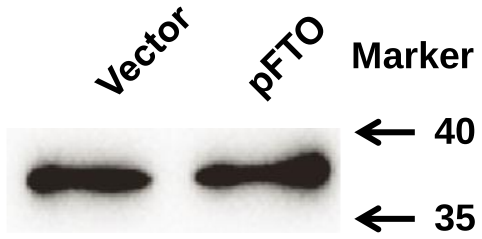

After the membrane was transferred, the membrane was cut from 35-40kd, and the part of the membrane on 35-40 kd was incubated with **GAPDH** antibody, and band detection was performed.

Figure S4a

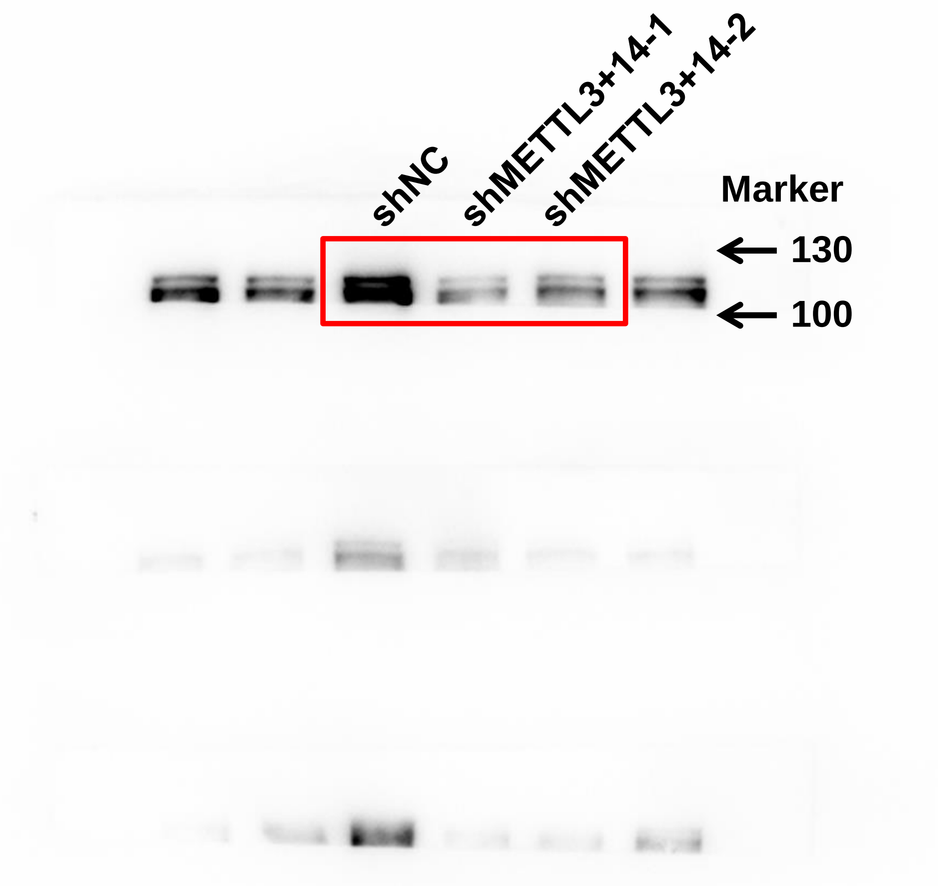

After the membrane was transferred, the membrane was cut from 100-130kd, and the part of the membrane on 100-130 kd was incubated with **ACTN4** antibody, and band detection was performed.

Figure S4a

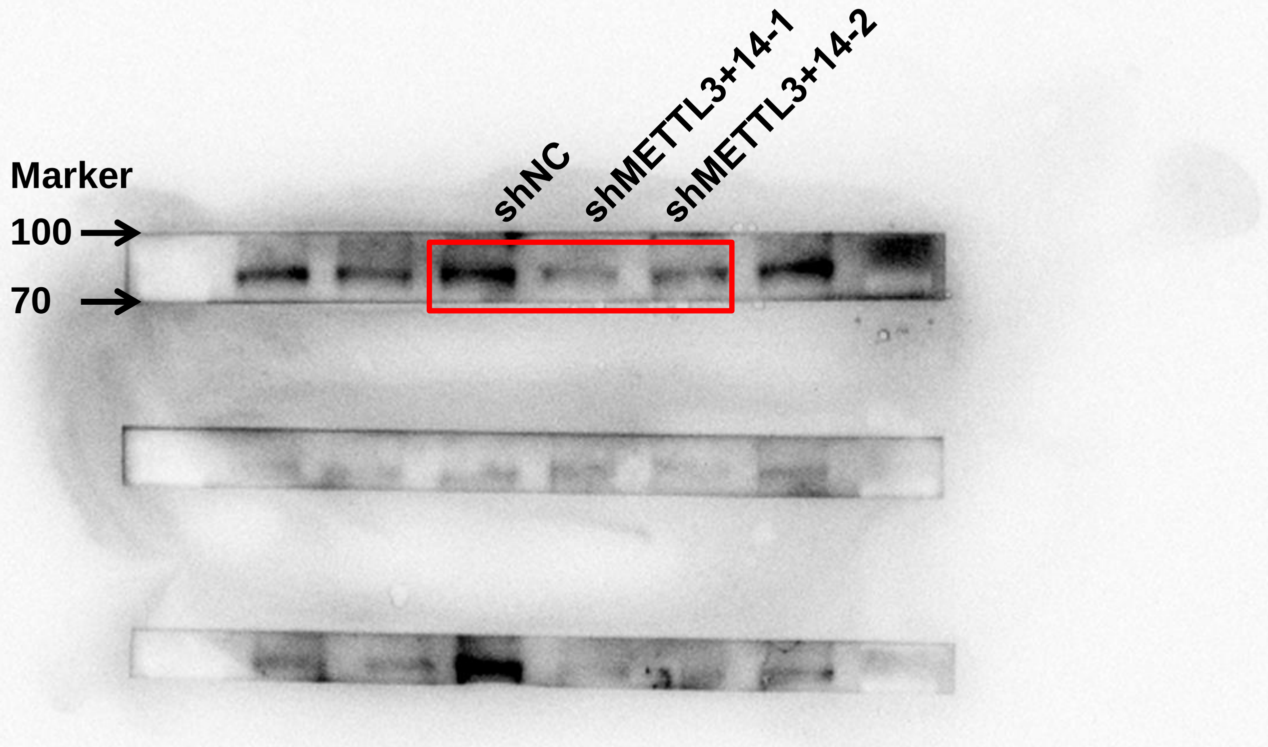

After the membrane was transferred, the membrane was cut from 70-100kd, and the part of the membrane on 70-100kd was incubated with **METTL3** antibody, and band detection was performed.

Figure S4a

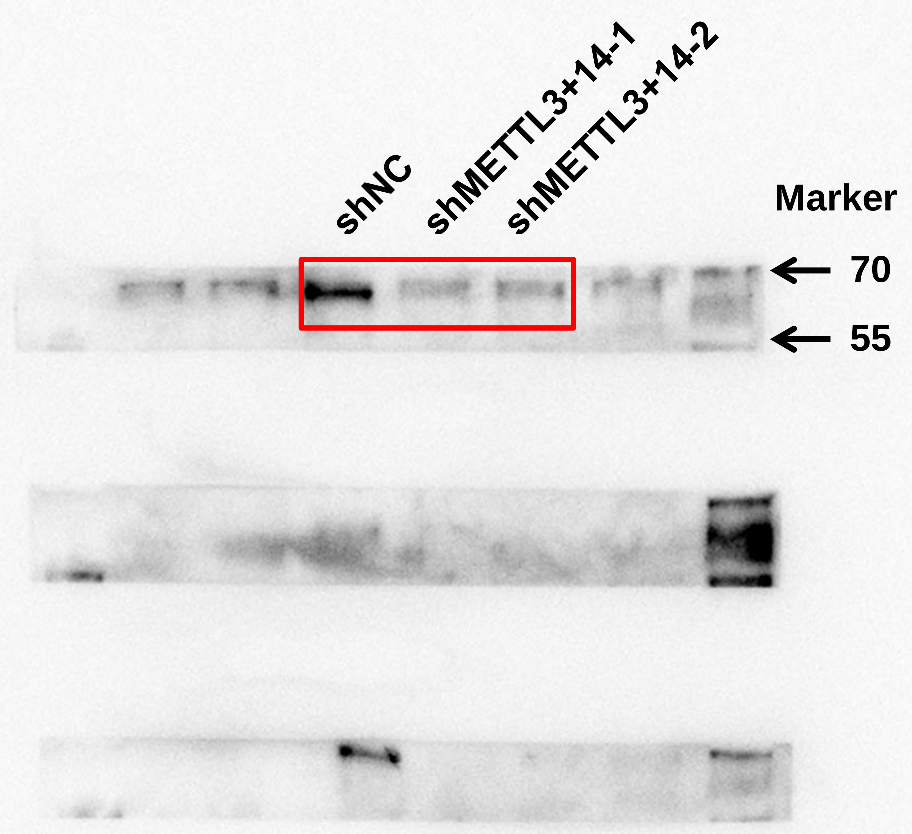

After the membrane was transferred, the membrane was cut from 55-70kd, and the part of the membrane on 55-70kd was incubated with **METTL14** antibody, and band detection was performed.

Figure S4a

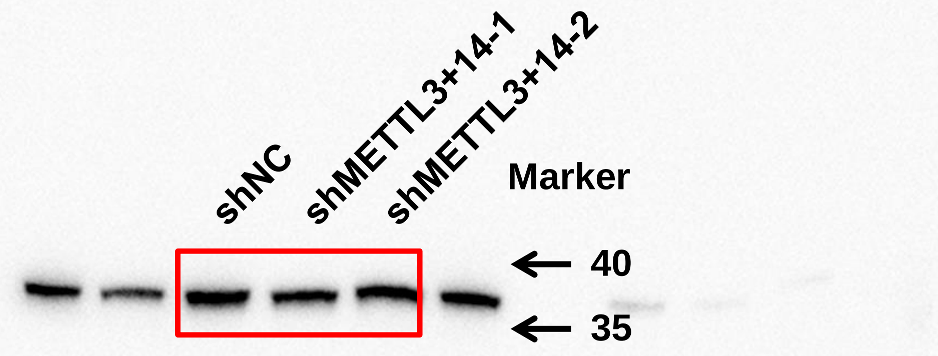

After the membrane was transferred, the membrane was cut from 35-40kd, and the part of the membrane on 35-40 kd was incubated with **GAPDH** antibody, and band detection was performed.

Figure S5c and d

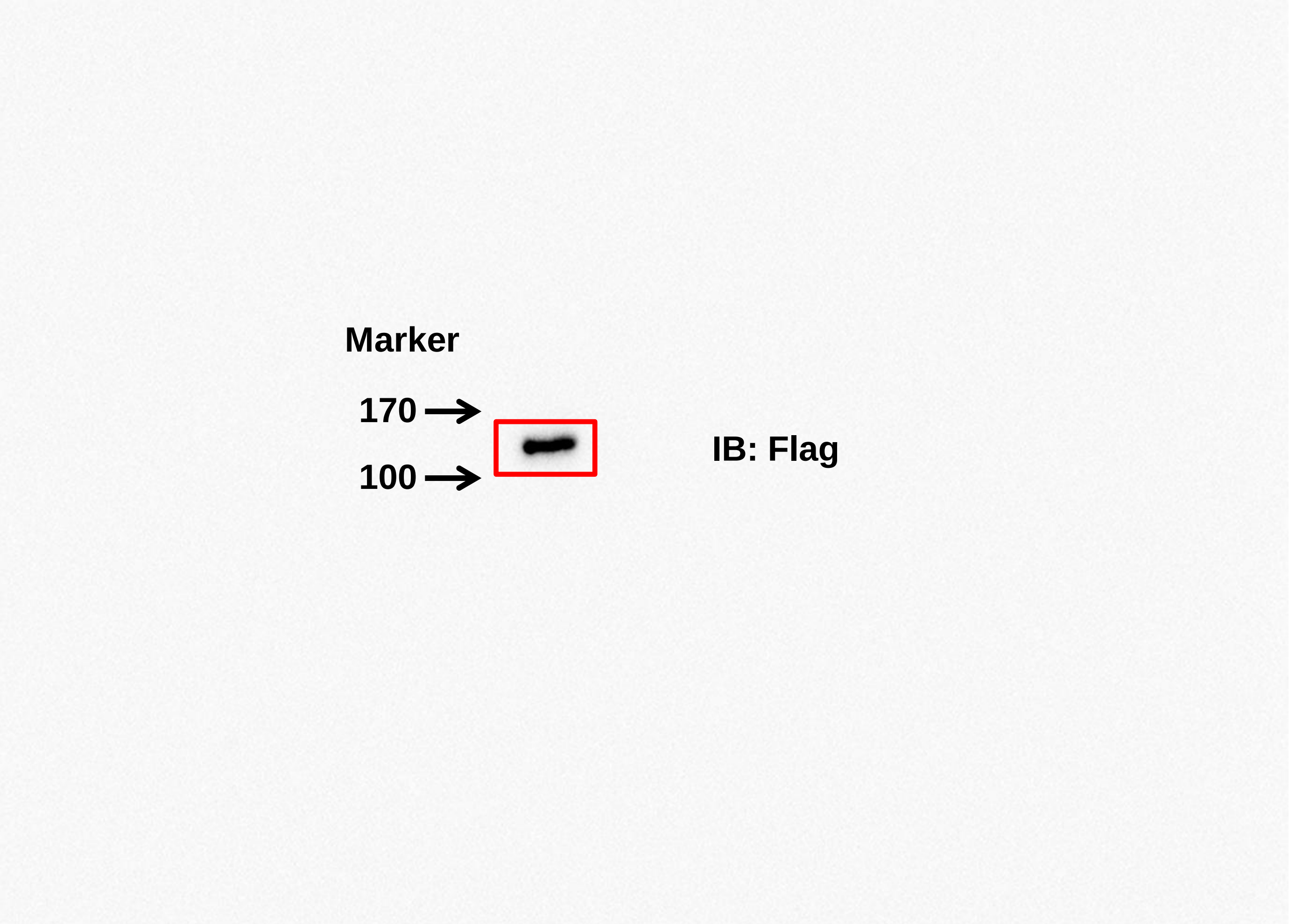

After the membrane was transferred, the membrane was cut from 100-170 kd, and the part of the membrane on 100-170 kd was incubated with **Flag** antibody, and band detection was performed.

Figure S5c and d

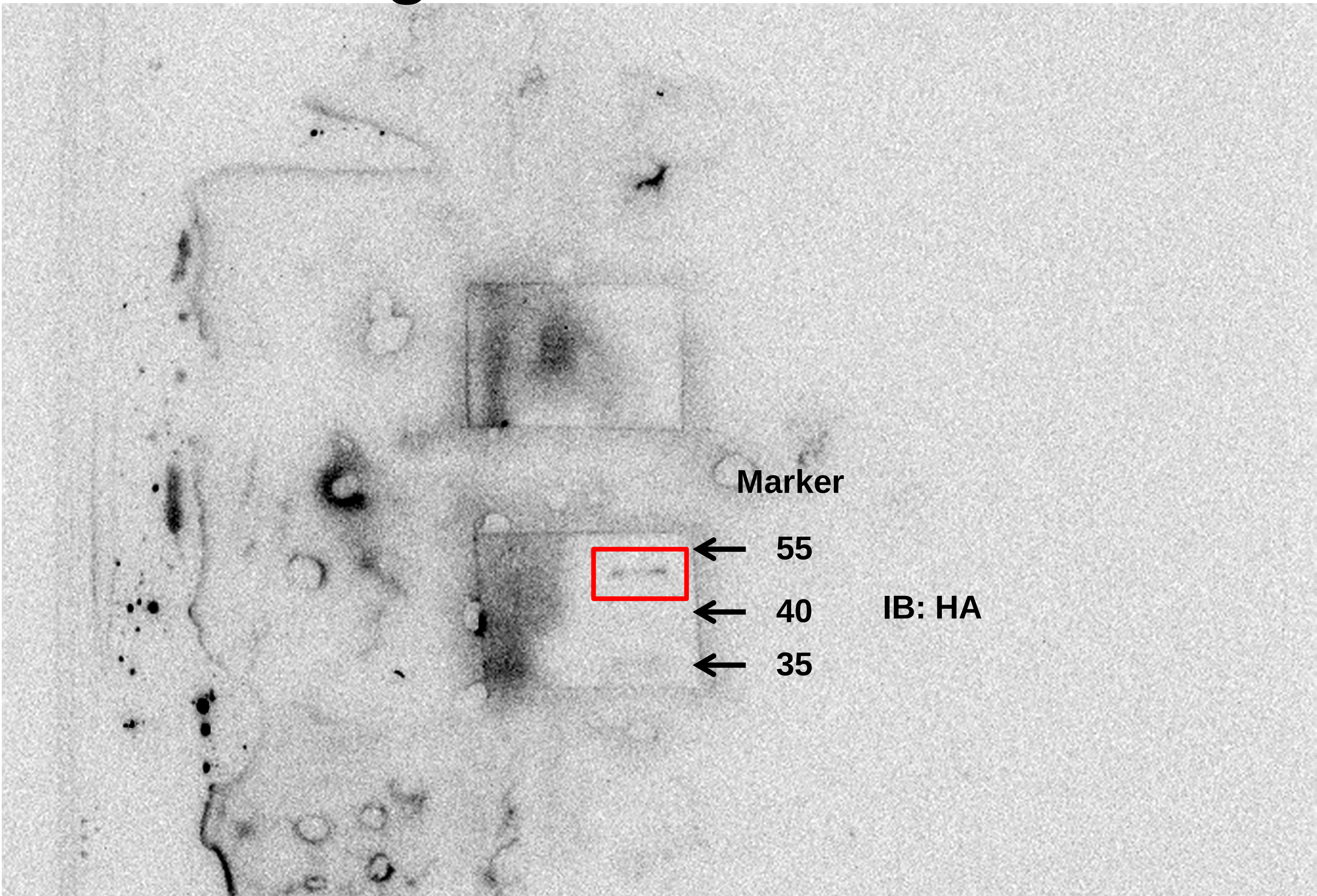

After the membrane was transferred, the membrane was cut from 35-55 kd, and the part of the membrane on 35-55 kd was incubated with **HA** antibody, and band detection was performed.

Figure S5c and d

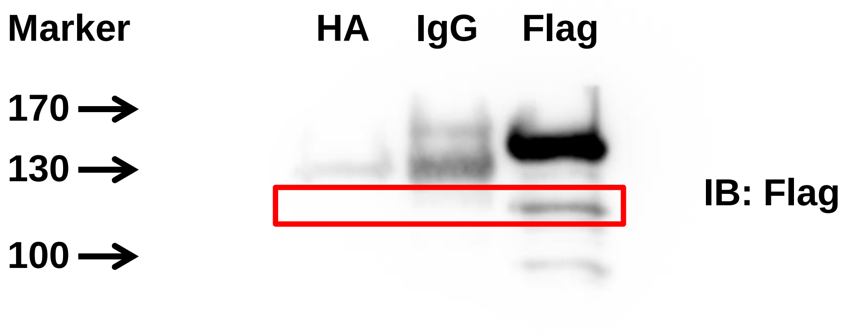

After the membrane was transferred, the membrane was cut from 100-170 kd, and the part of the membrane on 100-170 kd was incubated with **Flag** antibody, and band detection was performed.

Figure S5c and d

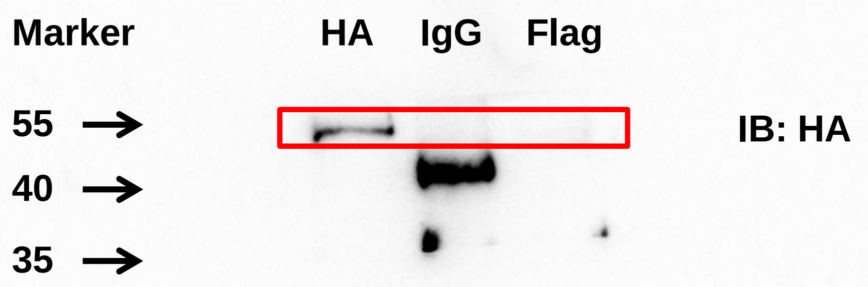

After the membrane was transferred, the membrane was cut from 35-55 kd, and the part of the membrane on 35-55 kd was incubated with **HA** antibody, and band detection was performed.

Figure S5e

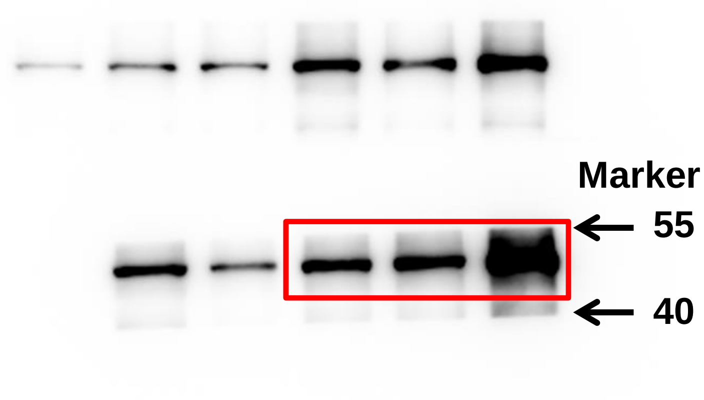

After the membrane was transferred, the membrane was cut from 40-55 kd, and the part of the membrane on 40-55 kd was incubated with **WTAP** antibody, and band detection was performed.

Figure S5e

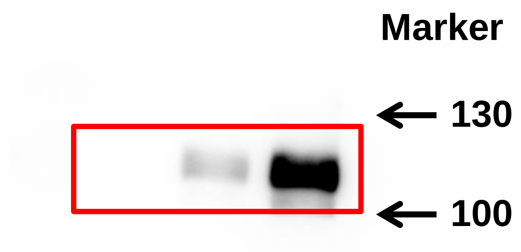

After the membrane was transferred, the membrane was cut from 100-130kd, and the part of the membrane on 100-130 kd was incubated with **ACTN4** antibody, and band detection was performed.

Figure S5e

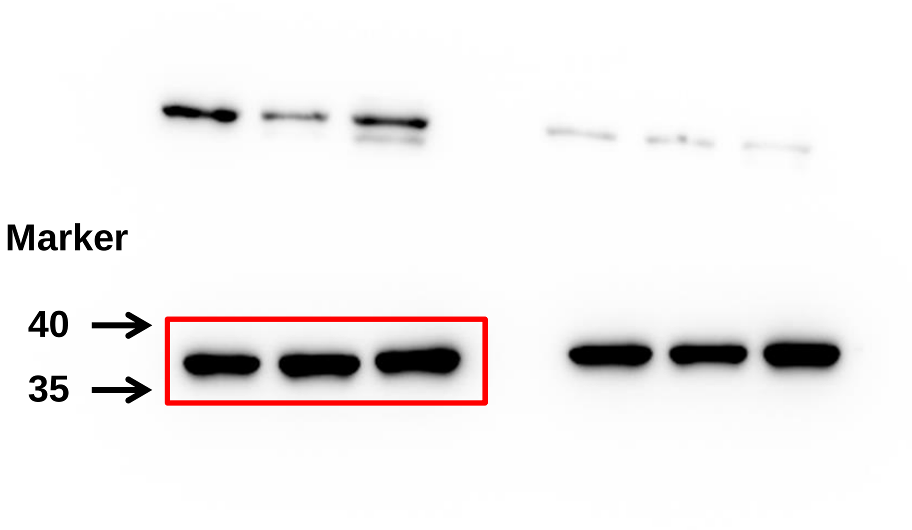

After the membrane was transferred, the membrane was cut from 35-40kd, and the part of the membrane on 35-40 kd was incubated with **GAPDH** antibody, and band detection was performed.

Figure S6a

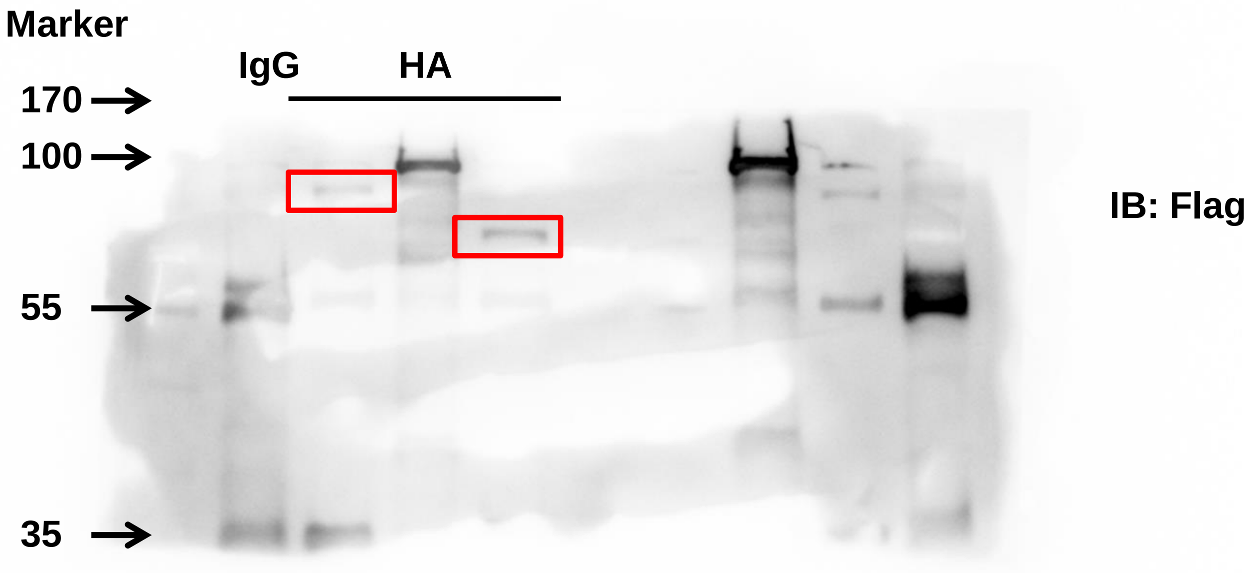

After the membrane was transferred, the membrane was cut from 35-170 kd, and the part of the membrane on 35-170 kd was incubated with **Flag** antibody, and band detection was performed.

Figure S6a

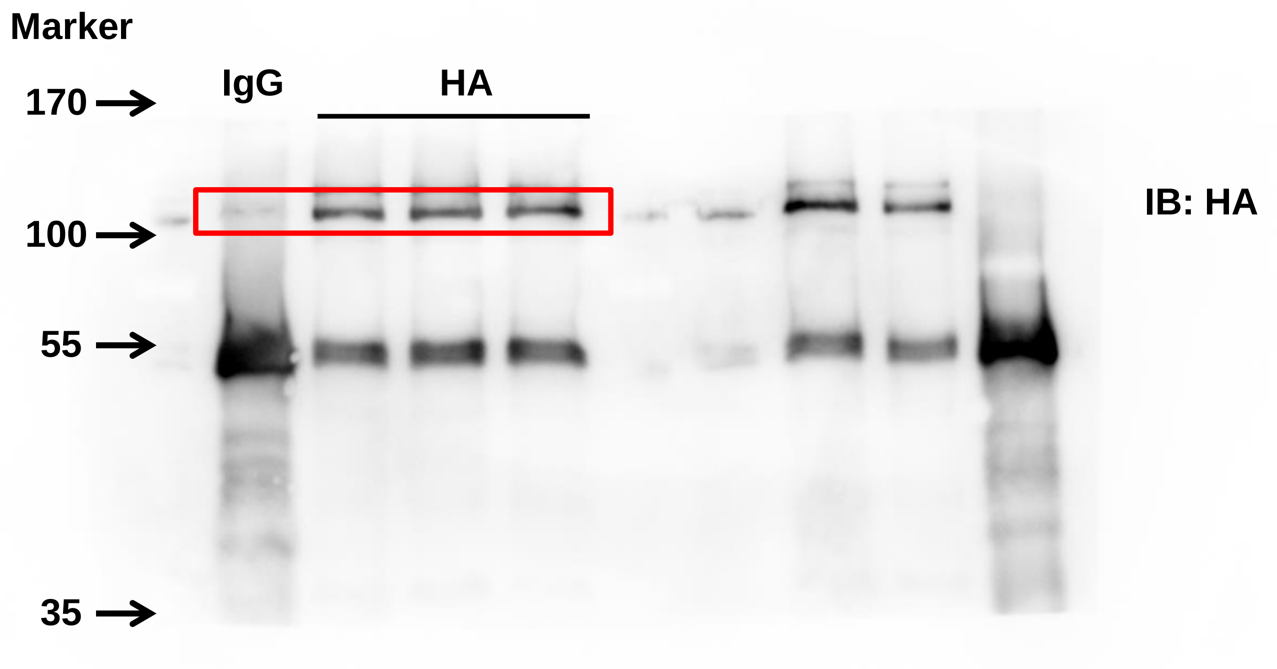

After the membrane was transferred, the membrane was cut from 35-130 kd, and the part of the membrane on 35-170 kd was incubated with **HA** antibody, and band detection was performed.

Figure S6b

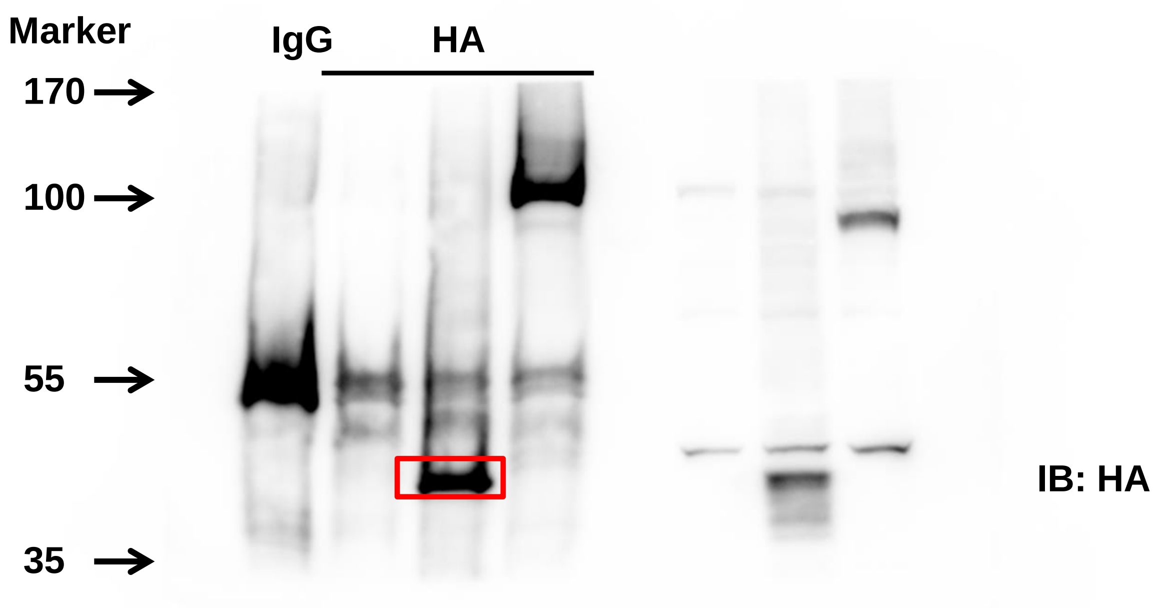

After the membrane was transferred, the membrane was cut from 35-170 kd, and the part of the membrane on 35-170 kd was incubated with **HA** antibody, and band detection was performed.

Figure S6b

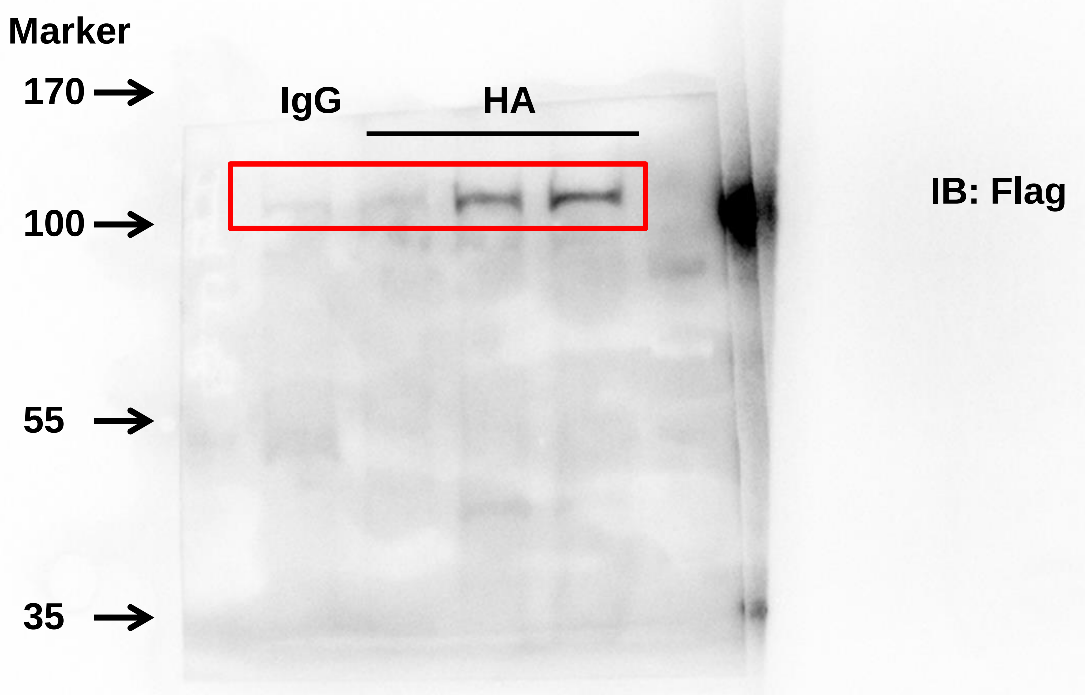

After the membrane was transferred, the membrane was cut from 35-170 kd, and the part of the membrane on 35-170 kd was incubated with **Flag** antibody, and band detection was performed.

Figure S6c and d

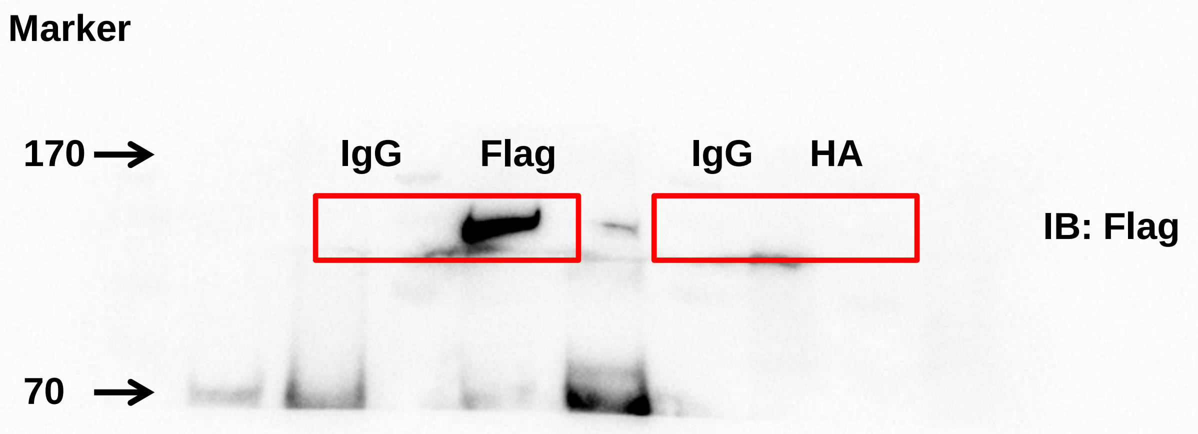

After the membrane was transferred, the membrane was cut from 70-170 kd, and the part of the membrane on 70-170 kd was incubated with **Flag** antibody, and band detection was performed.

Figure S6c and d

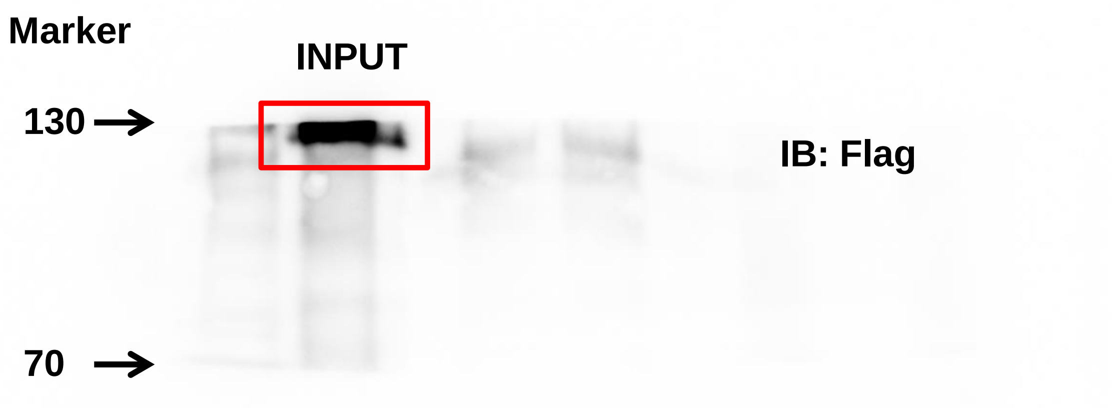

After the membrane was transferred, the membrane was cut from 70-130 kd, and the part of the membrane on 70-130 kd was incubated with **Flag** antibody, and band detection was performed.

Figure S6c and d

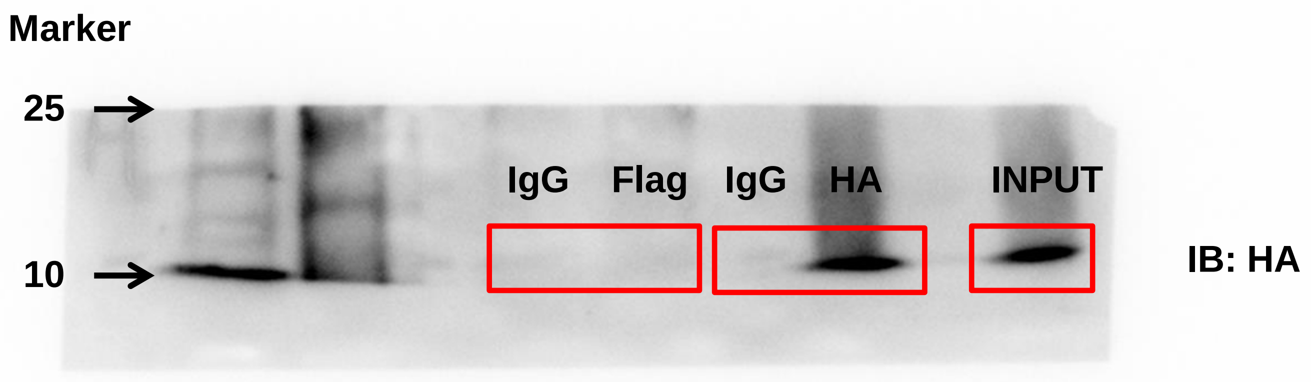

After the membrane was transferred, the membrane was cut from 10-25 kd, and the part of the membrane on 10-25 kd was incubated with **HA** antibody, and band detection was performed.

Figure S6e and f

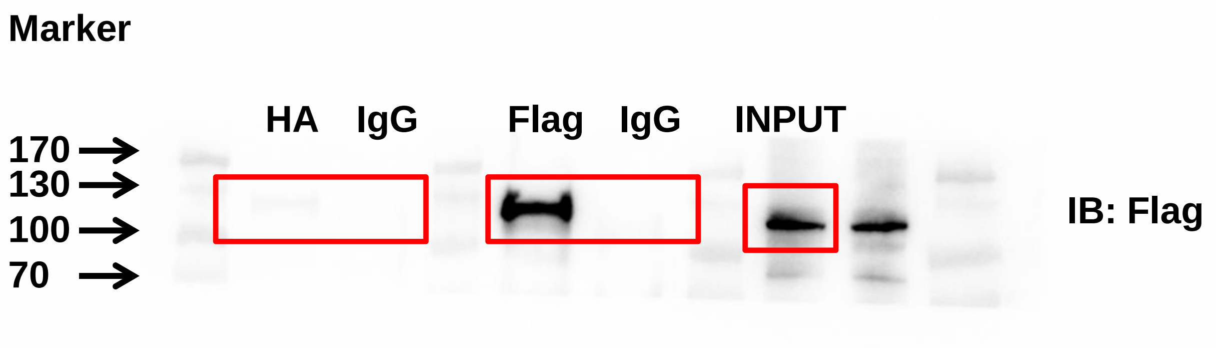

After the membrane was transferred, the membrane was cut from 70-170kd, and the part of the membrane on 70-170 kd was incubated with **Flag** antibody, and band detection was performed.

Figure S6e and f

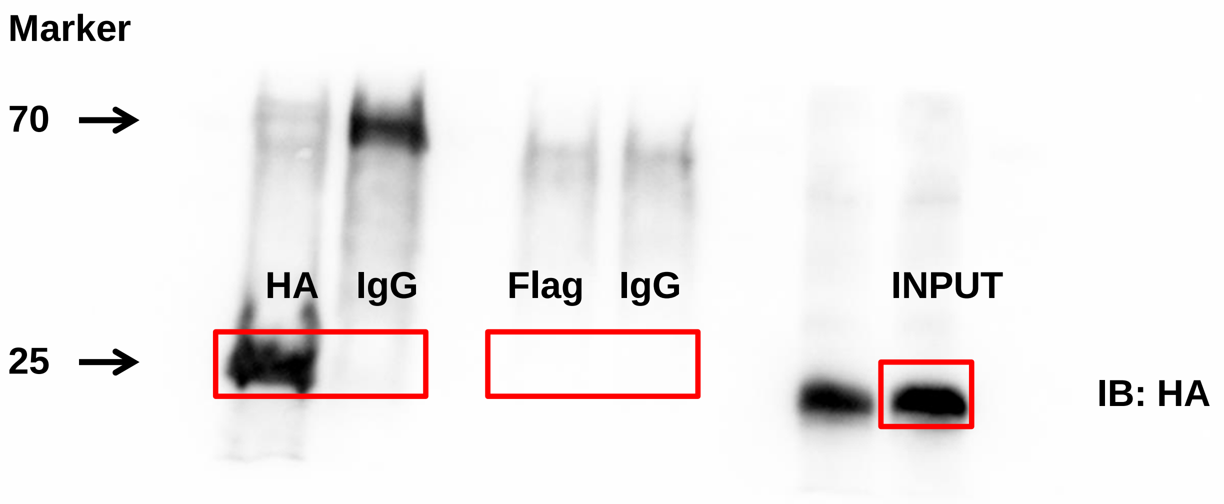

After the membrane was transferred, the membrane was cut from 25-70kd, and the part of the membrane on 25-70 kd was incubated with **HA** antibody, and band detection was performed.

Figure S7b

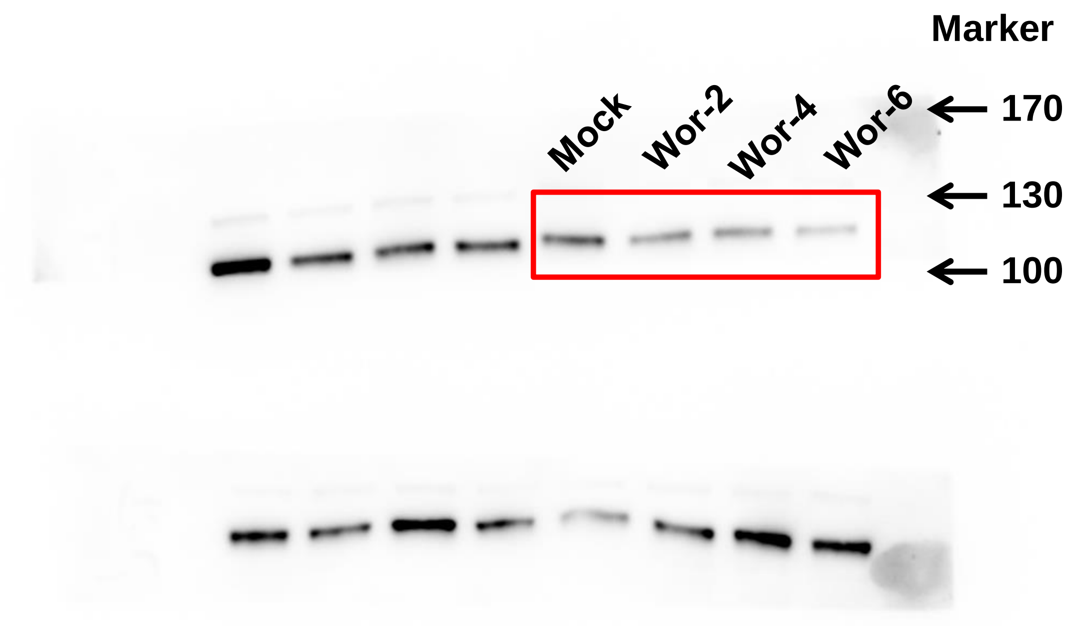

After the membrane was transferred, the membrane was cut from 100-170kd, and the part of the membrane on 100-170 kd was incubated with **ACTN4** antibody, and band detection was performed.

Figure S7b

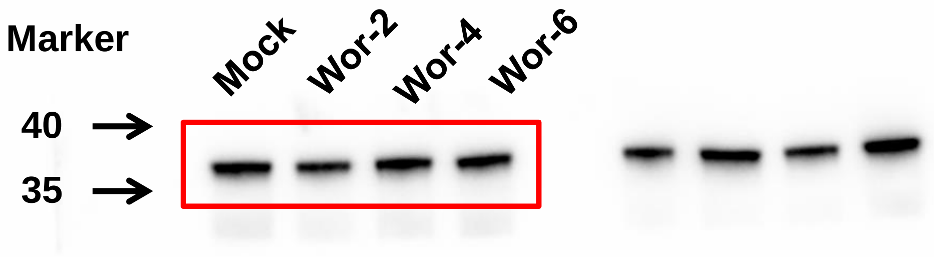

After the membrane was transferred, the membrane was cut from 35-40kd, and the part of the membrane on 35-40 kd was incubated with **GAPDH** antibody, and band detection was performed.

Figure S7d

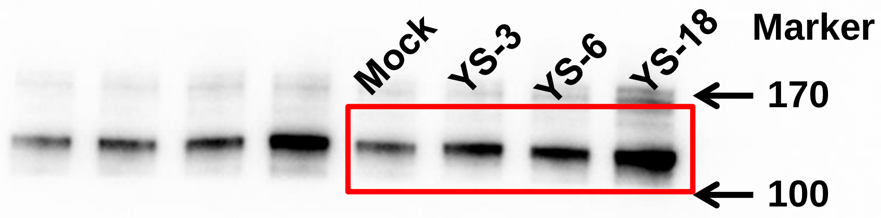

After the membrane was transferred, the membrane was cut from 100-170kd, and the part of the membrane on 100-170 kd was incubated with **ACTN4** antibody, and band detection was performed.

Figure S7d

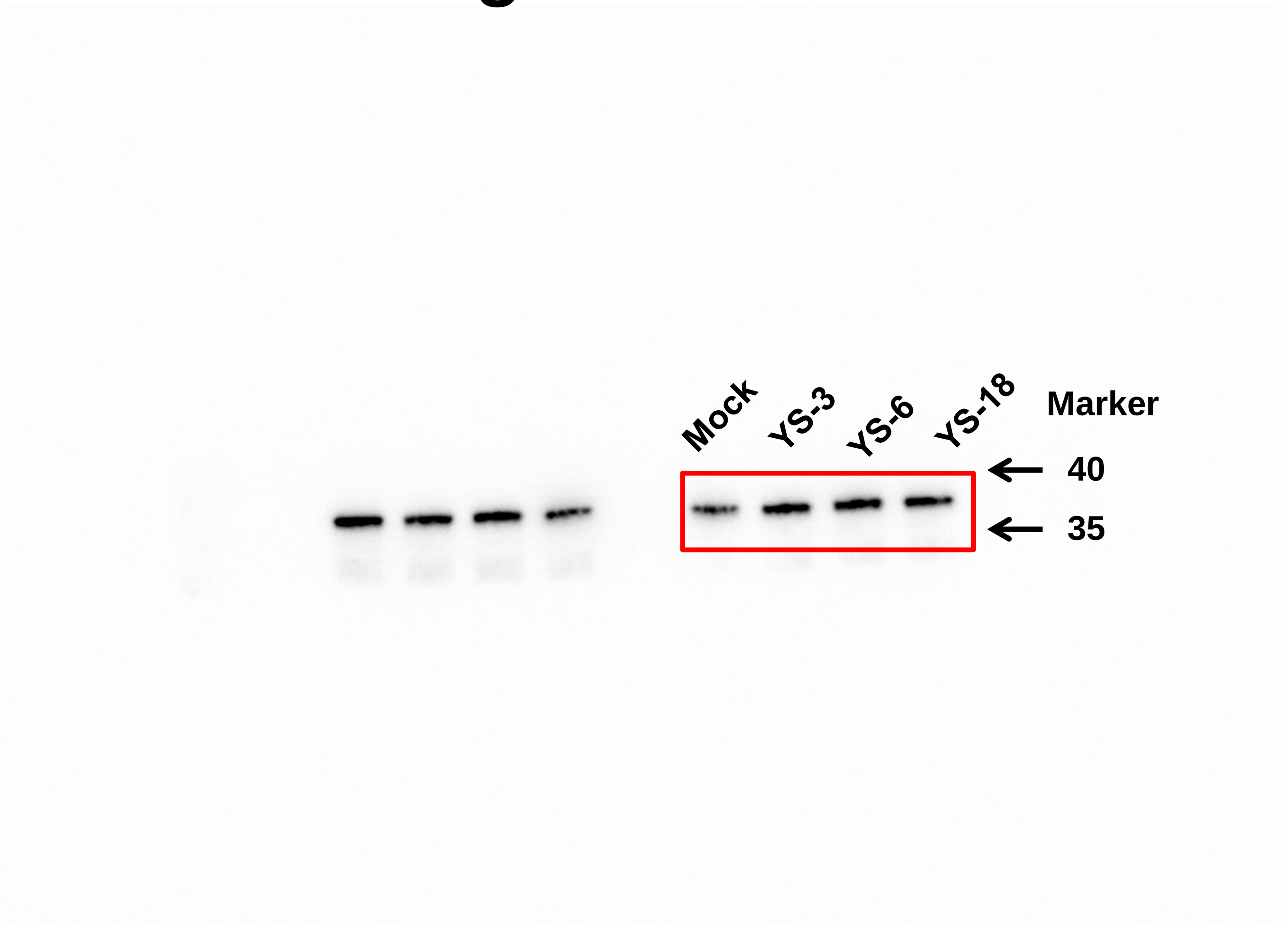

After the membrane was transferred, the membrane was cut from 35-40kd, and the part of the membrane on 35-40 kd was incubated with **GAPDH** antibody, and band detection was performed.

Figure S7f

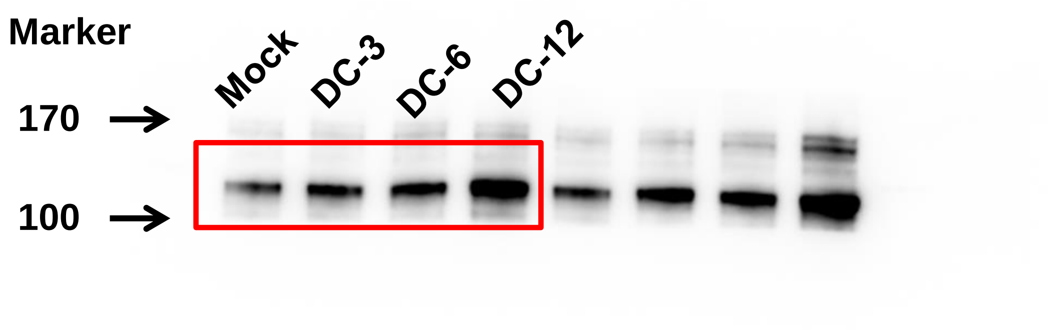

After the membrane was transferred, the membrane was cut from 100-170kd, and the part of the membrane on 100-170 kd was incubated with **ACTN4** antibody, and band detection was performed.

Figure S7f

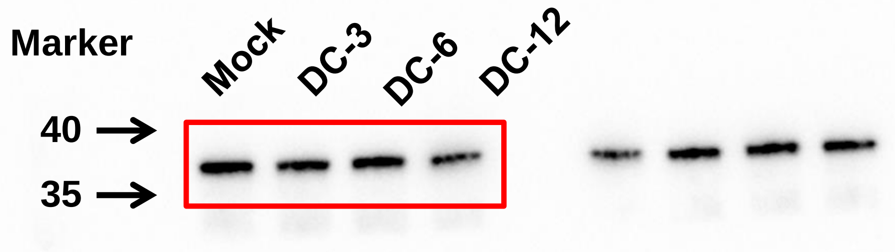

After the membrane was transferred, the membrane was cut from 35-40kd, and the part of the membrane on 35-40 kd was incubated with **GAPDH** antibody, and band detection was performed.

Figure S8e

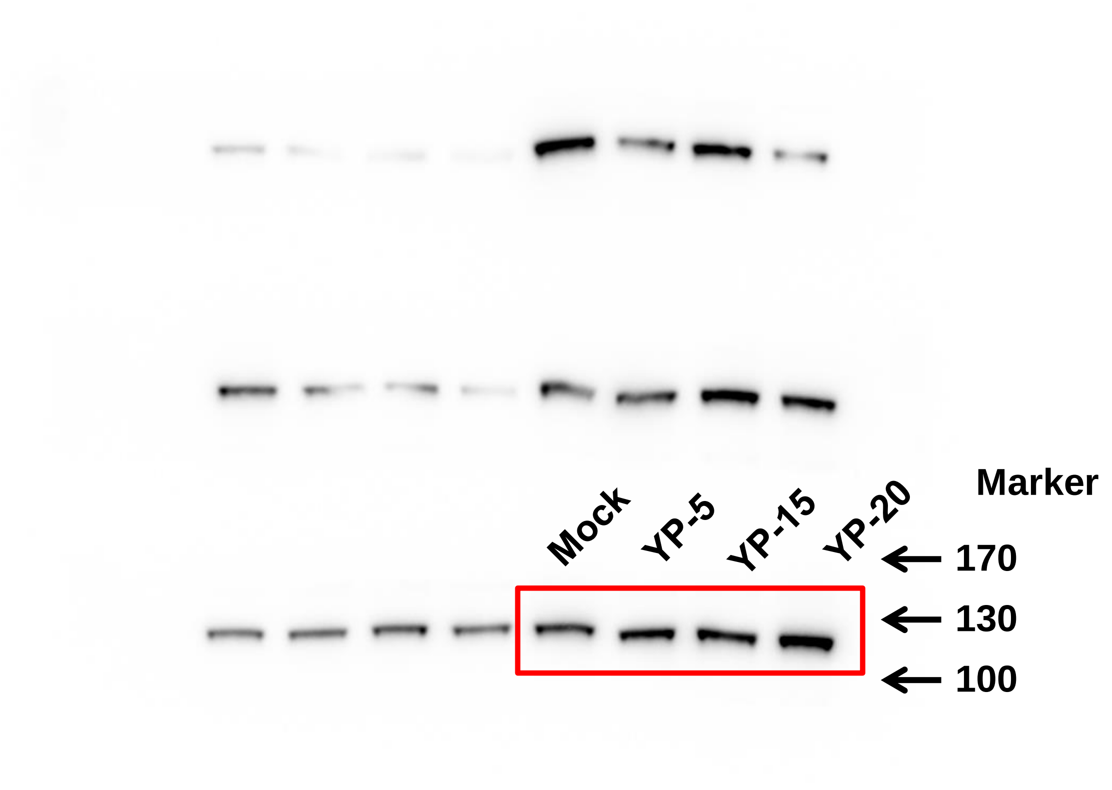

After the membrane was transferred, the membrane was cut from 100-170kd, and the part of the membrane on 100-170 kd was incubated with **ACTN4** antibody, and band detection was performed.

Figure S8e

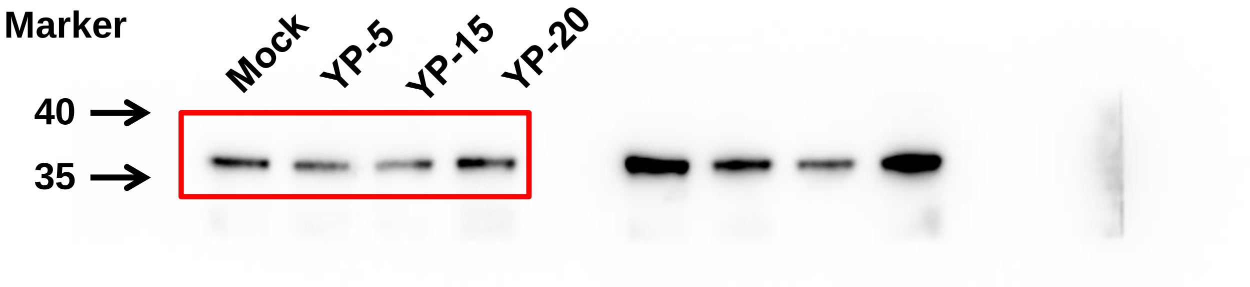

After the membrane was transferred, the membrane was cut from 35-40kd, and the part of the membrane on 35-40 kd was incubated with **GAPDH** antibody, and band detection was performed.

Figure S8f

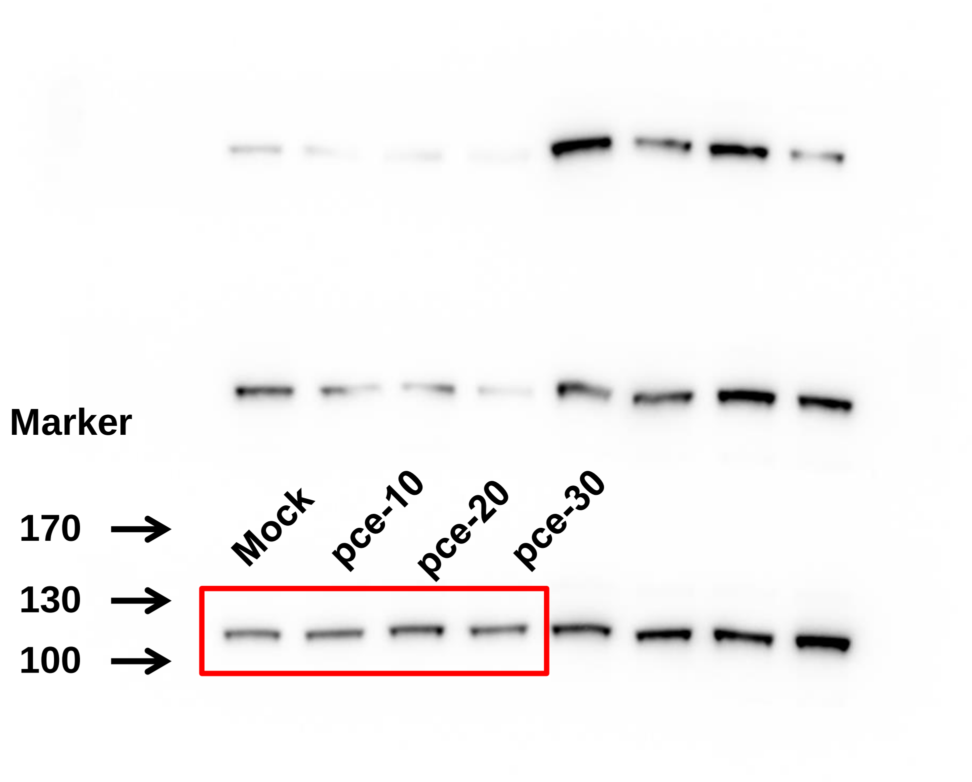

After the membrane was transferred, the membrane was cut from 100-170kd, and the part of the membrane on 100-170 kd was incubated with **ACTN4** antibody, and band detection was performed.

Figure S8f

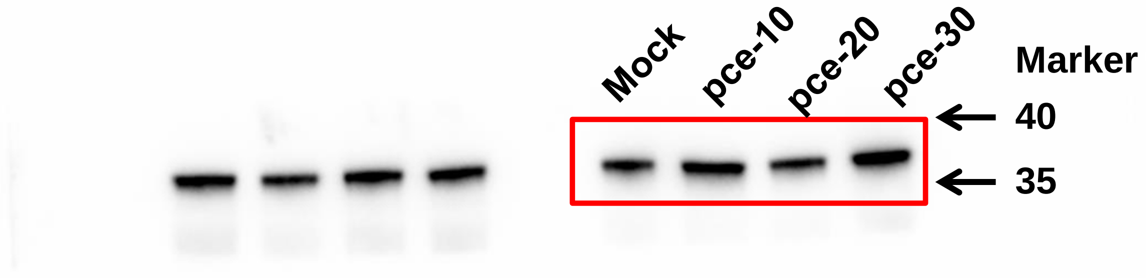

After the membrane was transferred, the membrane was cut from 35-40kd, and the part of the membrane on 35-40 kd was incubated with **GAPDH** antibody, and band detection was performed.

Figure S9a

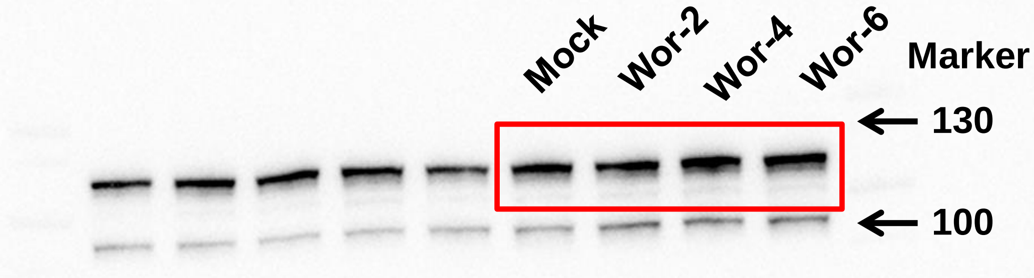

After the membrane was transferred, the membrane was cut from 100-130kd, and the part of the membrane on 100-130 kd was incubated with **ACTN4** antibody, and band detection was performed.

Figure S9a

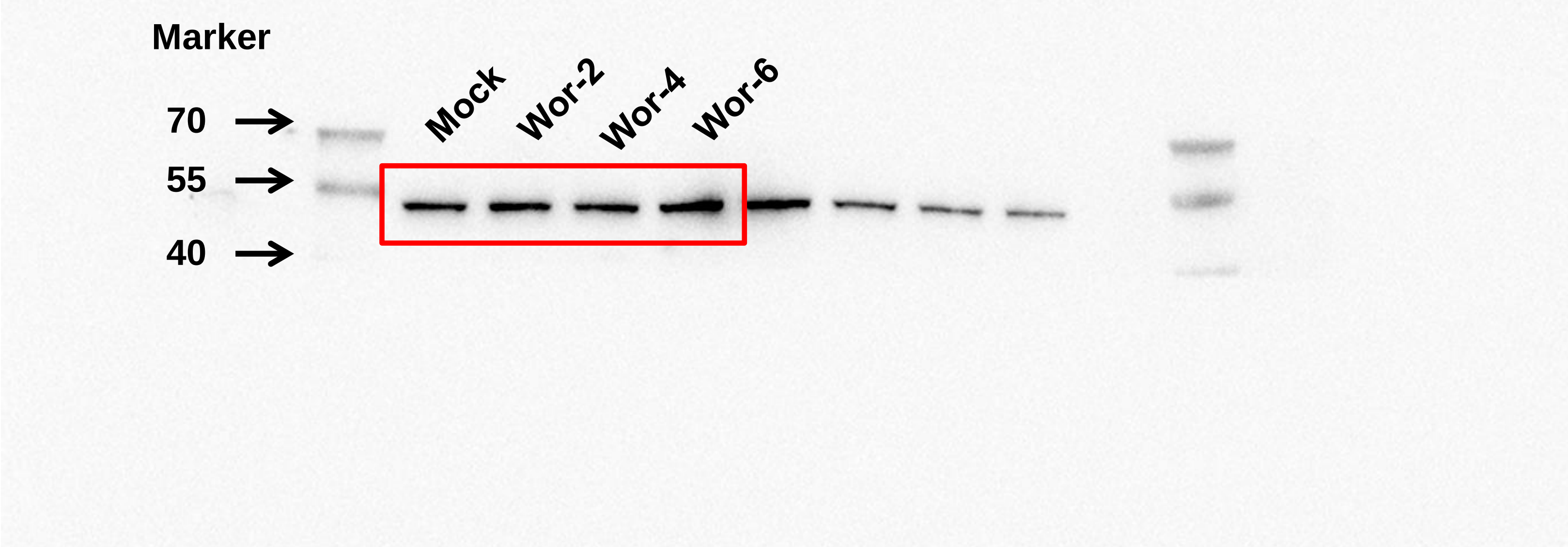

After the membrane was transferred, the membrane was cut from 40-70kd, and the part of the membrane on 40-70 kd was incubated with **SARS-CoV-2 Nucleoprotein Rabbit pAb** antibody, and band detection was performed.

Figure S9a

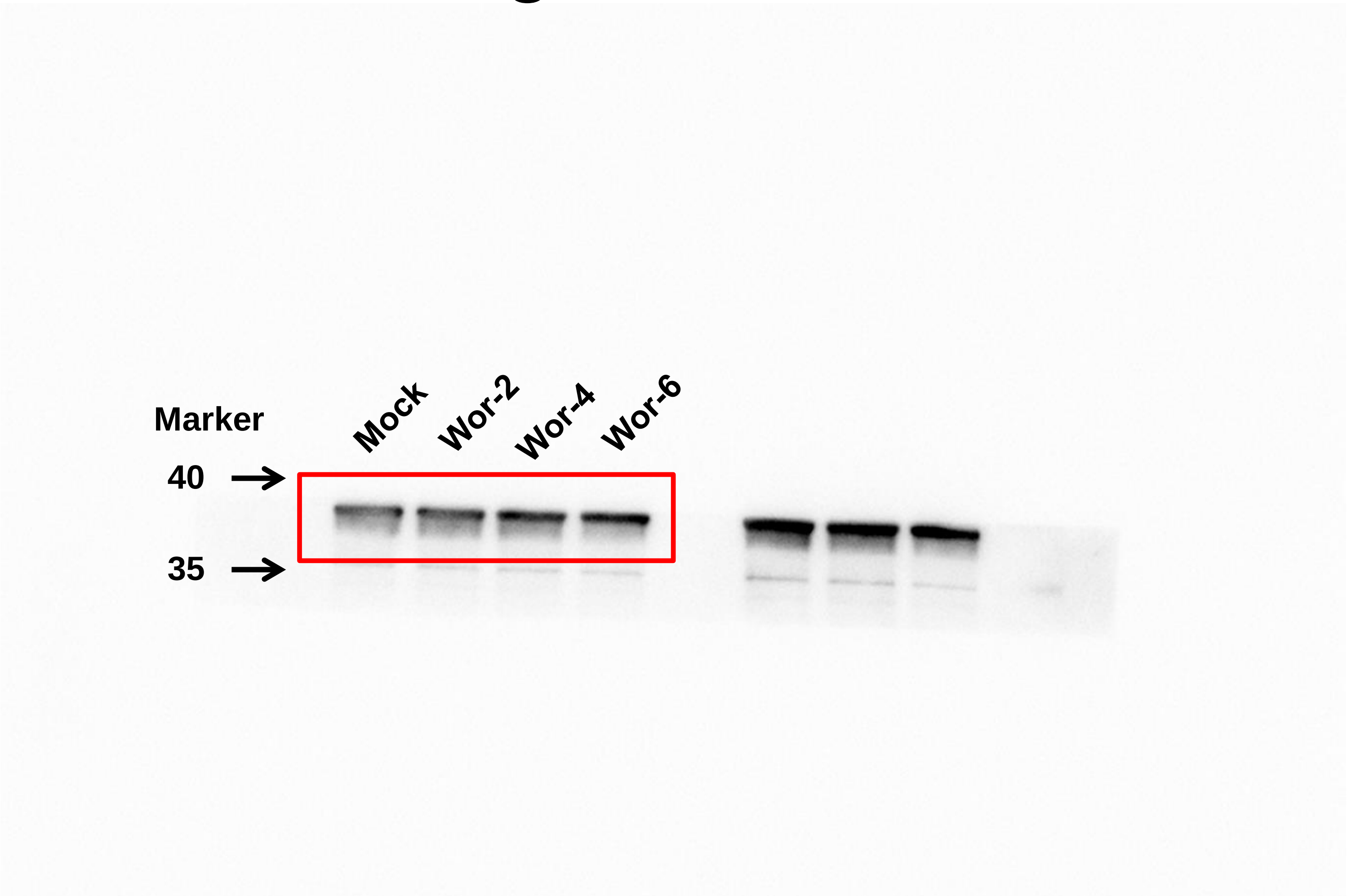

After the membrane was transferred, the membrane was cut from 35-40kd, and the part of the membrane on 35-40 kd was incubated with **GAPDH** antibody, and band detection was performed.

Figure S9b

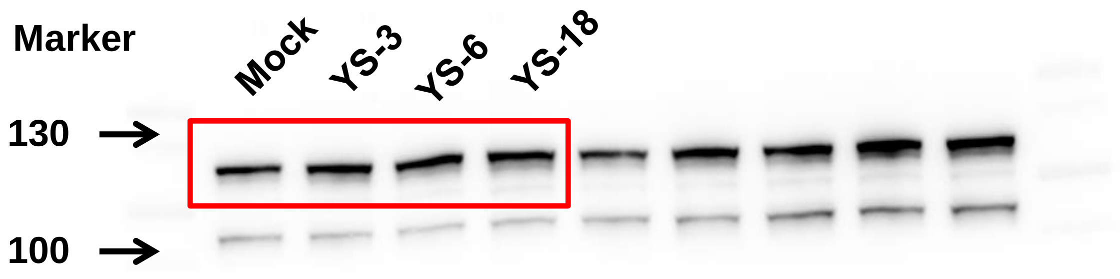

After the membrane was transferred, the membrane was cut from 100-130kd, and the part of the membrane on 100-130 kd was incubated with **ACTN4** antibody, and band detection was performed.

Figure S9b

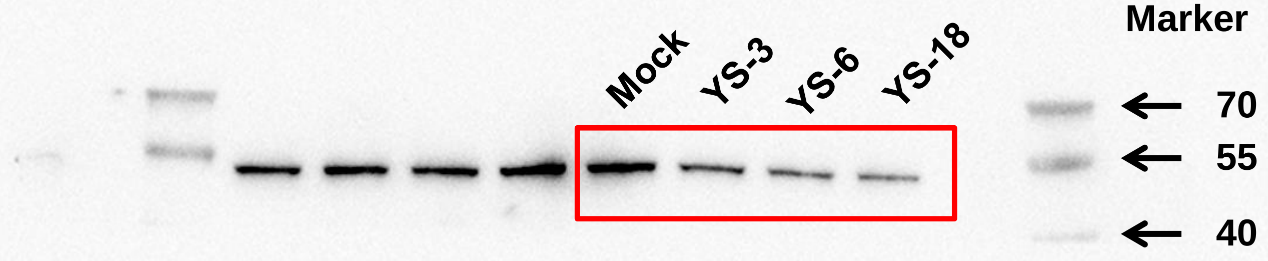

After the membrane was transferred, the membrane was cut from 40-70kd, and the part of the membrane on 40-70 kd was incubated with **SARS-CoV-2 Nucleoprotein Rabbit pAb** antibody, and band detection was performed.

Figure S9b

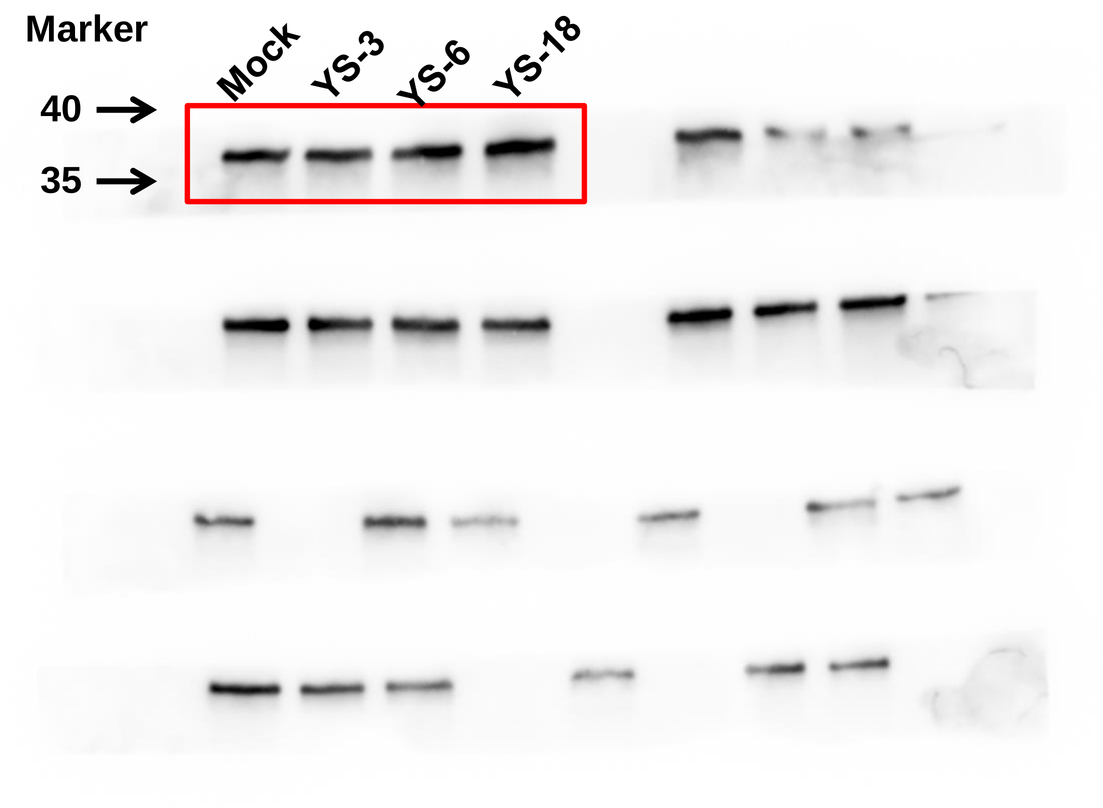

After the membrane was transferred, the membrane was cut from 35-40kd, and the part of the membrane on 35-40 kd was incubated with **GAPDH** antibody, and band detection was performed.

Figure S9c

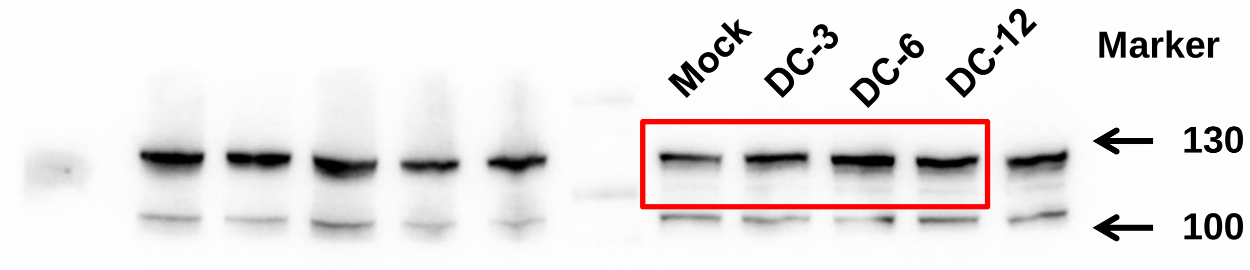

After the membrane was transferred, the membrane was cut from 100-130kd, and the part of the membrane on 100-130 kd was incubated with **ACTN4** antibody, and band detection was performed.

Figure S9c

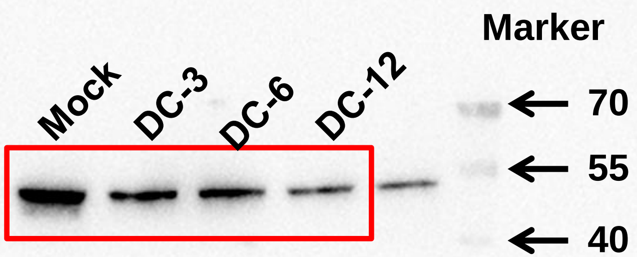

After the membrane was transferred, the membrane was cut from 40-55kd, and the part of the membrane on 40-55 kd was incubated with **SARS-CoV-2 Nucleoprotein Rabbit pAb** antibody, and band detection was performed.

Figure S9c

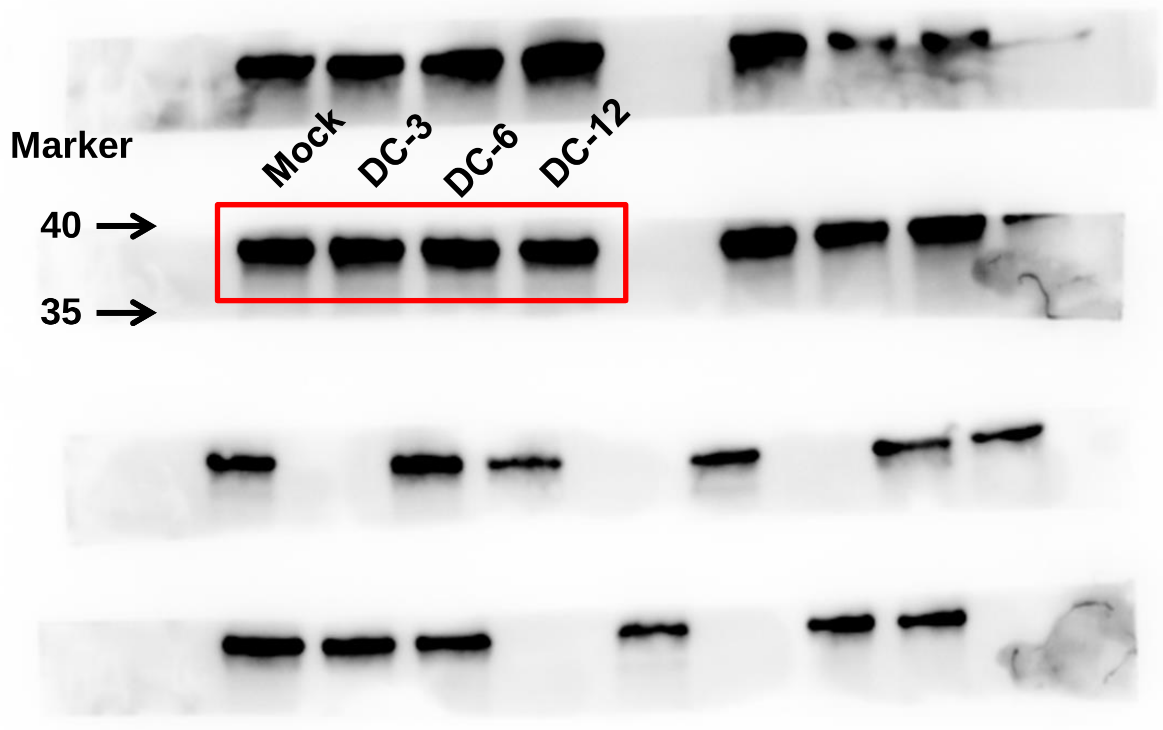

After the membrane was transferred, the membrane was cut from 35-40kd, and the part of the membrane on 35-40 kd was incubated with **GAPDH** antibody, and band detection was performed.
